# Aged lipid‐laden microglia display impaired responses to stroke

**DOI:** 10.15252/emmm.202217175

**Published:** 2022-12-21

**Authors:** Maria Arbaizar‐Rovirosa, Jordi Pedragosa, Juan J Lozano, Carme Casal, Albert Pol, Mattia Gallizioli, Anna M Planas

**Affiliations:** ^1^ Department of Neuroscience and Experimental Therapeutics, Instituto de Investigaciones Biomédicas de Barcelona (IIBB) Consejo Superior de Investigaciones Científicas (CSIC) Barcelona Spain; ^2^ Institut d'Investigacions Biomèdiques August Pi i Sunyer (IDIBAPS) Barcelona Spain; ^3^ Bioinformatics Platform Centro de Investigación Biomédica en Red Enfermedades Hepáticas y Digestivas (CIBEREHD) Barcelona Spain; ^4^ Microscopy Service, Instituto de Investigaciones Biomédicas de Barcelona (IIBB) Consejo Superior de Investigaciones Científicas (CSIC) Barcelona Spain; ^5^ Department of Biomedical Sciences, Faculty of Medicine Universitat de Barcelona Barcelona Spain; ^6^ Institució Catalana de Recerca i Estudis Avançats (ICREA) Barcelona Spain

**Keywords:** brain, immunometabolism, ischemia, lipid droplets, microglia, Cardiovascular System, Immunology, Neuroscience

## Abstract

Microglial cells of the aged brain manifest signs of dysfunction that could contribute to the worse neurological outcome of stroke in the elderly. Treatment with colony‐stimulating factor 1 receptor antagonists enables transient microglia depletion that is followed by microglia repopulation after treatment interruption, causing no known harm to mice. We tested whether this strategy restored microglia function and ameliorated stroke outcome in old mice. Cerebral ischemia/reperfusion induced innate immune responses in microglia highlighted by type I interferon and metabolic changes involving lipid droplet biogenesis. Old microglia accumulated lipids under steady state and displayed exacerbated innate immune responses to stroke. Microglia repopulation in old mice reduced lipid‐laden microglia, and the cells exhibited reduced inflammatory responses to ischemia. Moreover, old mice with renewed microglia showed improved motor function 2 weeks after stroke. We conclude that lipid deposits in aged microglia impair the cellular responses to ischemia and worsen functional recovery in old mice.

## Graphical abstract

Stroke outcome is impaired in old subjects. Here, microglia of aged mice are shown to accumulate lipids and display exacerbated inflammation after stroke. The microglia lipid droplet content was reduced by microglia depletion/repopulation in old mice, and motor function was improved after stroke.

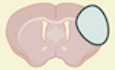

**The paper explained**

**Problem**

Over 12 million people suffer a new stroke every year in the world. Stroke is the second‐leading cause of mortality and a prominent cause of permanent disability worldwide. Age is a nonmodifiable stroke risk factor, and stroke outcome is worse in aged individuals. Novel strategies for the restoration of cerebral blood flow by removing or dissolving the arterial blood clots have improved dramatically the treatment of ischemic stroke. However, these interventions are only useful in the hours that follow stroke onset and within the first day at the most. Beyond this, no drugs have shown efficacy to improve stroke disability so far. Innovative therapeutic strategies are needed to reduce the disability burden of stroke sufferers.

**Results**

Our study focused on microglia, which is a population of immune cells resident in the brain. After stroke, these cells carry out important functions like removing the damaged tissue. To this end, these cells suffer metabolic and immune changes, and we noticed that they accumulate little organelles of fat, called lipid droplets. Moreover, we found that microglia of old but healthy mice do also have lipid droplets. After stroke, old mice show larger brain lesions and worse neurological outcomes, and their microglia show a more inflammatory profile. We argued that microglia of old mice may be dysfunctional and hypothesized that renewing this cell population may be beneficial. We used a pharmacological strategy to deplete and then repopulate the brain microglia of old mice. Notably, the renewed microglia showed less lipid droplets, and after stroke, the old mice showed a better recovery of motor function.

**Impact**

Our experimental findings show the involvement of lipid droplets in the response of microglia to stroke and suggest that lipid disposal may be impaired in microglia of aged individuals. Altered lipid metabolism in the elderly may deteriorate the cell functions and may contribute to worsen stroke outcome. Strategies preventing cellular lipid accumulations in aging may preserve microglia function and increase the resilience to stroke.

## Introduction

Age is the main nonmodifiable stroke risk factor, and the stroke outcome is worse in the elderly. The higher vulnerability of the aged brain to ischemia has been attributed to differences in the mechanisms of ischemic brain injury between aged and young individuals (Chen *et al*, 2010). Moreover, elderly patients with stroke show worse responses to reperfusion therapies (Mishra *et al*, 2010). Several lines of evidence support that exacerbated brain inflammation and altered immunological response underlie the worse neurological impairment in aged subjects after cerebral ischemia/reperfusion (Ritzel *et al*, 2018). The brain of aged mice shows a differential transcriptional profile to that of the young (Androvic *et al*, 2020). The expression of genes encoding pro‐inflammatory and innate immune response molecules is exaggerated in the elderly, whereas genes involved in axonal and synaptic structure and activity are downregulated (Hickman *et al*, 2013; Hammond *et al*, 2019; Androvic *et al*, 2020; Tabula Muris Consortium, 2020). Aging impairs microglia functions (Sierra *et al*, 2007) particularly affecting white matter‐associated microglia (Cantuti‐Castelvetri *et al*, 2018; Safaiyan *et al*, 2021). Recent studies identified subsets of dysfunctional microglia in the aging brain characterized by increased oxidative stress, inflammatory profile, abnormal lipid accumulation (Marschallinger *et al*, 2020), and increased lysosomal storage (Burns *et al*, 2020).

Microglial cells play critical roles in the central nervous system during brain development (Wu *et al*, 2015) and in adulthood to maintain brain homeostasis via microglia–neuron interactions (Cserép *et al*, 2020, 2021). Microglia prevent excessive neuronal depolarization following excitotoxic insults (Kato *et al*, 2016) and exert some protective effects in cerebral ischemia (Szalay *et al*, 2016; Otxoa‐de‐Amezaga *et al*, 2019; Marino Lee *et al*, 2021). Microglia survival is dependent on colony‐stimulating factor 1 receptor (CSF1R). In rodents, genetic strategies to prevent microglial *Csf1r* expression or treatment with pharmacological inhibitors of CSF1R cause microglia depletion (Waisman *et al*, 2015). Upon removal of the CSF1R inhibitor, microglia repopulate the brain and several lines of evidence support that repopulated microglia exert beneficial effects (Elmore *et al*, 2018; Han *et al*, 2019). Proliferating and nonproliferating microglia with a distinct transcriptomic profile are detected in the adult mouse brain during repopulation following microglia depletion (Belhocine *et al*, 2021). Notably, microglia repopulation in the aged brain reversed some age‐induced changes in microglia gene expression, and reduced lysosome enlargement and lipofuscin accumulation (O'Neil *et al*, 2018). In an experimental model of traumatic brain injury, repopulating microglia stimulated neurogenesis and promoted recovery mediated by IL‐6‐dependent neuroprotection (Willis *et al*, 2020). We hypothesized that restoring the microglia phenotype in the elderly may improve the functional outcome of stroke. We investigated the microglia response to stroke in young (3–4 months) and aged (21–22 months) mice and studied whether microglia renewal improved stroke outcome in the elderly. Our work shows that drug‐induced microglia repopulation in the elderly modifies some phenotypic traits of old microglia and increases the resilience of old individuals to the motor impairment caused by stroke.

## Results

### Cerebral ischemia/reperfusion changes the transcriptomic profile and function of microglia

**Figure 1:**
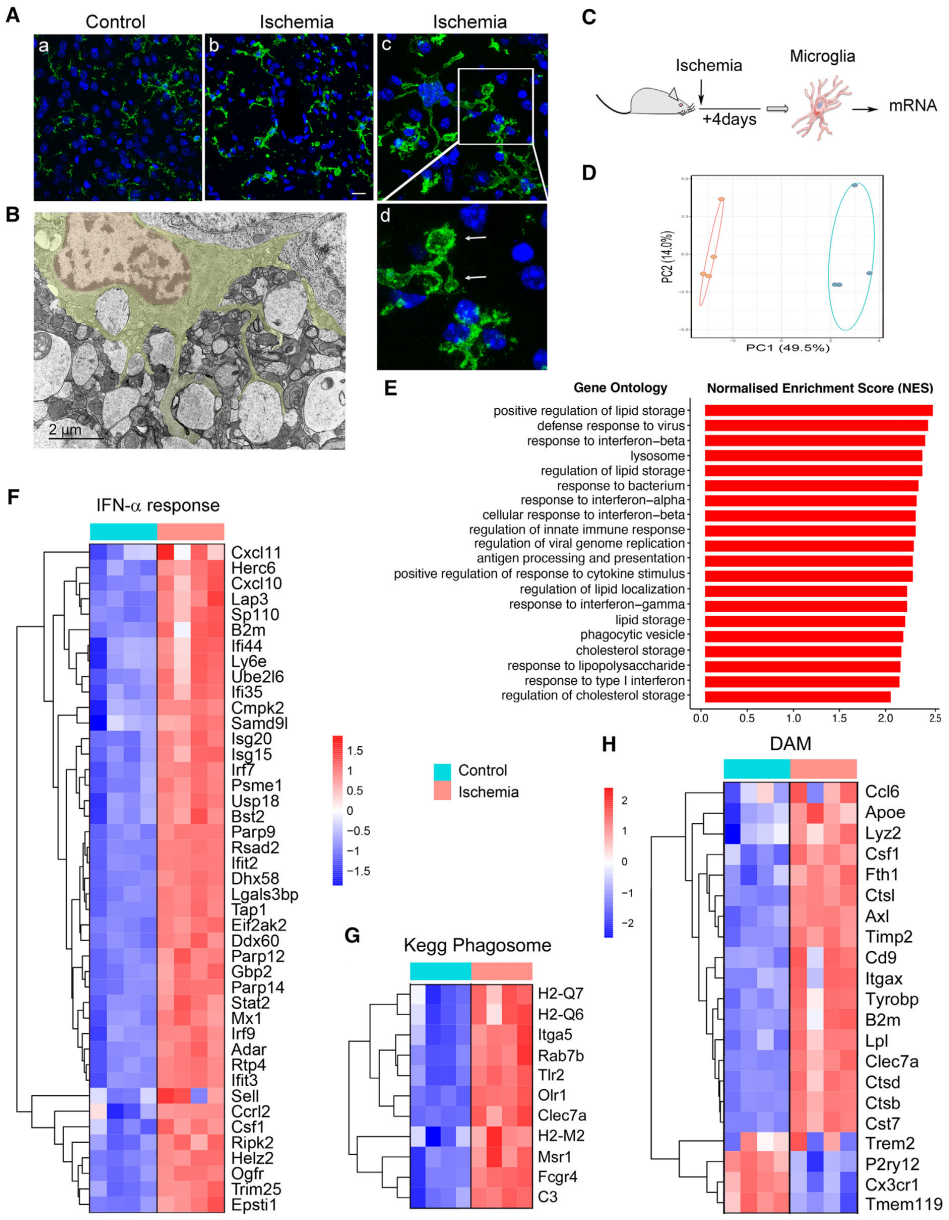
Transcriptomic and phenotypic changes in microglia after brain ischemia Representative P2YR12 immunostaining (green) of microglia of wild‐type male mice showing morphological differences between control microglia (a) and ischemic microglia (b–d). Ischemic microglial cells show typical phagocytic pouches (arrows). Nuclei are labeled with TO‐PRO‐3 (blue). Image (d) is a magnification of the area marked with a square in (c). Scale bar (a, b): 20 μm; (c): 10 μm; (d): 4 μm.Transmission electron microscopy showing a microglial cell at the periphery of infarction 1 day postischemia surrounding remarkably swollen postsynaptic vesicles. Scale bar: 2 μm.Microglia were obtained using fluorescence‐activated cell sorting (FACS) from the brain of control and ischemic young (3–4 months) male CX3CR1cre^ERT2^:Rosa26‐tdT mice 4 days postischemia (*n* = 4 mice per group). Microglia RNA was extracted for RNAseq analysis (GSE136856), as reported (Gallizioli *et al*, 2020).Principal components analysis (PCA) shows sample distribution clearly separating microglia from control and ischemic mice.Gene Ontology analysis illustrating pathways enriched in microglia after brain ischemia.GSEA highlights the IFN‐α response pathway as highly upregulated after ischemia.Genes of the Kegg pathway: *Phagosomes* are upregulated in microglia after ischemia. Color scale as in (F).Most genes described as upregulated or downregulated in disease‐associated microglia (DAM) are accordingly regulated in microglia 4 days postischemia.

Microglia undergo strong phenotypic alterations due to brain injury following stroke. In line with the need to remove dead cells and cell debris, microglia morphology became more reactive and displayed engulfing phagosomes (Fig 1A). In the periphery of infarction, electron microscopy showed microglia surrounding swollen synaptic vesicles (Fig 1B). We studied the transcriptomic profile of microglia obtained via fluorescence‐activated cell sorting (FACS) from the brain of mice after an episode of transient ischemia induced by 45‐min intraluminal middle cerebral artery occlusion (MCAo) followed by reperfusion (Fig 1C), as we previously reported (Gallizioli *et al*, 2020). RNAseq analysis showed marked changes in the microglia transcriptomic profile 4 days postischemia versus controls (Fig 1D). According to gene ontology (GO) pathways and gene set enrichment analysis (GSEA), ischemia upregulated innate immune pathways in microglia with prominent enrichment of genes regulating lipid storage and defense response to virus, particularly interferon (IFN)‐α and IFN‐β (Fig 1E and F). Other biological processes highly enriched in ischemic microglia included terms related to phagocytosis, lysosomes, and cholesterol storage (Fig 1G). Notably, some of the abovementioned ischemia‐induced differentially expressed genes (DEGs) in microglia are typically reported in disease‐associated microglia (DAM) under diverse neurodegenerative conditions (Keren‐Shaul *et al*, 2017; Deczkowska *et al*, 2018). Expression of the DAM genes was similarly upregulated or downregulated in ischemic microglia. Therefore, microglia acquire features of a DAM‐like profile within hours/days of an acute ischemic insult (Fig 1H).

### Ischemia induces the formation of lipid droplets in microglia

**Figure 2:**
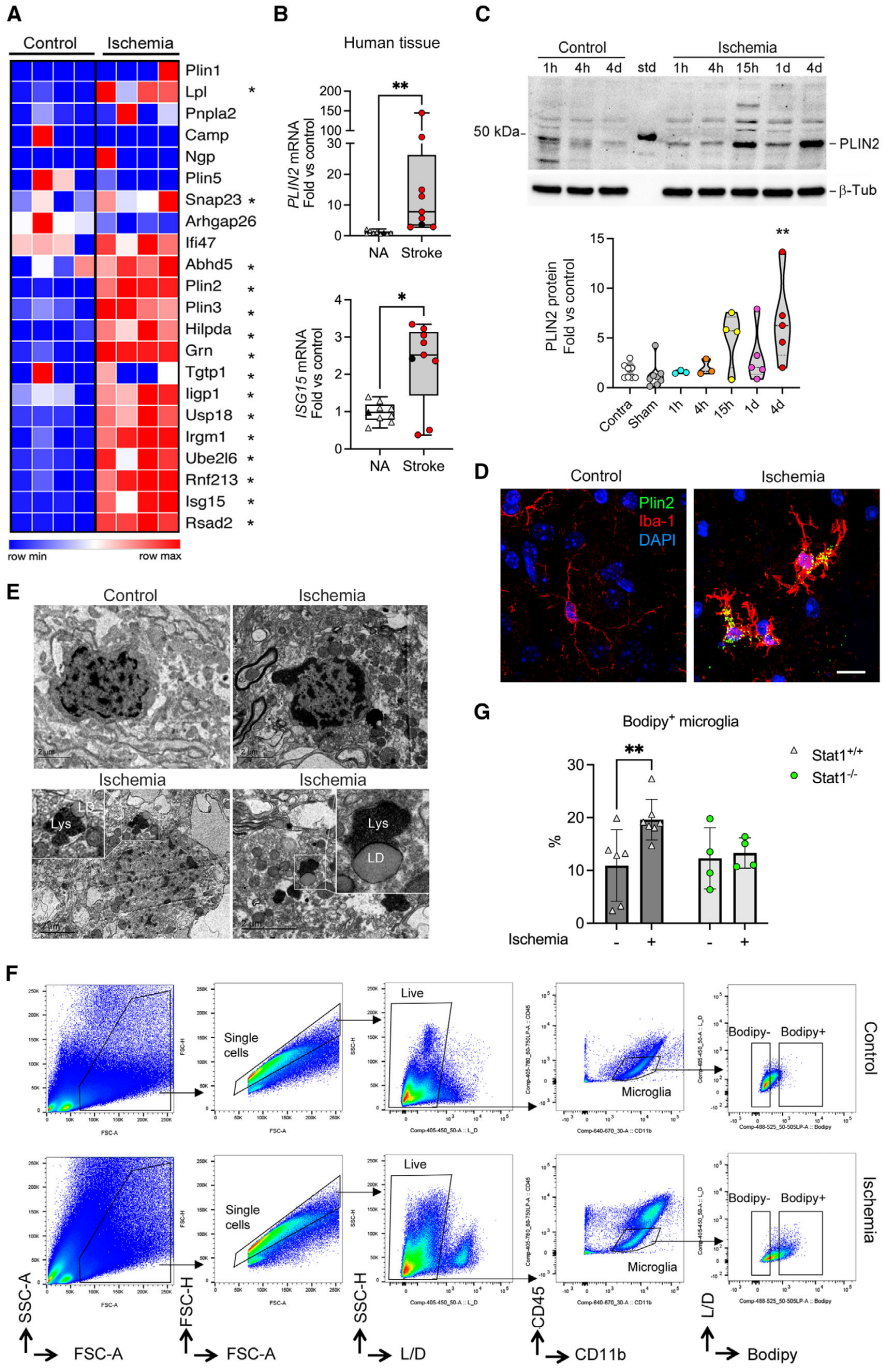
Microglia accumulate lipid droplets after brain ischemia Expression of a selection of genes encoding lipid droplet‐associated proteins in microglia sorted from control (*n* = 4) and ischemic  (*n* = 4) male mice at day four postischemia (obtained from RNAseq data shown in Fig 1). Heatmap illustrates ischemia‐induced upregulation of genes marked with * indicating adjusted *P*‐value < 0.001.mRNA expression of *PLIN2* and *ISG15* in postmortem human brain tissue of nine stroke patients (points are values of individual patients); eight women (red points) and one man (black point). Samples were obtained from the ischemic tissue (Stroke) and nonaffected (NA) tissue. The graph shows boxes from the 25^th^ to 75^th^ percentiles, the median line, and whiskers down to the minimum and up to the maximum value, showing all points. Values are expressed as fold versus mean control (i.e., nonaffected tissue) Wilcoxon matched‐pairs signed‐rank test, **P = 0.0039; *P = 0.0195.PLIN2 protein expression in mouse brain tissue, as assessed by Western blotting. Values were obtained from the ipsilateral hemisphere 1 h (*n* = 3), 4 h (*n* = 3), 15 h (*n* = 4), 24 h (*n* = 5) and 96 h (*n* = 5) postischemia, and 15 h (*n* = 4), 24 h (*n* = 2), and 96 h (*n* = 2) after sham operation. Values of sham mice were pooled together since they did not differ between time points. Samples of the contralateral hemisphere (Contra) of ischemic mice (1 h, *n* = 2; 24 h, *n* = 3; and 96 h, *n* = 3) were also evaluated. Ischemia increased PLIN2 expression at day 4 versus the sham group (**P = 0.0032, Kruskal–Wallis test and Dunn's multiple comparisons test). β‐tubulin is the protein loading control. The “Std” lane indicates the molecular weight standard. Quantification of band intensity where values are expressed as fold versus control (nonischemic). Points correspond to independent male mice and group values are expressed as a violin plot. Values were obtained 1 h (*n* = 3), 4 h (*n* = 3), 15 h (*n* = 4), 24 h (*n* = 5), and 96 h (*n* = 5) postischemia, and 15 h (*n* = 4), 24 h (*n* = 2), and 96 h (*n* = 2) after sham operation. Values of sham mice were pooled together since they did not differ between time points. Samples of the contralateral hemisphere (Contra) of ischemic mice (1 h, *n* = 2; 24 h, *n* = 3; and 96 h, *n* = 3) were also evaluated. Ischemia increased PLIN2 expression at day 4 (**P = 0.0032, Kruskal–Wallis test and Dunn's multiple comparisons test versus the sham group). Data are presented as violin plots with lines at the median and quartiles (dashed lines).Immunofluorescence with antibodies against PLIN2 (green) and Iba‐1 (red) in brain tissue 1 day after induction of ischemia in female mice. Nuclei are stained with DAPI (blue). Control is the contralateral hemisphere. Scale bar: 10 μm.Transmission electron microscopy showing microglial cells in control or ischemic tissue 1 day postischemia. Ischemia induces the formation of lipid droplets (LD) in microglia. LD are often seen near lysosomes (Lys). Insets in the lower panels are magnifications of the regions marked with a square. Scale bar 2 μm.Flow cytometry gates to identify Bodipy^+^ microglia for control and ischemic brain tissue. Gates were set based on fluorescence minus one (FMO) intensity values.Quantification of the percentage of CD11b^+^CD45^low^ microglia containing lipid droplets as Bodipy^+^ microglia using flow cytometry in male mice deficient in Stat1 (Stat1^−/−^; *n* = 4) and corresponding Stat1^+/+^ mice (*n* = 7) shows ischemia‐induced increases in Stat1^+/+^ mice (**P = 0.0086) but not in Stat1^−/−^ mice (*P* = 0.927; Two‐way ANOVA and Šídák's multiple comparisons test). The graph shows values of the nonischemic (−) and ischemic (+) brain hemispheres of individual mice and the mean ± SD.

Conditions involving infection or inflammation in certain cell types cause the accumulation of neutral lipids in the cytoplasm forming lipid droplets. Interestingly, lipid droplets are surrounded by a monolayer of phospholipids decorated with innate immune molecules, particularly proteins regulated by type I IFN that play a critical role in immune defense (Bosch *et al*, 2020; Monson *et al*, 2021). Given the conspicuous type I IFN response induced by cerebral ischemia in microglial cells, as well as the accompanying inflammatory response and enrichment in genes regulating lipid storage, we hypothesized that ischemia could induce lipid droplet biogenesis in microglia. We checked for ischemia‐induced DEGs encoding typical lipid droplet membrane protein components, such as PLIN1‐5 family, and the reported IFN‐induced lipid droplet‐associated proteins (Bosch *et al*, 2020). Brain ischemia in young mice increased at day 4 the microglial mRNA expression of Adipophilin (*Plin2*), Perilipin (*Plin3*), Hypoxia‐Inducible Lipid Droplet‐Associated (*Hilpda*), granulin precursor (*Grn*), Abhydrolase Domain Containing 5, Lysophosphatidic Acid Acyltransferase (*Abhd5*), Synaptosome Associated Protein 23 (*Snap23*), and lipoprotein lipase (*Lpl*; Fig 2A). Moreover, we detected ischemia‐induced microglial mRNA upregulation of IFN‐induced lipid droplet‐associated molecules such as ISG15 Ubiquitin‐Like Modifier (*Isg15*), Ubiquitin‐Conjugating Enzyme E2 L6 (*Ube2L6*), Ubiquitin‐Specific Peptidase 18 (*Usp18*), Viperin (*Rsad2*), Ring Finger Protein 213 (*Rnf213*), Immunity‐Related GTPase M (*Irgm1*), and Interferon‐Inducible GTPase 1 (*Iigp1*; Fig 2A). We investigated whether this response was also upregulated after human stroke using postmortem brain tissue. Characteristics of the stroke patients are shown in Table EV1. Stroke increased the mRNA expression of *PLIN2* and *ISG15*, one of the typical type I IFN responsive genes, suggesting that the molecular machinery related to lipid droplet biogenesis was activated after stroke in the human brain too (Fig 2B). We then validated the ischemia‐induced expression of PLIN2 at the protein level by Western blotting (Fig 2C) and immunofluorescence (Fig 2D) in mouse tissue.

We identified the presence of lipid droplets in microglia by transmission electron microscopy of mouse brain tissue 24 h after ischemia (Fig 2E). Furthermore, we observed lipid droplets in contact with lysosomes, supporting the occurrence of macrolipophagy as a mechanism that could supply energy to the cells. The presence of lipid droplets in ischemic microglia was quantified by flow cytometry after staining the cells with fluorescent Bodipy (Fig 2F). The percentage of Bodipy^+^ CD45^low^CD11b^+^ microglia increased 4 days postischemia in Stat1^+/+^ mice, but not in Stat1^−/−^ mice (Fig 2G). Stat1 is a critical factor mediating the transduction of cellular responses to several types of IFNs (Van Boxel‐Dezaire *et al*, 2006). Therefore, these results show that the IFN response is involved in ischemia‐induced lipid droplet biogenesis in microglia.

### Old mice show worse neurological outcomes than young mice, and microglia of old mice show exacerbated innate immune responses to brain ischemia

**Figure 3:**
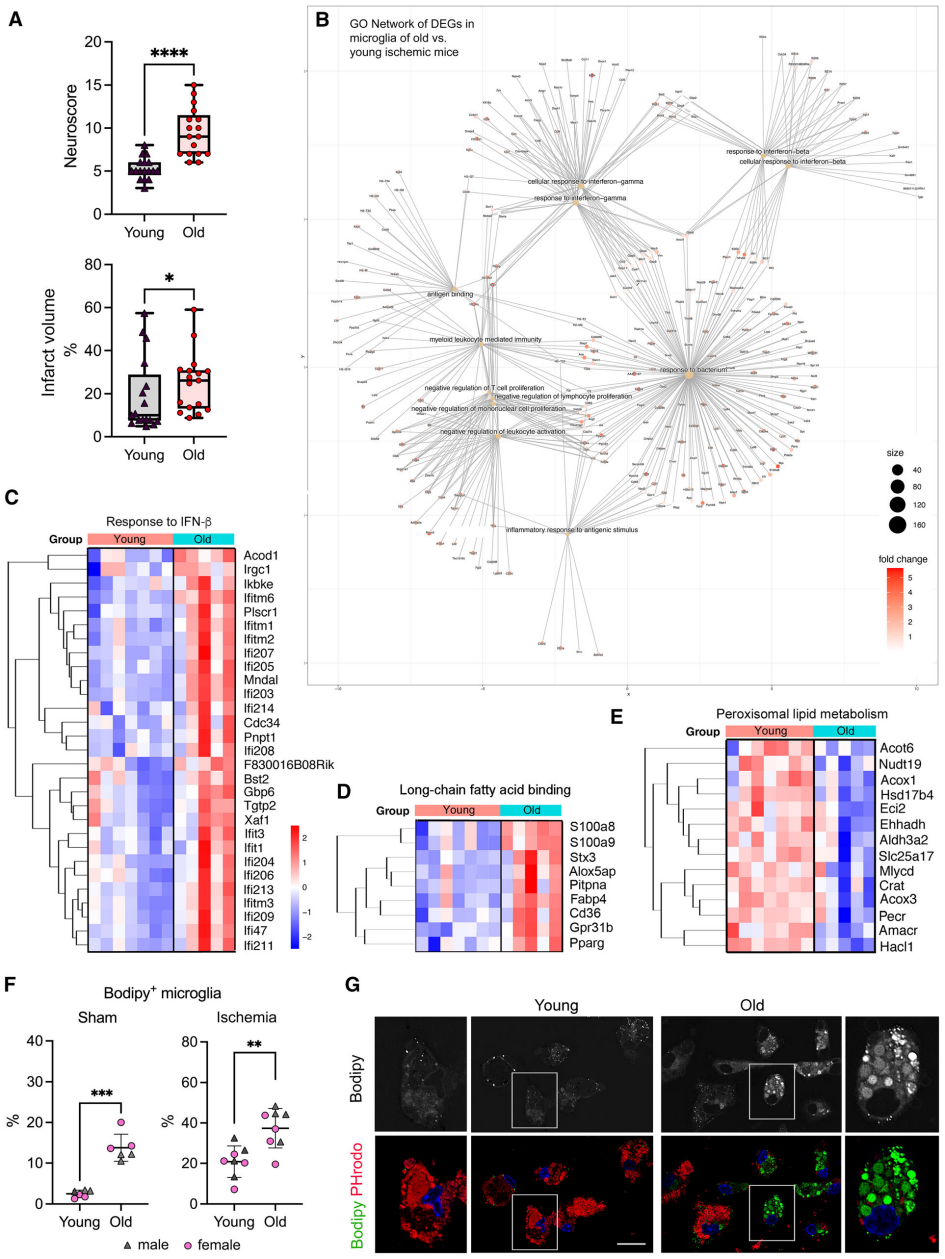
Worse stroke outcome in old mice and corresponding transcriptional profile of microglia AIschemia was induced in young and old female C57BL/6 mice (*n* = 17 per group) and infarct volume, as assessed with T2w MRI, and the neurological score was evaluated at day 4. The neurological score was higher (worse) in old mice (Mann–Whitney test, ****P < 0.0001), which also showed larger infarct volume (Mann–Whitney test, *P = 0.041) compared with young mice. The graph shows boxes from the 25^th^ to 75^th^ percentiles, the median line, and whiskers down to the minimum and up to the maximum value, showing all points.BMicroglia mRNA was obtained from young (3–4 months; *n* = 7) and old (21–22 months; *n* = 5) female mice 4 days postischemia and studied by RNAseq (GSE196737). Cnetplot illustrates the network of genes enriched after ischemia in old versus young mice linked to GO terms mainly related to innate immunity, inflammatory responses, and antigen presentation.CHeatmaps of genes of representative innate immunity pathways enriched in microglia of old versus young ischemic mice.D, EUpregulation of genes related to long‐chain fatty acid binding and downregulation of genes related to peroxisomal long‐chain fatty acid metabolism in microglia of old mice.FMicroglia obtained from young and old male (gray) and female (pink) mice 4 days postischemia (*n* = 16 mice; 4 mice per age group and sex) and sham operation (*n* = 12 mice; 3 mice per age group and sex) was studied by flow cytometry. After sham operation old mice showed a higher proportion of microglia containing lipid droplets after sham operation (***P < 0.001) and ischemia (**P = 0.0022; *t*‐test). Values show the mean ± SD.GMicroglia from adult young and old male mice using CD11b^+^ beads were kept in culture for 7 days and then exposed to red fluorescent pHrodo *Escherichia coli* bioparticles and stained with Bodipy. Bodipy^+^ lipid droplets (white in the upper panels ‐raw intensity‐ and green in the corresponding lower panels) are clearly seen in microglia from old mice, but not young mice. The images at the bottom also show phagocytosed red bioparticles, which are hardly seen in lipid droplets containing cells. The square in each image in the center is magnified in the images on the left and right sides for young and old microglia, respectively. Scale bar = 20 μm.

**Figure EV1:**
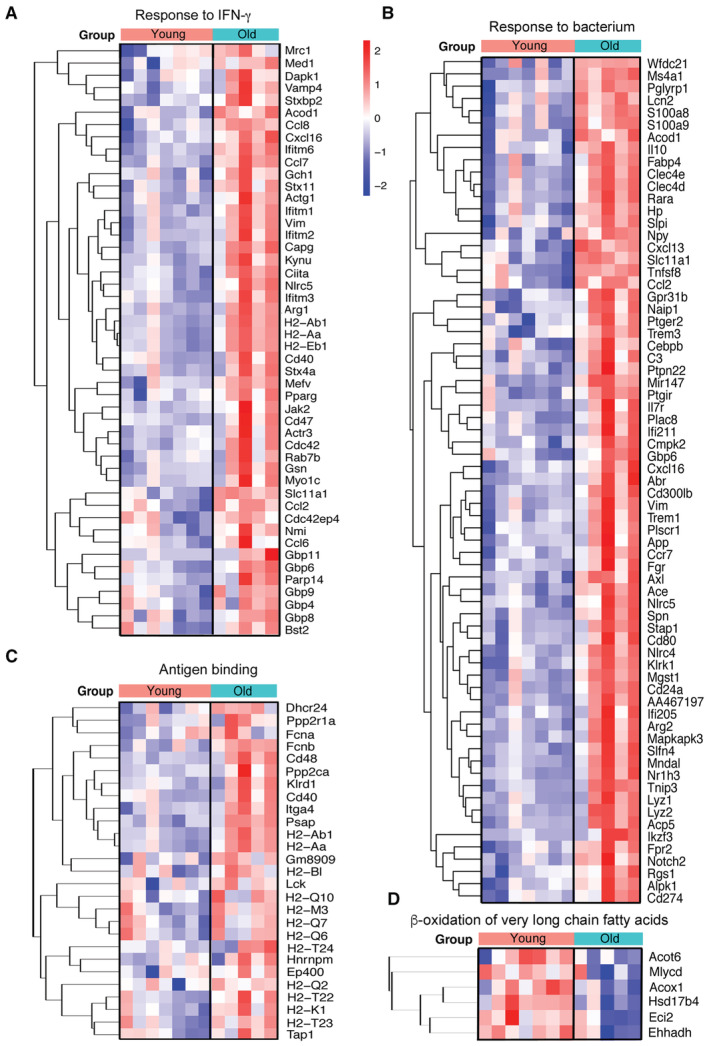
Enrichment of genes in GO pathways in microglia of old versus young ischemic female mice. Related to Fig 3 RNAseq analysis of microglia obtained by FACS from the brain of old (*n* = 5) versus young (*n* = 7) female mice 4 days after ischemia (GSE196737).
A–CHeatmaps illustrate genes upregulated in microglia of old ischemic mice versus young ischemic female mice for the following GO terms: “Response to IFN‐γ” (A), “Response to bacterium” (B), and “Antigen binding” (C).DBy contrast, downregulated pathways in microglia of old mice included: “β‐oxidation of very long‐chain fatty acids”.

Stroke caused more severe neurological deficits and larger infarct volumes in old (21–22 months) than in young (3–4 months) female mice (Fig 3A). Given the protective effect of estrogens (Koellhoffer & McCullough, 2013), it is possible that differences in plasma estrogen levels in individual young female mice (Appendix Fig S1), attributable to the various stages of the estrus cycle, contributed to infarct volume variability. Comparison of the transcriptomic profile of microglia FACS‐sorted from the brain 4 days postischemia in young and old female mice showed 1,980 DEGs. Of these, 1,153 genes (58%) were upregulated in old ischemic mice, and so were most GSEA pathways indicating a gain of function associated with age (Dataset EV1). Compared with microglia of young ischemic mice, microglia of old ischemic mice showed enrichment of GO pathways related to the innate immune system and antigen presentation, amongst others (Fig 3B). Enrichment of innate immune system and inflammatory pathways was highlighted by the GO terms “Response to interferon‐beta,” “Response to interferon‐gamma,” and “Response to bacterium” (Figs 3C and EV1). Many of these responses were already induced by ischemia in microglia of young mice (Fig 1E and F), but they were upregulated further in microglia of old mice. Inflammatory responses were accompanied by upregulation of genes involved in the “Regulation of necroptotic cell death,” and microglia of ischemic old mice also showed a prominent enrichment of antigen‐presenting machinery (Fig EV1).

In addition, microglia of old ischemic mice were enriched in pathways affecting “Phagocytosis,” and processes involving lipids, e.g., “Long‐chain fatty acid binding” (Fig 3D), “Positive regulation of fatty acid metabolic process,” “Cellular response to lipid,” “Lipid transport.” By contrast, “Peroxisomal transport,” “Peroxisomal lipid metabolism,” and “Beta‐oxidation of very long‐chain fatty acids” pathways were downregulated in microglia of old ischemic mice (Figs 3E and EV1). Deficiency in peroxisomal β‐oxidation causes very long‐chain fatty acid accumulation as reported not only in the liver of aged subjects (Périchon & Bourre, 1995) but also in the brain of Alzheimer's disease patients (Kou *et al*, 2011). Therefore, fatty acid disposal appears to be impaired in microglia of aged mice versus microglia of young mice after brain ischemia.

For comparative purposes, we also looked for changes in microglia gene expression in old versus young mice 4 days after sham operation (Dataset EV2). Microglia of old versus young sham‐operated female mice showed upregulation of innate immune and pro‐inflammatory GO pathways, together with upregulation of GO pathways involved in lipid accumulation and metabolism, such as “Fatty acid binding,” “Cellular response to fatty acid,” “Triglyceride metabolic process,” “Lipid storage,” “Cholesterol storage,” “Regulation of fatty acid biosynthetic process,” “Lipoprotein particle binding,” and “Long‐chain fatty acid binding,” amongst others. Therefore, compared with young microglia, microglia of old mice show a pro‐inflammatory profile and altered lipid metabolism already in the absence of ischemia.

**Figure EV2:**
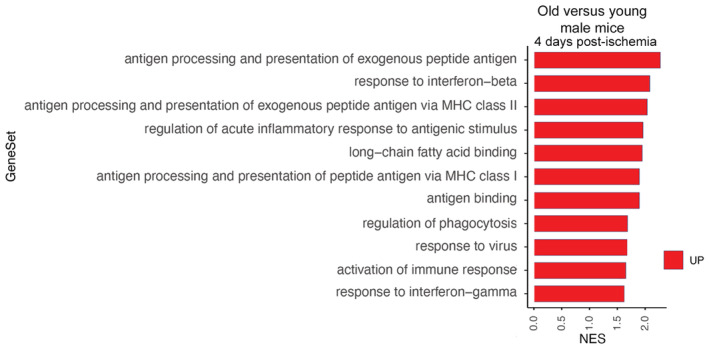
Enrichment of genes in GO pathways involved responses in microglia of old versus young ischemic male mice. Related to Fig 3 A transcriptomic analysis (GSE209732) was performed to validate that the main pathways found upregulated in old female mice were also upregulated in old male mice 4 days after ischemia. GO pathways following RNAseq analysis of microglia (Bodipy^+^) obtained by FACS from the brain of old (*n* = 3) versus young (*n* = 4) male mice 4 days after ischemia.

The results showing altered inflammatory and metabolic pathways in old versus young ischemic mice were obtained in female mice. Given that estrogens can affect microglia function and brain lipid metabolism (Vegeto *et al*, 2003; Morselli *et al*, 2018), we confirmed that many of the above‐described pathways were also upregulated in microglia of old versus young male mice after ischemia (Fig EV2, Dataset EV3).

These findings agree with the reported accumulation of lipid droplets in microglia of old mice under steady state that has been associated with impairment of microglia phagocytic function (Marschallinger *et al*, 2020). By flow cytometry we found that old male and female mice showed more lipid droplet‐rich microglia (Bodipy^+^) than young mice under sham and ischemic conditions (Fig 3F). Compared with sham operation, ischemia increased the percentage of lipid droplet‐rich microglia by 8.3‐fold in young mice and 2.7‐fold in old mice (Fig 3F). We then obtained microglia from the brain of young and old mice by immunomagnetic isolation and maintained the cells in culture for 7 days. We analyzed the cells by confocal microscopy after exposure to red pHrodo *Escherichia coli* bioparticles followed by Bodipy staining (Fig 3G). Bodipy^+^ microglia was only detected in the cultures obtained from old mice. Therefore, the culturing conditions did not remove the lipid droplets accumulated in microglia of old mice. Moreover, the microglia cells most enriched in lipid droplets did not contain or contained few phagocytosed bioparticles (Fig 3G), indicating that they display impaired phagocytosis.

### Microglia repopulation restores certain transcriptional features of old microglia after ischemia

**Figure EV3:**
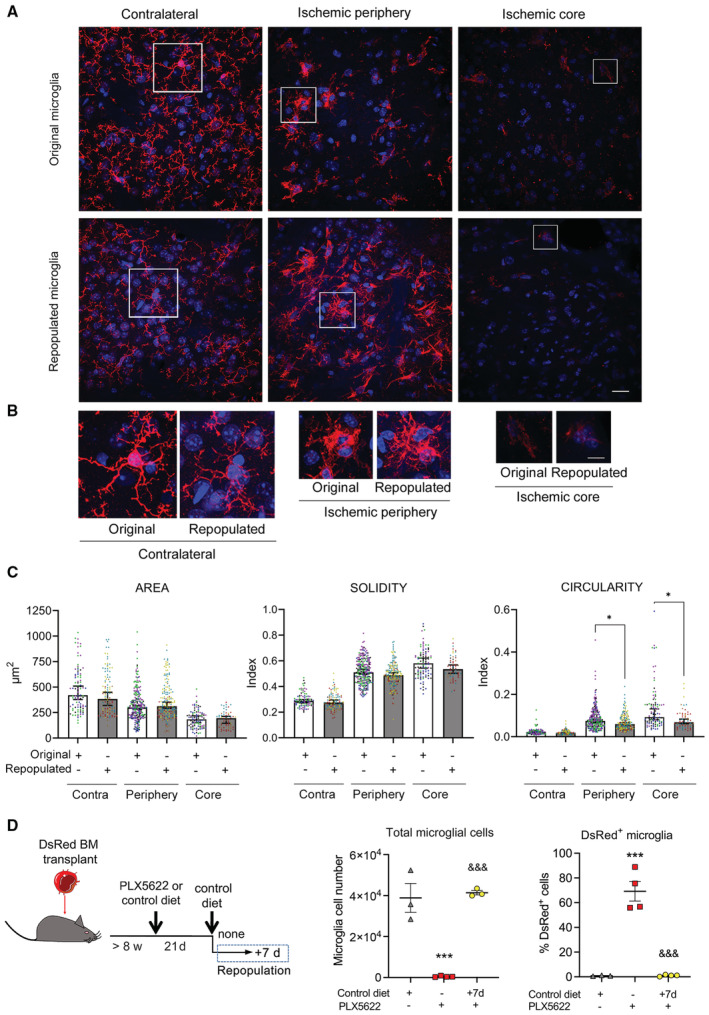
Microglia cells after depletion/repopulation. Related to Fig 4 A, B(A) Representative images of the striatum of young female mice with original microglia and repopulated microglia 4 days postischemia (*n* = 4 per group). Images show microglial cells immunostained with anti‐P2YR12 (red) in the noninjured contralateral hemisphere, the periphery of ischemia, and the lesion core. Cell nuclei are labeled with DAPI (blue). After repopulation, original and renewed microglia react to ischemia with changes in morphology. (B) Magnification of individual cells marked with squares in (D). Scale bar: (A) 20 μm, (B) 10 μm.CMorphometric analysis of microglial cells showed reduced area and increased solidity and circularity in microglia of the ischemic hemisphere versus the contralateral hemisphere in both groups. However, the increase in circularity at the periphery and core of infarction was attenuated in repopulated versus original microglia (*P = 0.047 in the periphery and *P = 0.033 in the core; Kruskal–Wallis test). Points show individual cells (in the periphery, core, and contralateral regions: *n* = 233, 96, and 91 cells for the control group, and *n* = 178, 58, and 109 cells for the renewed group, respectively), and colors indicate different mice (*n* = 4 mice per group). Bars show the median with 95% confidence interval.DRenewed microglia derive from brain cells. We generated chimeric mice by bone marrow transplantation from DsRed fluorescent reporter donor male mice to wild‐type recipient male mice (*n* = 11). After at least 8 weeks, mice were treated with PLX5622 in the diet (*n* = 8) or corresponding control diet (*n* = 3). Three weeks later, mice were euthanized (*n* = 4) or they were switched to control diet for repopulation for 7 days (*n* = 4 per group). The brain was studied via flow cytometry by measuring DsRed^+^ and DsRed^−^ cells in the gate of microglia cells. Absolute number of microglial cells was strongly reduced after microglia depletion (PLX5622 diet; one‐way ANOVA and Holm–Šídák's multiple comparisons test ***P = 0.0002 versus control diet). Microglia numbers recovered after 7 days of repopulation (^&&&^ *P* = 0.0002 versus depleted cells). The % of DsRed cells in the microglia gate of mice fed control diet was negligible. However, after microglia depletion (PLX5622 treatment; ANOVA and Holm–Šídák's, ***P < 0.0001 versus control diet) there was a high % of DsRed^+^ cells within the very small population of CD45^low^CD11b^+^ cells indicating the presence of a few CSF1R‐independent infiltrating cells. Importantly, the proportion of DsRed^+^ cells was negligible after mice were switched to control diet and the number of microglia increased (^&&&^ *P* < 0.0001 versus depleted mice). Values are expressed as the mean ± SEM.

We then hypothesized that microglia renewal by depletion/repopulation could modify some features of the microglial phenotype in old mice, thus improving stroke outcome. Microglia can be depleted via several strategies (Waisman *et al*, 2015; Miron & Priller, 2020). We used treatment with the CSF1R antagonist PLX5622 provided in the diet as it strongly reduces brain microglia content after 2–3 weeks of treatment, as previously reported (Elmore *et al*, 2018; Otxoa‐de‐Amezaga *et al*, 2019). Microglia repopulate the brain after interruption of the PLX5622 diet and switch to control chow (Elmore *et al*, 2018; Miron & Priller, 2020). Following PLX5622 diet, we switched to control diet for 7 days prior to ischemia and studied the brain 4 days postischemia. Microglia repopulated the contralateral and ipsilateral hemispheres. Renewed microglia showed ramified morphology similar to the original microglia, and the cells acquired reactive morphology at the periphery of infarction and in the lesion core that resembled the morphology displayed by the original microglia in these locations (Fig EV3). Morphometric analysis of striatal microglia showed no differences between original and renewed microglia in the contralateral hemisphere. In the ischemic hemisphere, microglia of both groups showed lower area and higher solidity and circularity at the periphery and core of infarction versus the contralateral hemisphere, as expected. However, the increase in circularity in the ischemic hemisphere was less pronounced in the repopulated microglia versus the original microglia (Fig EV3), suggesting that morphological changes in response to ischemia were slightly attenuated in the repopulated microglia. New repopulated microglia originate from brain‐resident cells rather than peripheral hematopoietic cells, as demonstrated in chimeric mice generated by transplanting bone marrow from DsRed mice to recipient wild‐type mice that were later subjected to microglia depletion with PLX5622 and repopulation for 7 days (Fig EV3), in agreement with previous findings (Huang *et al*, 2018).

**Figure EV4:**
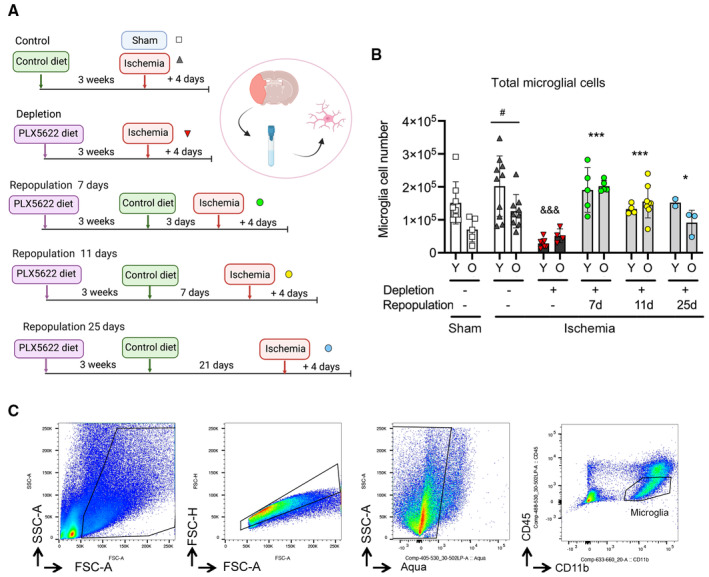
Effects of microglia repopulation. Related to Fig 4 AExperimental design for microglia depletion and repopulation in young (3–4 months; *n* = 39) and old (20–22 months; *n* = 34) female mice. We depleted microglia via a PLX5622 diet for 3 weeks. Mice were repopulated by switching to the corresponding control diet for 3, 7, or 21 days prior to ischemia induction. The brain was studied 4 days postischemia and during this period control diet was maintained. Total repopulation times: 7, 11, and 25 days, respectively. As controls, we used mice subjected to control diet and studied 4 days postischemia or sham operation.BThe number of microglia recovered postischemia was lower in old (O) than in young (Y) mice fed a control diet (two‐way ANOVA and Šídák's multiple comparisons test, #*P* = 0.0134). The PLX5622 diet strongly reduced the number of microglial cells postischemia in both age groups (^&&&^ *P* < 0.0001 versus ischemic mice on control diet); switching to control diet increased the number of microglia versus depleted mice (***P < 0.0001 at day 7, ***P = 0.0003 at day 11, and *P = 0.0377 at day 25; two‐way ANOVA and Dunnett's multiple comparison test). Ischemic mice: *n* = 10 Y and *n* = 9 O with control diet; *n* = 7 Y and *n* = 4 O with PLX5622 diet (depleted); *n* = 5 Y and *n* = 5 O with PLX5622 diet + 7 d (repopulated for 7 days); *n* = 4 Y and *n* = 8 O with PLX5622 diet + 11 d (repopulated for 11 days); *n* = 2 Y and *n* = 3 O with PLX5622 diet + 25 d (repopulated for 25 days). Sham mice: *n* = 8 Y and *n* = 5 O.CGating strategy for cell sorting to obtain the CD45^low^CD11b^+^ microglia shown in (B).

In other experimental groups, we allowed microglia repopulation for different time periods: 3, 7, or 21 days. We then induced brain ischemia and studied the mice 4 days later (Fig EV4). As expected, the PLX5622 diet reduced microglia cell number and the return to control diet increased microglia cell number, as seen in ischemic mice (Fig EV4). For the subsequent experiments, we chose to induce ischemia after 7 days of repopulation and study the mice 4 days post‐ischemia, i.e. 11 days repopulation in total, since at this time point microglia cell numbers had achieved values comparable to those of control mice and we could recover a substantial number of microglial cells for further study (Fig EV4). We also checked whether leukocyte infiltration 4 days postischemia differed between old mice with or without microglia repopulation; we did not detect differences between these groups (Appendix Fig S2).

**Figure 4:**
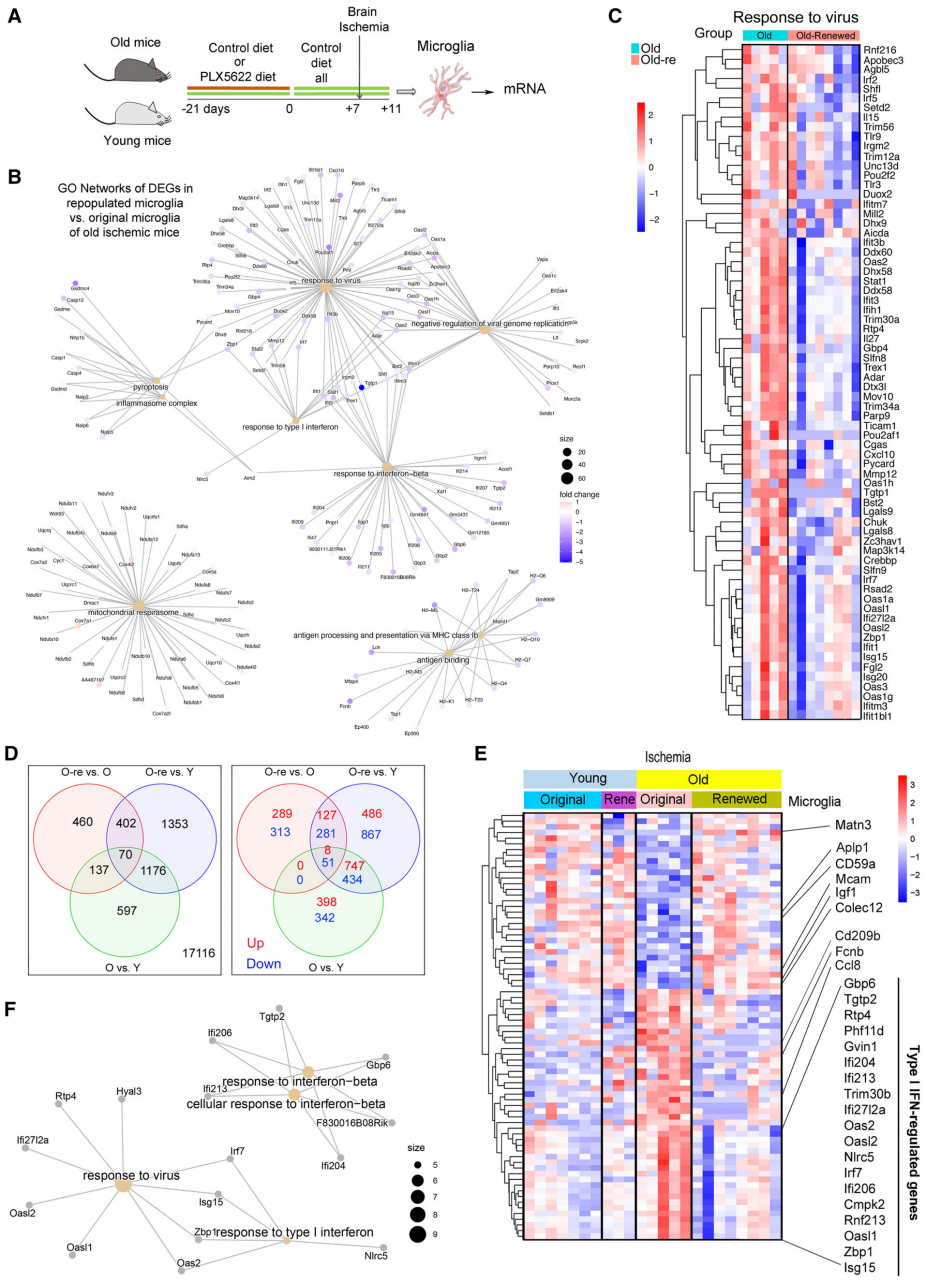
Microglia repopulation restores certain transcriptional features of old microglia after ischemia Young (3–4 months) and old (21–22 months) female mice were fed control diet (*n* = 12; *n* = 5 old mice and *n* = 7 young mice) or were treated with diet containing PLX5622 for 21 days to deplete microglia followed by restoration of control diet for 7 days to induce microglia repopulation (*n* = 11; *n* = 8 old mice and *n* = 3 young mice). Microglia were FACS sorted and RNA extracted for comparison of the transcriptomic profile of microglia obtained from old female ischemic mice with repopulated microglia (GSE196737). Created with BioRender.Cnetplot illustrating GO networks of DEGs in renewed microglia versus original microglia of old mice after brain ischemia.Heatmap showing reduced expression of genes related to the GO term “Response to virus” in renewed microglia.Venn diagram showing DEGs common and unique to the comparisons of the microglia obtained under the various experimental conditions after ischemia in female mice. There are 137 DEGs common for “microglia of old versus young ischemic mice” and “repopulated (re) versus original microglia of old ischemic mice” (upper diagram). Notably, none of these 137 genes is commonly upregulated or downregulated, signifying that they go in opposite directions in each comparison.Heatmap illustrating expression of 75 annotated genes of this group. Microglia renewal prevents the effect of old age by upregulating the expression of genes previously downregulated in old microglia and *vice versa*.Cnetplot illustrating the results of pathway analysis of the aforementioned 75 genes shows that the most enriched pathways correspond to IFN signaling.

**Figure EV5:**
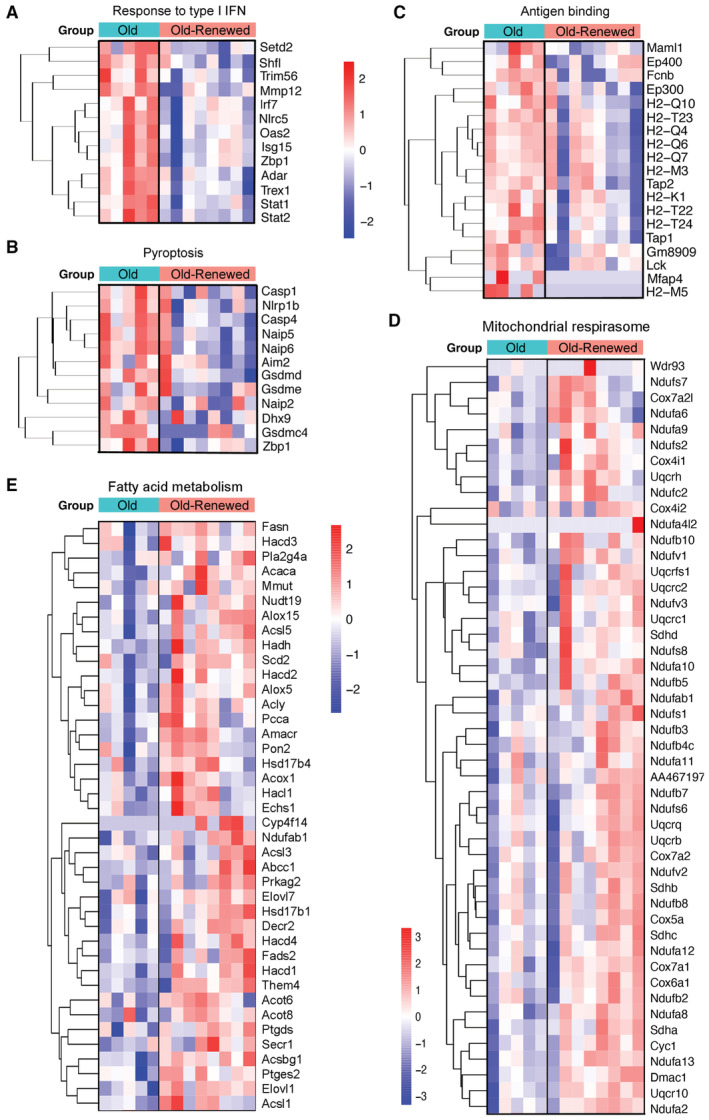
Enrichment of genes in GO pathways in renewed microglia of old ischemic mice versus the original microglia of old ischemic mice. Related to Fig 4 RNAseq analysis of microglia obtained by FACS from the brain of old mice with repopulated microglia (Old‐renewed; *n* = 8) versus old mice with the original microglia (*n* = 5) 4 days after ischemia.
A–EHeatmaps illustrate DEGs in various GO pathways downregulated (A–C) and upregulated (D, E) in renewed microglia of old mice after ischemia in relation to the original microglia of old ischemic mice. Downregulated GO terms include: (A) “Response to type I IFN,” (B) “Pyroptosis,” and (C) “Antigen binding.” By contrast, enriched pathways in renewed microglia include: (D) “Mitochondrial respirasome” and (E) “Fatty acid metabolism”.

The comparison of the transcriptomic profile of renewed microglia versus original microglia of old mice after ischemia (Fig 4A) detected 1,069 DEGs. Of these, 424 genes were upregulated and 645 genes were downregulated in renewed microglia. The downregulated GO terms (Dataset EV4) included innate immune responses like “Response to virus” (Fig 4B and C) and “Response to type I interferon” (Fig EV5). The GO terms “Pyroptosis” (Fig EV5) and “Inflammasome complex” were downregulated in repopulated microglia. Overall, the results showed that microglia repopulation in old mice reduced the innate immune and inflammatory responses displayed by these cells after ischemia. The antigen presentation capacity was also downregulated after microglia renewal, as illustrated by underrepresentation of the GO terms “Antigen binding,” “Antigen processing and presentation of endogenous antigen,” and “Antigen processing and presentation of endogenous peptide antigen via MHC class I” (e.g., Fig EV5). By contrast, renewed microglia of old ischemic mice displayed upregulation of pathways related to protein synthesis and metabolic activation (Dataset EV4). We illustrate this finding by showing the upregulated genes of the GO term “Mitochondrial respirasome” (Fig EV5). Microglia renewal also seemed to affect lipid metabolism (Fig EV5), as shown by upregulation of the GSEA pathways “Fatty acid metabolism” and “Metabolism of lipids” highlighted by increased expression of genes involved in metabolizing very long‐chain fatty acids and peroxisome β‐oxidation, cholesterol intracellular transport, and regulation of cholesterol biosynthetic route, amongst others. Overall, the transcriptomic profile of renewed microglia of old ischemic mice shows a reduction in innate immune responses together with an improvement of mitochondrial function and protein and lipid metabolism when compared with the original resident microglia of old ischemic mice.

For comparative purposes, we also evaluated whether microglia renewal affected the transcriptomic profile in young ischemic mice (Dataset EV5). In contrast to renewed microglia of old mice, renewed microglia of young mice did not downregulate the expression of innate immune response pathways, nor did it upregulate genes involved in mitochondrial respiration and lipid metabolism, whereas upregulation of genes involved in protein synthesis seemed to be common to renewed microglia regardless of the mouse age. Downregulated pathways in renewed microglia were mainly related to cell cycle, suggesting a tight cell cycle control at this stage of microglia repopulation.

### Microglia renewal in old mice prevents the exaggerated type I interferon innate immune response of old microglia to brain ischemia

We then examined the DEGs found when comparing the different groups of microglia of ischemic mice (age effect and microglia renewal effect in old mice; Fig 4D). We focused on the 137 DEGs common to “old versus young” (age effect) and “old‐renewed versus old” (microglia renewal effect in old mice), as illustrated in the Venn diagram (Fig 4D, left; Dataset EV6). Of note, none of these genes were commonly upregulated or downregulated in both comparisons (Fig 4D, right). Therefore, these genes had to be regulated in opposite directions. Indeed, this was the case, as illustrated by the 75 annotated genes in the heatmap (Fig 4E) showing either upregulation or downregulation of genes in microglia of old mice versus microglia of young mice, and the restoration of gene expression by microglia renewal in old mice to the level of microglia of young mice. Amongst the genes downregulated in old microglia and upregulated after microglia renewal in old mice we found insulin growth factor 1 (*Igf1*), encoding a protein secreted by microglia with important synaptic and neurogenic functions (Schmidt *et al*, 2021). A pattern of gene expression similar to that of *Igf1* was observed for amyloid precursor‐like protein 1 (*Aplp1*), matrix protein Matrylin‐3 (*Matn3*), CD59a, Collectin‐12 (*Colec12*), and Melanoma Cell Adhesion Molecule (*Mcam*), which is a co‐receptor for VEGFR2. Within the genes upregulated in old microglia and restored by microglia renewal, the genes regulated by type I IFN stood out, e.g., *Isg15*, *Zbp1*, *Rnf213*, *Ifi206*, *and Irf7*, to mention but a few genes in this pathway. Other innate immune genes also downregulated by microglia renewal in old mice include the chemokine *Ccl8* and the pattern recognition receptors *Cd209b* and *Fcnb*. We also identified the restoration of expression levels of genes involved in lipid homeostasis and endoplasmic reticulum stress. Pathway analysis of these DEGs mainly detected type I IFN antiviral innate immune responses (Fig 4F, Dataset EV7). Overall, these analyses reinforced the finding that repopulated microglia show less prominent innate immune responses compared with original microglia of ischemic old mice and suggest improvement of the cellular metabolic profile, particularly affecting lipid metabolism.

### Microglia renewal strongly reduces the age‐dependent subset of microglia containing lipid droplets

**Figure 5:**
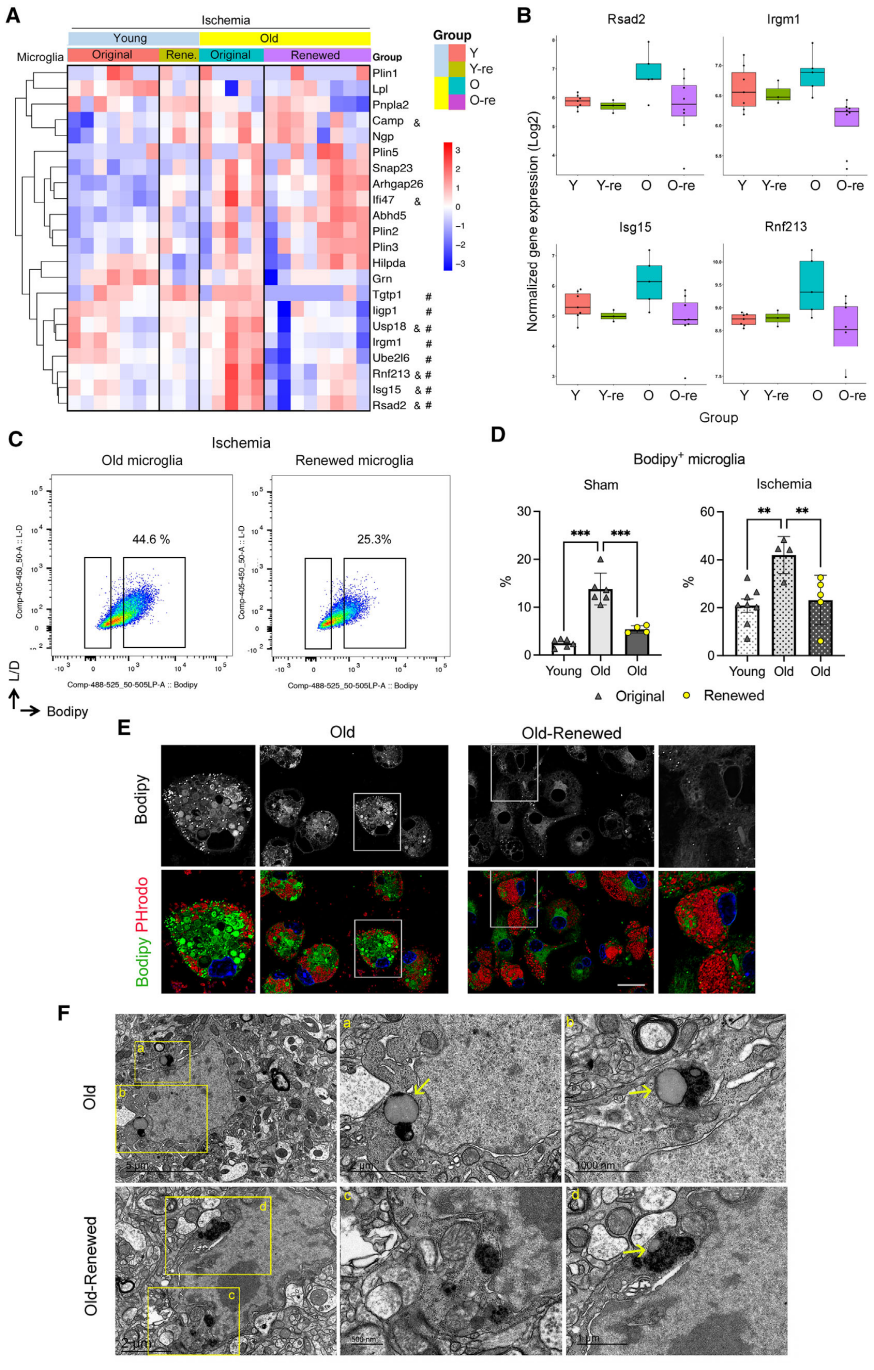
Microglia renewal eliminates the age‐dependent microglia containing lipid droplets Heatmap of lipid droplet‐associated proteins in microglia of young (*n* = 7) and old (*n* = 5) female mice and corresponding groups with microglia renewal (Re) under ischemia (*n* = 3 and *n* = 8). Old microglia show increased expression of some genes versus young microglia (^&^ *P* < 0.05). Microglia renewal in old mice prevents the age‐dependent increase in type I IFN genes (^#^ *P* < 0.05).Box plots depict normalized gene expression of representative genes encoding lipid droplet‐associated proteins of the IFN pathway. The graph shows boxes from the 25^th^ to 75^th^ percentiles, the median line, and whiskers down to the minimum and up to the maximum value, showing all points.Representative flow cytometry plots showing Bodipy^+^ CD11b^+^CD45^low^ microglia from old male mice with or without microglia renewal. Data show results from the ischemic brain hemisphere.Quantification of flow cytometry shows that microglia renewal reduced the percentage of Bodipy^+^ microglia in old mice in sham (***P = 0.0001) and ischemic (*P = 0.0057) conditions (one‐way ANOVA and Holm–Šídák's multiple comparisons test). For illustrative purposes, the values of young mice are also shown (original old versus young: ***P < 0.001 in the sham group and **P = 0.0026 in the ischemic group). Values show individual mice and bars show the mean ± SD.Primary microglia cultures were obtained using CD11b^+^ magnetic beads from old male mice with or without microglia renewal (*n* = 2 per group). Cells were exposed to red fluorescent pHrodo *Escherichia coli* bioparticles and were stained with green fluorescent Bodipy and studied by confocal microscopy. Black and white images illustrate raw intensity data of Bodipy staining. The lower images are corresponding merged channels showing Bodipy in green, phagocytosed bioparticles in red, and the DAPI^+^ nuclei in blue. Repopulated microglia show less lipid droplets and the cytoplasm is replenished with phagocytosed bioparticles. The square regions of the images indicate areas magnified at the side of each image. Scale bar = 20 μm.Electron microscopy images representative of microglia of old male mice with original microglia (Old) and with renewed microglia (Old‐Renewed; *n* = 3 per group). Images labeled (a–d) are higher magnifications of the areas marked with yellow squares in the images on the left. Lipid droplets are marked with arrows. Large lipid droplets are seen in microglia of old mice, whereas renewed microglia typically show no or small lipid droplets.

Microglia of old ischemic mice upregulated the expression of some of the genes encoding lipid droplet‐associated molecules even more than microglia of young ischemic mice (Fig 5A). Importantly, microglia renewal in old mice significantly reduced the expression of type I IFN genes encoding for molecules associated with lipid droplets, e.g., *Rsad2*, *Isg15*, *Rnf213*, and *Irgm1* (Fig 5B). Moreover, compared with untreated old mice, old mice with renewed microglia showed a reduced % of Bodipy^+^ microglia both under sham and ischemic conditions, approaching levels like those observed in corresponding microglia of young mice, as assessed by flow cytometry (Fig 5C and D). We then investigated whether microglia isolated from the brain of old mice and cultured for 7 days maintained the low Bodipy^+^ phenotype and their phagocytic capacity using pHrodo *E. coli* bioparticles as before. Microglia with Bodipy^+^ lipid droplets and scarce phagocytosed bioparticles were hardly observed in microglia cultures obtained from repopulated old mice compared with cultures obtained in parallel from untreated old mice (Fig 5E). Electron microscopy of the brain tissue of old mice showed microglia with typically large lipid droplets that were absent or showed a small size in renewed microglia of old mice (Fig 5F). These experiments support the concept that lipid droplet enrichment reduces the cell's capacity to accumulate phagocytosed materials, suggesting that microglia repopulation in old mice improves microglia function.

### Microglia renewal improves the neurological outcome after stroke

**Figure 6:**
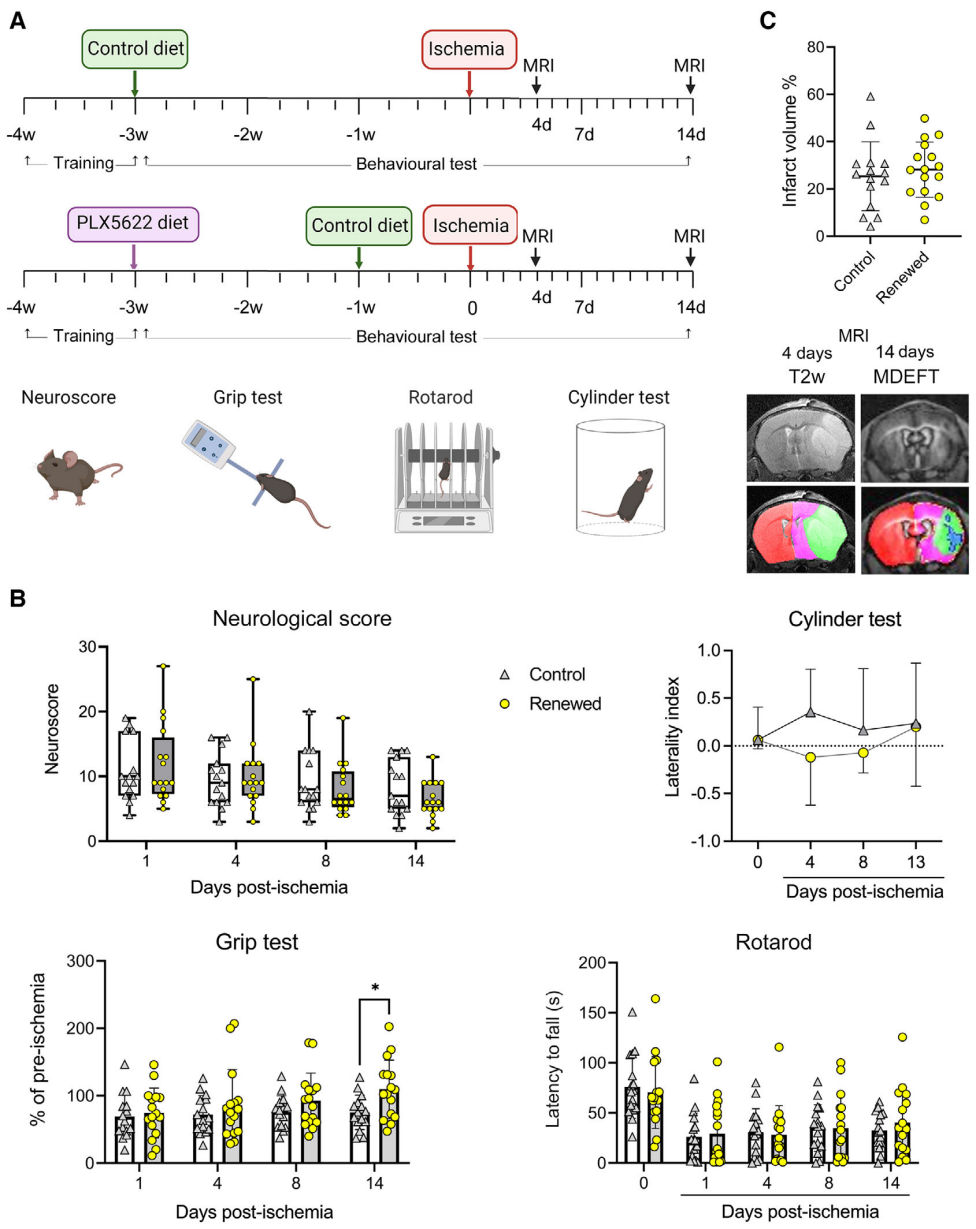
Microglia repopulation ameliorates the neurological outcome of stroke Experimental design for microglia depletion and repopulation in old (21–22 month) female mice. We depleted microglia by providing a diet containing the CSF1R antagonist PLX5622 for 3 weeks. Microglia repopulation was induced by switching to control diet for 7 days prior to induction of ischemia (*n* = 17). Treatment controls were fed a corresponding control diet (*n* = 16). Behavioral testing (including training sessions) was conducted throughout the experiment and mice underwent two MRI studies. Measures were obtained the day prior to ischemia (basal value, time = 0) and at different time points postischemia until day 14. Image created with BioRender.The neuroscore value (mean ± SD) decreased from day 1 to day 14 by 23% in the control group (from 10.8 ± 4.7 to 8.3 ± 4.2; *n* = 15) and by 46% in the microglia renewed group (from 11.9 ± 6.1 to 6.4 ± 2.8; *n* = 16). The variability within groups was large and differences between groups were not statistically significant (*P* = 0.21, nonparametric Kruskal–Wallis test with a repeated‐measures design). Points are individual values for each mouse, box and whiskers show the median, and bars indicate min. and max. values. One animal of each group died after ischemia and these mice were excluded. The grip test assessing limb strength showed a better performance in the group with repopulated microglia (Two‐way ANOVA and Šídák's multiple comparisons test, *P = 0.047). Values are expressed as % of the corresponding basal value (preischemia) for each mouse. Rotarod test did not show differences between groups. Values are expressed as latency to fall (seconds) from the running wheel at different time points postischemia (higher means better). Laterality was assessed via the cylinder test in a subgroup of mice (*n* = 7 renewed, *n* = 6 control). Positive values in the laterality index (ranging from −1 to +1) indicate impairment of the affected hindlimb, i.e., contralateral to the injured brain hemisphere. There were no differences between groups with the Mann–Whitney test. However, data analysis with the Wilcoxon signed‐rank test showed that the repopulated group was significantly different (*P = 0.016) from a theoretical median of +1 at day 4 postischemia whereas the control group was not (*P* = 0.062). The results indicate some transient laterality effect in the control group at day 4 postischemia that was not detected in the repopulated group. Bars represent mean ± SD.The MRI lesion volume, as illustrated in the corresponding false color images below, showed no differences in infarct volume in mice with (*n* = 16) or without (*n* = 15) microglia renewal. Results correspond to day four postischemia. Representative MRI images were obtained on day four postischemia using T2w MRI and day 14 using a Modified Driven Equilibrium Fourier Transform (MDEFT) MRI sequence (7.0 T BioSpec 70/30, Bruker BioSpin). Values show data for individual mice and/or the mean ± SD.

To investigate whether renewal of microglia in old mice affected the neurological dysfunction induced by stroke we conducted several behavioral tests in a longitudinal manner up to 14 days postischemia in mice treated with control diet or subjected to microglia depletion and repopulation (Fig 6A). The mean neuroscore value decreased (improved) from day 1 to day 14 by 23% in the control group and by 46% in the microglia renewed group. However, the variability within groups was high and differences between groups were not statistically significant (Fig 6B). Old mice with renewed microglia showed improvement of the forelimb strength at day 14, as assessed by the grip test (Fig 6B). The latency to fall in rotarod, which assesses motor activity, was higher (indicating better performance) in the repopulated group, but group differences were not statistically significant. The laterality index, as assessed with the cylinder test, showed a trend toward positive laterality, indicating impairment of the affected limb, in the control group at day 4 postischemia but not in the repopulated group (Fig 6B). The volume of the brain lesion, as assessed by MRI at day 4 postischemia was similar in both groups (Fig 6C), like the mean body weight before ischemia and 1 day later (Appendix Fig S3). At day 2, mean body weight dropped more in the control group, but differences were not statistically significant (Appendix Fig S3). Of note, we did not detect behavioral differences between diet groups during treatment prior to ischemia in old mice (Appendix Fig S3). Overall, microglia renewal in old mice prior to ischemia did not reduce the size of the lesion, nor did it improve the neurological score in the first few days postischemia. However, old mice with renewed microglia showed signs of improvement of the neurological function 2 weeks after stroke suggesting that renewed microglia facilitated the recovery of function in old mice.

## Discussion

Worse stroke outcome in elderly people is attributed to increased vulnerability of the aging brain to ischemia, but knowledge of the underlying biology is still poor (Chen *et al*, 2010). Here we show that dysfunctional microglia contribute to the worse outcome of ischemic stroke in old mice. The underlying mechanisms involve an exaggerated type I IFN signature associated with age‐dependent lipid droplet accumulation in microglial cells. Renewing the microglia population of old mice increases the overall fitness of these cells by reducing the proportion of microglia containing lipid droplets. After stroke, repopulated microglia in old mice display attenuated innate immune responses and reduced lipid droplets compared with the original microglia of old mice. Importantly, old mice with repopulated microglia show improvement in the motor dysfunction caused by stroke.

Microglia derived from erythromyeloid progenitor cells of the yolk sac colonize the mouse brain at embryonic stages (Ginhoux *et al*, 2010; Schulz *et al*, 2012). These cells remain in the adult brain with negligible replacement by peripheral cells, and a low rate of self‐renewal under steady state. In humans, microglial cells can live for more than 20 years (Réu *et al*, 2017). Overall, the microglia lifespan is long, and cells display specific phenotypes in the aged brain (Norden *et al*, 2015; Hammond *et al*, 2019), which is characterized by the presence of dysfunctional microglia (Sierra *et al*, 2007; Streit *et al*, 2014; Safaiyan *et al*, 2016; Marschallinger *et al*, 2020). A hallmark of aging is the accumulation of lipofuscin inclusions (Mrak *et al*, 1997; Katz & Robison Jr, 2002), which are found in several cells including microglia (Streit *et al*, 2014; Safaiyan *et al*, 2016). These lipofuscin pigment deposits are autofluorescent intracellular bodies containing lipid residues derived from the oxidation of polyunsaturated fatty acids and proteins (Jolly *et al*, 2002). Although there are different kinds of lipofuscin depending on the cell type, tissue, and disease condition, they often correspond to still‐active lysosomes containing hydrolytic enzymes. A previous study found that microglia repopulation in old mice restores the age‐dependent dysfunction in lysosomal structure and intracellular lipofuscin accumulation (O'Neil *et al*, 2018). Importantly, the increased myelin degradation by microglia in the aged brain is a recognized trigger of lipofuscin‐like lysosomal inclusions (Safaiyan *et al*, 2016). Lysosomes carry out lipid hydrolysis and recruit lipid droplets to generate free fatty acids. We observed lysosomes attached to lipid droplets in microglia after ischemia, and similar observations were reported in microglia of old rats (Vaughan & Peters, 1974). These findings suggest cellular lipid recycling in microglia through lipophagy, i.e., a form of autophagy, both after brain ischemia and in aged subjects. Moreover, the transcriptomic profile of old versus young microglia after ischemia showed enrichment of metabolic pathways related to lipid homeostasis, whereas pathways involved in peroxisomal beta‐oxidation of very long‐chain fatty acids were downregulated. Altogether, these findings are compatible with age‐dependent impairment of the capacity to shorten the chain of very long‐chain fatty acids derived from lysosomal lipid degradation (Wanders *et al*, 2020), thus suggesting alterations in lipid disposal in microglia of aged subjects. In turn, we found that repopulated microglia in old mice after stroke show transcriptional enrichment of pathways related to lipid metabolism and have less lipid droplet accumulation. Additional features of microglia of old mice that were restored after microglia repopulation could contribute to improve neurological recovery after stroke. For instance, relative to young mice, microglia of old mice showed downregulation of *Igf1* mRNA after ischemia, whereas microglia renewal in old mice upregulated the expression of *Igf1*. IGF1 plays important trophic and neurogenic functions in the CNS (Schmidt *et al*, 2021). Moreover, the administration of IGF1 has shown beneficial effects in experimental stroke models (Lioutas *et al*, 2015). Therefore, microglia renewal in old mice is likely a critical player in the observed benefits of PLX5622 treatment for stroke outcomes. Although we did not detect changes in leukocyte infiltration in the repopulated tissue of ischemic mice, we cannot fully exclude that the effects of treatment on peripheral immune cells (Lei *et al*, 2020) also contributed to stroke recovery. Altogether, the current findings in repopulated microglia demonstrate attenuation of certain metabolic features typically associated with microglia of old mice.

Lipid droplets originate from the endoplasmic reticulum and store neutral lipids that can be later metabolized via β‐oxidation to generate ATP. After bacterial infection, hepatocytes generate lipid droplets surrounded by antimicrobial proteins (Bosch *et al*, 2020). Lipid droplets are critically involved in mounting an efficient IFN response against different types of infection (Knight *et al*, 2018; Monson *et al*, 2018, 2021; Bosch *et al*, 2020; Truong *et al*, 2020). Our results show lipid droplet biogenesis associated with the innate immune response induced by brain ischemia in microglia. Of note, aging and ischemia upregulated Viperin, which can promote TLR7‐ and TLR9‐mediated production of type I IFN in plasmacytoid dendritic cells (Saitoh *et al*, 2011). Aged microglial cells rich in lipid droplets showed less phagocytosed *E. coli* bioparticles supporting the reported impaired phagocytic function of microglia containing lipid droplets (Marschallinger *et al*, 2020). The accumulation of lipid droplets seems to contribute to microglia dysfunction in old mice, impairing the cellular fitness for tissue clearance. Very recent studies reported ischemia‐induced changes in lipid metabolism in microglia (Beuker *et al*, 2022), and accumulation of lipid droplets in chronic stroke lesions impairing stroke recovery (Becktel *et al*, 2022).

This study also demonstrates that brain ischemia induces lipid droplet biogenesis in microglia of young mice within hours/days after stroke onset. Acute lipid droplet biogenesis could be advantageous since it protects from lipotoxicity caused by free fatty acids and is beneficial in other conditions like infection (Knight *et al*, 2018; Bosch *et al*, 2020). Our results show that microglia accumulate lipid droplets shortly after stroke onset due to lipid enrichment plausibly attributable to the innate immune response, phagocytosis of damaged tissue, and immunometabolic disturbances that are associated with the increased expression of certain lipid droplet‐associated proteins, such as Plin2. Brain ischemia causes tissue necrosis and inflammation, increasing the requirement of phagocytosis to remove dead cells, tissue debris, myelin fragments, and infiltrating leukocytes (Otxoa‐de‐Amezaga *et al*, 2019). During this process, we show that microglia develop a strong type I IFN signaling response likely attributable to recognition of intracellular danger signals, e.g., via the cGAS/Sting pathway (Li *et al*, 2020; Liao *et al*, 2020), thus upregulating the expression of innate immune molecules associated to lipid droplets. Moreover, we found that IFN signaling is involved in ischemia‐induced lipid droplet biogenesis since it is abrogated in Stat1 knockout mice defective in IFN signal transduction. The postischemic IFN response is further exacerbated in microglia of old versus young mice, whereas it is reduced in repopulated microglia of old mice to the levels of microglia of young mice, in consonance with the reduction in age‐associated lipid droplets. While acute lipid droplet biogenesis in microglia after ischemia resembles the homeostatic response to immunometabolic challenges (Bosch *et al*, 2020; Monson *et al*, 2021), chronic accumulation of lipids and lipofuscins in microglia of old mice is related to aging and can impair microglia function (Sierra *et al*, 2007; Cantuti‐Castelvetri *et al*, 2018; Marschallinger *et al*, 2020; Safaiyan *et al*, 2021). Microglia renewal in old mice reduced age‐associated lipid droplets and favored motor function recovery after stroke.

We do not know whether all microglial cells are able to generate lipid droplets under specific conditions and putative differences in lipid droplet composition between young and old individuals, as well as between male and female since estrogen affects lipid metabolism (Morselli *et al*, 2018) and microglial activity (Vegeto *et al*, 2003). Microglia heterogeneity has been described depending on age, brain region, and injury/disease (Hammond *et al*, 2019; Li *et al*, 2019; Masuda *et al*, 2019; O'Koren *et al*, 2019). Therefore, it is possible that the regional location contributed to the abundance of Bodipy^+^ microglia since, for instance, aging‐related lipofuscin accumulation associated with myelin degradation is more prominent in the white matter than in the gray matter (Mrak *et al*, 1997). Strategies designed to remove age‐related dysfunctional microglia or to improve microglia lipid disposal in the elderly could ameliorate microglia function, increasing the cellular resilience to cope with brain injury induced by stroke and possibly other neurological conditions.

## Materials and Methods

### Materials and correspondence

Further information and requests for resources and reagents should be directed to and will be fulfilled by the corresponding authors Mattia Gallizioli or Anna M. Planas.

### Mice

We used adult young (3–4 months) mice and old (21–22 months) mice in the C57BL/6 background obtained from Janvier (Lyon, France) or Envigo (Amsterdam, The Netherlands). We also used CX3CR1creERT2 mice (B6.129P2(C)‐Cx3cr1tm2.1(cre/ERT2)Jung/J, #SN020940) crossed with reporter ROSA26 tdTomato mice (B6.Cg‐Gt(ROSA)26Sortm9(CAG‐tdTomato)Hze/J, #SN007909) obtained from The Jackson Laboratory. The latter mice received tamoxifen 3 weeks before surgery. For generating chimeric mice, we used donor DsRed fluorescent reporter mice from our colony. Stat1^−/−^ mice (129S6/SvEv‐Stat1^tm1Rds^) and corresponding Stat1^+/+^ mice in the 129S6 background were initially obtained from Taconic. Mice were maintained at the animal house of the School of Medicine or Faculty of Psychology of the University of Barcelona (UB) under standardized environmental conditions, a 12 h dark/light cycle, and *ad libitum* access to food and water. We used female and male mice, but we did not mix sexes in all the experiments. For clarity, the sex of the mice is reported in the figure legends. Animal work was conducted following the Catalan and Spanish laws (Real Decreto 53/2013) and the European Directives. All experiments were conducted with the approval of the ethical committee (Comité Ètic d'Experimentació Animal, CEEA) of UB, and the local regulatory bodies of the Generalitat de Catalunya, and in compliance with the NIH Guide for the Care and Use of Laboratory Animals. The work is reported following the ARRIVE guidelines.

### Human brain tissue

We obtained the brain tissue of nine patients who died at 1, 3, 4, 5, 6, 18, or 140 days after stroke onset at the Stroke Unit of the Hospital Clinic of Barcelona. Written consent was obtained from the families for tissue removal after death for diagnostic and research purposes at the Neurological Tissue Bank of the Biobank‐Hospital Clinic‐Institut d'Investigacions Biomèdiques August Pi i Sunyer (IDIBAPS). The study had the approval of the Ethics Committee of this Hospital (Reg. HCB/2020/1455) and conformed to the principles set out in the WMA Declaration of Helsinki and the Department of Health and Human Services Belmont Report. The basic characteristics of the patients are provided in Table EV1.

### Induction of brain ischemia

Cerebral ischemia was induced in mice by transient occlusion of the right middle cerebral artery (MCAo) with the intraluminal technique, as described (Gallizioli *et al*, 2020), with modifications. In brief, anesthesia was induced with 4% isoflurane in a mixture of 30% O_2_ and 70% N_2_O, and it was maintained with 1–1.5% isoflurane in the same mixture using a facial mask. With the animal in supine position on a heated surgery table (37°C), a longitudinal cut was produced in the ventral midline of the neck and the submaxillary glands, and the omohyoid and sternothyroid muscles were separated, exposing the right carotid artery. A monofilament (#701912PK5Re, Doccol Corporation, Sharon, MA) was introduced through the right external carotid artery up to the level where the MCA branches out. We allowed the mice to wake up and kept them on a heated blanket at 37°C. After 45 min of arterial occlusion, mice were anesthetized again, the filament was cautiously removed, and the suture of the ipsilateral CCA was removed to allow reperfusion. CBF was monitored with laser Doppler flowmetry (Perimed, AB, Järfälla, Sweden) during the procedure to guide the filament insertion and monitor perfusion. Mice received analgesia (buprenorphine, 150 μl, 0.015 mg/ml, via s.c.) and were kept on a thermal blanket at 37°C for 1 h after surgery. Pre‐established exclusion criteria were either unsuccessful MCAo resulting in no infarction or technical surgical complications. Sham‐operated mice received the same surgical interventions, anesthesia, and analgesia as the ischemic mice but the MCA was not occluded in the former. At different time points, we euthanized the mice and obtained the brain, which received a code that did not reveal the identity of the experimental group.

### Drug treatments

For microglia depletion, mice received the CSF1R inhibitor PLX5622 (supplied by Plexxikon Inc, Berkeley USA; or purchased from MedChemExpress. #HY‐114153) following a previously reported protocol (Otxoa‐de‐Amezaga *et al*, 2019). PLX5622 was mixed into AIN‐76A rodent diet at 1,200 ppm (Research Diets, NJ, USA; supplied by Brogaarden, Denmark). Treatment controls received AIN‐76A drug‐free standard chow for the same period. Both diets were given in parallel in groups of five animals per cage. Mice received the diet *ad libitum*. In as much as possible, we blinded the treatment, e.g., for evaluation of behavioral data. The various dosing regimens are explained in the figures.

### Generation of chimeric mice

We generated chimeric mice by chemical ablation of the bone marrow of wild‐type (WT) recipient mice followed by transplantation of bone marrow from DsRed reporter donor mice, as described (Otxoa‐de‐Amezaga *et al*, 2019). In brief, adult (2‐month‐old) male WT mice received three intraperitoneal injections of the chemotherapeutic agent busulfan (30 μg/g body weight) 7, 5, and 3 days prior to the transfer of five million bone marrow cells from DsRed donor mice via the tail vein. The mice were studied at least 8 weeks after transplantation.

### Assessment of neurological function

We used a composite neuroscore modified from a previously reported neuroscore (Orsini *et al*, 2012). The score ranged from 0 (no deficits) to 37 (poorest performance in all items) and it was calculated as the sum of general and focal deficits, including the following general deficits (scores): hair (0–2), ears (0–2), eyes (0–3), posture (0–3), spontaneous activity (0–3), and epileptic behavior (0–1); and the following focal deficits: body symmetry (0–2), gait (0–4), climbing (0–3), circling behavior (0–3), forelimb symmetry (0–4), compulsory circling (0–3), and whisker response (0–4). For behavioral tests, mice received preoperative training prior to starting drug treatments and were tested during treatments to check for possible behavioral alterations. Tests were performed at different time points prior and after ischemia, and the last test performed prior to ischemia was taken as the basal value. The rotarod test (RotaRod/RS Panlab Harvard Apparatus) assesses motor activity. We carried out three trials on an accelerating rod, starting at 4 rpm with an increasing acceleration of 1 rpm each 8 s with 15 min of rest between the trials. The latency before failing was taken as the average of the three trials. The grip test measures the maximal muscle strength of forelimbs when the mouse loses its grip on the T‐bar. A computerized grip strength meter (Alemo 2450, Ahlborn) was used. We performed three measures on each testing day, and we used the best measure. The laterality index was calculated with the cylinder test. In brief, the mouse was introduced in a plexiglass cylinder with an internal diameter of 9.5 cm and a height of 15 cm, and the mouse was video‐recorded for 10 min. We counted in the videos the number of times that the mouse raised the body and touched the cylinder (first touch only) for vertical exploration of the cylinder. We counted the number of first contacts with the right (nonaffected) or left (affected) forelimb, or both simultaneously. The laterality index was calculated as the number of contacts with the right limb minus those with the left limb, divided by the total number of first touches. Two independent observers analyzed the videos, and the mean value was calculated.

### Magnetic resonance imaging (MRI)

The brain lesion was imaged using MRI in a 7.0 T BioSpec 70/30 horizontal animal scanner equipped with a 12‐cm inner diameter actively shielded gradient system (400 mT/m). The receiver coil was a phased array surface coil for mouse brain (Bruker BioSpin, Ettlingen, Germany). Four days and 14 days after induction of ischemia, the brain was studied with T2w turbo RARE fast spin‐echo MRI sequences with one effective echo time (ET) = 33 ms, slice thickness = 0.5 mm, repetition time (TR) = 2,336 ms, field of view = 20 × 20 mm^2^, matrix size = 256 pixels, and in‐plane spatial resolution = 0.078 mm. For evaluation of structural alterations that were no longer observable in T2 relaxometry maps at day 14 postischemia, we acquired high‐resolution 3D Modified Driven Equilibrium Fourier Transform (MDEFT) images. The scan parameters were as follows: TE = 2.2 ms, slice thickness = 0.5 mm, TR = 4,000 ms, four segments, in‐plane spatial resolution 0.078 mm. Images were obtained with ParaVision 6.0 software (Bruker BioSpin, Ettlingen, Germany). Image analysis was carried out using ImageJ.

### Brain tissue processing and flow cytometry

Mice were anesthetized with isoflurane and euthanized by cervical dislocation. The brain was carefully collected, in order not to damage the tissue, and immersed in Hanks' Balanced Salt solution w/o ions (HBSS w/o Ca^2+^ and Mg^2+^; #14175‐053, Thermo Fisher Scientific) on ice. The forebrain was dissected with a scalpel, discarding the cerebellum and the olfactory bulbs, and minced into small pieces. The Neural Tissue Dissociation Kit – P (NTDK, #130‐092‐628, Miltenyi Biotec) based on the enzymatic dissociation with papain was used to homogenize the tissue. Mechanical dissociation was performed with the gentleMACS™ Octo Dissociator (#130‐096‐427, Miltenyi Biotec: 1× m_Brain_1 program and 1× ABDK_37C program by Miltenyi) with the tissue immersed in the buffers and enzymes from the NTDK kit, according to manufacturer instructions. The tissue was then filtered through a 70 μm cell strainer (#352350, Falcon) previously humidified with HBSS with Ca^2+^ and Mg^2+^ (#14025‐092, Thermo Fisher Scientific). Then, cells were separated from myelin by an immunomagnetic separation method. Brain cells were incubated with Myelin Removal Beads II (#130‐096‐733, Miltenyi Biotec) and then passed through LS Columns (#130‐042‐401, Miltenyi Biotec) held onto the OctoMACS Separator (#130‐091‐051, Miltenyi Biotec) and to the MACS® MultiStand (#130‐042‐303, Miltenyi Biotec). Unspecific binding of antibodies was blocked by previous incubation for 10 min with anti‐CD16/CD32 (Fc block, clone 2.4G2; #55314, BD Pharmingen) in Fluorescence‐Activated Cell Sorting (FACS) buffer at 4°C. Live/dead Aqua cell stain (#L34957, Thermo Fisher Scientific) was used to determine the viability of cells. Cells were incubated during 30 min at 4°C with the following primary antibodies: CD11b (clone M1/70, AF647, #557686, BD Pharmingen), CD45 (clone 30‐F11, Brilliant Violet 786, #564225, BD Horizon). After washing once with phosphate‐buffered saline (PBS), cells were stained with BODIPY (4,4‐difluoro‐3a,4adiaza‐s‐indacene, Molecular Probes BODIPY 493/503, #D3922, Life Technologies) diluted 1:1,000 during 15 min at RT. After a wash with cold FACS buffer, the cells were analyzed in a BD FACSAriaII cytometer using FacsDiva software (version 5, BD Biosciences, San Jose, CA, USA). Data analyses were performed with FlowJo software (version 10, FlowJo LLC, Ashland, OR, USA).

### Cell isolation

Microglia for the ischemic versus control (data in Fig 1) were isolated from the brain of CX3CR1cre^ERT2^‐ROSA26 tdTomato (tdT) mice via FACS after intracardiac perfusion with 2 ml of cold PBS. Brains were collected in cold HBSS buffer (w/o Ca^2+^ and Mg^2+^; #14175‐053, Thermo Fisher Scientific). The brain tissue was enzymatically dissociated using the Neural Tissue Dissociation Kit (P) (#130‐092‐628, Miltenyi Biotec). The gentleMACS™ Dissociator (#130‐096‐427, Miltenyi Biotec) was used for mechanical dissociation steps following the Neural Tissue Dissociation Kit (P) manufacturer protocol for dissociation without heaters. The digested tissue was filtered twice with 70 μm and 40 μm filters, washing with cold Hanks' balanced salt solution (HBSS; with Ca^2+^ and Mg^2+^; #14025‐092, Thermo Fisher Scientific). Cells were separated from myelin and debris by 30% isotonic percoll gradient (#17‐0891‐01, GE Healthcare) in Myelin Gradient Buffer (MGB). Samples were centrifuged at 950 × *g* without acceleration or brake, for 30 min at 4°C. Cells were collected from the bottom of the tube, washed once with cold FACS Stain Buffer (#554656, BD Biosciences), and processed for FACS in a FACSAriaII sorter (BD Biosciences). No staining was required since the expression of tdT was used to separate microglial cells.

Microglia for the old versus young comparisons were isolated from the brain of WT mice after the assigned depletion/renewal protocol and ischemia. Mice were euthanized under deep anesthesia and the brain was processed in the same way as for the flow cytometry experiments to obtain a single‐cell suspension (see above). Unspecific binding was blocked by incubation for 10 min with anti CD16/CD32 (in FACS buffer at 4°C). Live/dead Aqua cell stain was used to determine the viability of cells. Cells were incubated with the following primary antibodies during 30 min at 4°C: CD11b (clone M1/70, APC‐Cy7, #557657, BD Pharmingen), CD45 (clone 30‐F11, FITC, #553080, BD Pharmingen). When sorting included Bodipy staining, as in flow cytometry, we used Viakrome 405 Fixable Viability Dye (#C36614, Beckman Coulter) instead of Aqua. After washing with FACS Stain Buffer (#554656, BD Biosciences), the cells were sorted in a FACSAriaII SORP sorter (BD Biosciences). Microglial cells were collected in sterile DPBS (#14190‐094, Thermo Fisher Scientific), centrifuged, and resuspended in lysis buffer (from PureLink™ RNA Micro Kit #12183016, Invitrogen) supplemented with 10% β‐mercaptoethanol and finally snap‐frozen in dry ice.

### Adult microglia culture

Microglia cells from adult young or old mice were isolated and cultured using immunomagnetic separation (Miltenyi Biotec, Germany). Mice were perfused via the left ventricle with 60 ml of cold saline and collected in HBSS buffer without calcium/magnesium (#14175‐05; Life Technologies). The brain tissue was enzymatically dissociated using the Neural Tissue Dissociation Kit‐P (as above). The gentleMACS™ Dissociator with Heaters (#130‐096‐427; Miltenyi Biotec) was used for mechanical dissociation steps during 30 min at 37°C. The digested tissue was filtered (70 μm) with HBSS buffer with calcium and magnesium and prepared for myelin removal process (Myelin Removal Beads II, #130‐096‐733; Miltenyi Biotec). Then, cells were magnetically labeled with CD11b microbeads (#130‐093‐634; Miltenyi Biotec) diluted in PBS supplemented with 0.5% BSA for 15 min in the dark in the refrigerator (2–8°C). CD11b^+^ cells were collected using magnetic field columns (Miltenyi Biotec). Cell suspensions (35 μl) were then plated in a complete medium consisting of DMEM medium (#10569010; Gibco‐BRL) supplemented with 10% fetal bovine serum (FBS; Gibco‐BRL) and containing 40 U/ml penicillin and 40 μg/ml streptomycin (#15140122; Gibco‐BRL) added as a drop in the middle of each well of a Poly‐L‐Lysine (#P4832; Sigma) precoated 8‐well plate (μ‐Slide 8 Well, IBIDI #80826). Cells were incubated for 30 min at 37°C and then 250 μl of complete medium were carefully added to each well. Twenty‐four hours later, we replaced 50% of the complete medium, and we did a full medium change on day 5. The cells were maintained at 37°C in a humidified atmosphere of 5% CO_2_ for 7 DIV.

To assess phagocytosis we used red fluorescent pHrodo *E. coli* bioparticles (#P35361, Thermo Fisher Scientific). At 7 DIV, microglial cells were exposed to the fluorescent beads (0.1 mg of particles/ml) for 1 or 4 h. Following three washes with PBS pH 7.4 to remove all the nonphagocytosed particles, cells were fixed with cold 4% paraformaldehyde for 10 min. After two washes with PBS, fluorescent Bodipy (Molecular Probes BODIPY 493/503, #D3922, Life Technologies) was added (1:1,000) for 10 min at room temperature and protected from the light. DAPI (#D3571, Life Technologies) stain was performed to visualize the cell nuclei. Two more washes with PBS were completed before proceeding to image visualization. Images were obtained with a confocal microscope (Dragonfly confocal microscope, Andor) and were not further processed if not to enhance global signal intensity in the entire images of the same experiment for image presentation purposes using ImageJ or Adobe Photoshop.

### Immunofluorescence in brain tissue sections

Mice were perfused through the heart with 40 ml of cold saline (0.9%) followed by 20 ml of cold 4% paraformaldehyde diluted in phosphate buffer (PB) pH 7.4. The brain was removed, fixed overnight with the same fixative, and immersed in 30% sucrose in PB for cryoprotection for at least 48 h until the brains were completely sunk to the bottom of the tube. After that, brains were frozen in isopentane at −40°C. Cryostat brain sections (14‐μm thick) were fixed in ethanol 70%. In one experiment (Fig EV3), instead of cryostat sections, we used 30‐μm‐thick sections obtained in a vibratome that were processed free‐floating for immunofluorescence. Sections were blocked with 3% normal serum and incubated overnight at 4°C with the primary antibodies: rabbit polyclonal antibodies against P2RY12 (1:250, #AS‐55043A, AnaSpec Inc.) or anti‐PLIN2 (ADPF, 1:200; # PA1‐16972, Invitrogen), and goat polyclonal antibody against Iba‐1 (1:300; #011‐27991; FujiFilm Wako Pure Chemicals Corp.). The secondary antibodies were: Alexa Fluor 488 or 546 (Molecular Probes; Life Technologies S.A.). Cell nuclei were stained with DAPI or To‐Pro3 (Invitrogen). Confocal images were obtained (TCS‐SPE‐II microscope, Leica Microsystems, or Dragonfly, Andor).

### Morphometric analysis of microglia

Images of microglial cells were obtained 4 days postischemia from P2RY12 immunostained vibratome sections of mice priorly subjected or not to microglia depletion/repopulation (*n* = 4 male mice per group). Images were obtained with an oil ×60 objective of a confocal microscope (Dragonfly, Andor). Maximum intensity projections from 21 Z plans were generated with Fiji. The plugin “New Microglia Segmentation and Tracking” available at the Fiji update site “Microglia Morphometry” (https://github.com/embl‐cba/microglia‐morphometry#microglia‐morphometry) was used for semi‐automated segmentation of microglial cells. We analyzed the morphological descriptors: area, circularity, and solidity using Fiji in the ischemic core and periphery and the contralateral hemisphere. For these respective regions, the total number of cells analyzed was 96, 233, and 91 in the group with original microglia, and 58, 178, and 109 in the group with repopulated microglia.

### Transmission electron microscopy

Mice were perfused through the heart with 20 ml of phosphate buffer 0.1 M pH 7.4 followed by 20 ml of the fixative solution (mixture of paraformaldehyde 2% and glutaraldehyde 2.5% in phosphate buffer 0.1 M pH 7.4). Small pieces of 1.5 mm^3^ of cortex from the core and penumbra, and of striatum were obtained and postfixed in osmium tetroxide (1%) and potassium ferrocyanide (0.8%), dehydrated with acetone and embedded in Spurr resin. Ultrathin sections (60–80 nm) were obtained with an ultramicrotome (Leica Ultracut E) using a diamond knife (Diatome). Sections were mounted on copper grids, stained with 2% uranyl acetate for 10 min and lead citrate for 2 min, and observed in a Transmission electron microscope JEOL 1010 with an Orius (Gatan) CCD Camera.

### Western blotting

Mice were euthanized under isoflurane anesthesia brains were carefully removed from the skull and perfused through the heart as above. The ipsilateral and contralateral brain hemispheres were dissected out, immediately frozen on dry ice, and stored at −80°C until further use. Tissue was homogenized in radioimmunoassay precipitation (RIPA) buffer, centrifuged for 20 min at 12,000 × *g* at 4°C and the supernatant was used for protein determination by the Bradford method. Twenty‐five microgram of protein were mixed with loading buffer containing β‐mercaptoethanol and samples were loaded in 10% polyacrylamide gels for electrophoresis. Proteins were transferred to polyvinyl difluoride membranes (Immobilon‐P, #IPVH00010, Millipore/Sigma) and incubated overnight at 4°C with the primary anti‐Plin2 antibody (ADFP rabbit polyclonal antibody, # PA1‐16972, Invitrogen) diluted 1:500, followed by horseradish peroxidase‐conjugated secondary antibody 1 h at RT. β‐Tubulin (#T4026, Sigma‐Aldrich; 1:100,000) was used as loading control. Blots were developed with a chemiluminescent substrate (ECL Amersham, #RPN2235).

### ELISA

Plasma concentration of 17β‐estradiol was measured using an estradiol‐sensitive ELISA (#EIA‐4399; DRG Instruments GmbH, Germany), following the instructions of the manufacturer.

### Human RNA extraction and PCR

Infarcted brain tissue and a corresponding nonaffected region that, when possible, was the mirror region of the contralateral hemisphere, was dissected out at necropsy, immediately frozen, and kept at −80°C. For RNA extraction, we used TrizolVR Reagent (Life Technologies) followed by PureLinkTM RNA Mini Kit (#12183018 A, Invitrogen), and assessed RNA quantity and quality using a ND‐1000 micro‐spectrophotometer (NanoDrop Technologies). Thousand nanogram of total RNA was reverse‐transcribed and the final product was diluted six times in RNAse‐free water. Real‐time quantitative RT–PCR analysis was carried out with Taqman system (#4304437, Life Technology, Carlsbad, CA, USA) using iCycler iQTM Multicolor Real‐Time Detection System (Bio‐Rad). The Taqman primers used were: *PLIN2* (#Hs00605340_m1) and *ISG15* (#Hs01921425_s1). *GAPDH* (#Hs03929097_g1) was used as the housekeeping gene.

### RNA extraction of mouse microglia

RNA was extracted from samples of FACS‐sorted microglia with PicoPure™ RNA Isolation Kit (#KIT0204, Thermo Fisher Scientific) following manufacturer instructions with minor modifications. RNA was precipitated with 70% ethanol. To avoid genomic DNA contamination a DNAse step was performed using Invitrogen™ PureLink™ DNase Set (#12185010, Invitrogen). RNA quantity and purity were assessed with the Pico Kit Assay on the Agilent 2100 Bioanalyzer System.

### RNA sequencing

Libraries were prepared using NEBNext® Poly(A) mRNA Magnetic Isolation Module (#e7490) and NEBNext® Ultra II Directional RNA Library Prep Kit for Illumina (24 reactions #e7760 or 96 reactions #e7765) according to the manufacturer's protocol, to convert total RNA into a library of template molecules of known strand origin and suitable for subsequent cluster generation and DNA sequencing. Briefly, 10–50 ng of total RNA were used for poly(A)‐mRNA selection using poly‐T oligo‐attached magnetic beads using two rounds of purification. During the second elution of the poly‐A RNA, the RNA was fragmented under elevated temperature and randomly primed. Then, the cleaved RNA fragments were copied into first‐strand cDNA using reverse transcriptase. After that, second‐strand cDNA was synthesized, removing the RNA template and synthesizing a replacement strand, incorporating dUTP in place of dTTP to generate ds cDNA using DNA Polymerase I and RNase H. These cDNA fragments, then were “A”‐tailed, NEB hairpin adaptor was ligated and USER enzyme was used to excise the loop on the hairpin adaptors. Finally, PCR selectively enriched those DNA fragments that had adapter molecules on both ends to amplify the amount of DNA in the library and to add specific barcodes to each sample. Final libraries were analyzed using Bioanalyzer DNA 1000 or Fragment Analyzer Standard Sensitivity (# 5067‐1504 or # DNF‐473, Agilent) to estimate the quantity and validate the size distribution and were then quantified by qPCR using the KAPA Library Quantification Kit KK4835 (#07960204001, Roche) prior to the amplification with Illumina's cBot. Libraries were sequenced 1 × 50 + 8 bp on Illumina's HiSeq2500. For RNA with low input (Bodipy^+^ microglia), cDNA was generated using Smart‐seq2 protocol. Briefly, total RNA was reverse‐transcribed using betaine and increasing the magnesium chloride concentration, template switching was using a locked nucleic acid (LNA) and elimination of purification step before preamplification PCR. cDNA was measured with Qubit dsDNA High Sensitivity assay (#Q32851, Invitrogen) to determine the concentration and was analyzed using Agilent Bioanalyzer or Fragment analyzer High Sensitivity assay (#5067‐4626 or DNF‐474, Agilent) to check size distribution profile. Then, from this cDNA libraries were prepared using NEBNext® Ultra DNA Library Prep for Illumina® kit (#E7370) according to the manufacturer's protocol. Briefly, 5 ng of cDNA were fragmented at a range size of 200–500 bp using Covaris S2 and then were subjected to end repair and addition of “A” bases to 3′ ends, ligation of adapters, and USER excision. All purification steps were performed using AgenCourt AMPure XP beads (#A63882, Beckman Coulter). Library amplification was performed by PCR using NEBNext® Multiplex Oligos for Illumina® (96 Unique Dual Index Primer Pairs, #E6440). Final libraries were analyzed using Agilent Bioanalyzer or Fragment analyzer High Sensitivity assay to estimate the quantity and check size distribution and were then quantified by qPCR using the KAPA Library Quantification Kit (#KK4835, KapaBiosystems).

### Transcriptomic analysis

Analyses were performed as previously reported (Lozano‐Ruiz *et al*, 2022). In brief, raw reads were analyzed for data quality using FastQC v0.11.5 (Babraham Bioinformatics) and filtered using skewer v0.2.2 (Jiang *et al*, 2014) for removing the low‐quality reads and trimming the Illumina adapter. STAR program (Dobin & Gingeras, 2015) against Mus musculus genome (GRCm38) was used for mapping the reads followed by the quantification of genes with the RSEM program (Li & Dewey, 2011) using GENCODE m15 reference annotation (Frankish *et al*, 2019). After eliminating genes without an expected value greater than 10 and nonautosomal, we used the TMM method and limma‐voom transformation (Law *et al*, 2014) to normalize the nonbiological variability. Differential expression between different groups was assessed using moderated *t*‐statistics (Ritchie *et al*, 2015). Gene Ontology and Reactome canonical pathway enrichment analysis was performed through GSEA function in cluster Profiler package (gseGO and gsepathway for enrichment analysis and cnetplot graphics) using previously computed *t*‐statistic. Heatmaps and Principal component plots were performed using R statistical software.

### Statistical analyses

Two‐group comparisons were carried out with a two‐tailed Mann–Whitney test or *t*‐test, as required after testing for normality, and adjusting for paired measures using the Wilcoxon matched‐pairs signed‐rank test or paired *t*‐test. Multiple groups were compared with one‐way ANOVA or Kruskal–Wallis test, or two‐way ANOVA, followed by appropriate *post hoc* analyses. In experiments designed to test the difference between young and old mice and whether microglia repopulation in old mice could improve the neurological deficits induced by stroke, the sample size was calculated using G*power 3.1 software (University of Düsseldorf) with an alpha level of 0.05 and a statistical power of 0.95. The effect size (*d* = 1.3) was estimated based on previous data, resulting in groups of *n* = 17 mice. In other experiments, we made estimations based on information on the group mean and SD from previous flow cytometry or RNA data of our own laboratory, and we built from these data the number of animals needed for comparing other outcome measures. For measurements designed as a proof of concept, validation, or internal controls, we used minimum reasonable numbers of animals for confirmatory purposes. The specific tests used in each experiment, *P*‐values, and *n* values are stated in Figure Legends. We used GraphPad Prism software version 9.3.1.

## Author contributions

**Maria Arbaizar‐Rovirosa:** Investigation; methodology. **Jordi Pedragosa:** Formal analysis; investigation; methodology. **Juan J Lozano:** Formal analysis. **Carme Casal:** Methodology. **Albert Pol:** Conceptualization; writing – review and editing. **Mattia Gallizioli:** Conceptualization; formal analysis; supervision; investigation; methodology; writing – review and editing. **Anna M Planas:** Conceptualization; formal analysis; supervision; funding acquisition; writing – original draft; writing – review and editing.

## Disclosure and competing interests statement

The authors declare that they have no conflict of interest.

## Supporting information



Appendix

Expanded View Figures PDF

Table EV1

Dataset EV1

Dataset EV2

Dataset EV3

Dataset EV4

Dataset EV5

Dataset EV6

Dataset EV7

PDF+

## Data Availability

The RNASeq data are accessible from the GEO repository of the National Center for Biotechnology Information, U.S. National Library of Medicine. The accession numbers for these data are GEO: GSE136856 (https://www.ncbi.nlm.nih.gov/geo/query/acc.cgi?acc=GSE136856), GSE196737 (https://www.ncbi.nlm.nih.gov/geo/query/acc.cgi?acc=GSE196737), and GSE209732 (https://www.ncbi.nlm.nih.gov/geo/query/acc.cgi?acc=GSE209732). Other datasets will be found in the repository of our Institution, CSIC, or will be available from the corresponding author upon reasonable request.
