# Supplementary material for: Aged lipid‐laden microglia display impaired responses to stroke
Source: EMBO Mol Med. 2022 Dec 21;15(2):e17175. doi: 10.15252/emmm.202217175 (PMC9906381; doi:10.15252/emmm.202217175)
Supplement: Supplementary file 1 — Appendix [file EMMM-15-e17175-s006.pdf]

Appendix

Aged lipid-laden microglia display impaired responses to stroke

Maria Arbaizar-Rovirosa[1,2], Jordi Pedragosa[1,2], Juan J. Lozano[3], Carme Casal[4], Albert Pol[2,5,6], Mattia Gallizioli[1,2*], Anna M. Planas[1,2,*]

[1] Department of Neuroscience and Experimental Therapeutics, Instituto de Investigaciones Biomédicas de Barcelona (IIBB), Consejo Superior de Investigaciones Científicas (CSIC), Barcelona, Spain

[2] Institut d'Investigacions Biomèdiques August Pi i Sunyer (IDIBAPS), Barcelona, Spain

[3] Bioinformatics platform, Centro de Investigación Biomédica en Red Enfermedades Hepáticas y Digestivas (CIBEREHD), Barcelona, Spain.

[4] Microscopy service, Instituto de Investigaciones Biomédicas de Barcelona (IIBB), Consejo Superior de Investigaciones Científicas (CSIC), Barcelona, Spain

[5] Department of Biomedical Sciences, Faculty of Medicine, Universitat de Barcelona, 08036, Barcelona, Spain.

[6] Institució Catalana de Recerca i Estudis Avançats (ICREA), 08010, Barcelona, Spain.

Table of Contents

1. Appendix Fig. S1
2. Appendix Fig. S2
3. Appendix Fig. S3

Appendix Fig. S1

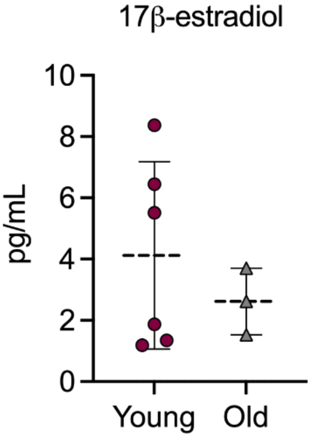

Appendix Fig. S1. 17β-estradiol concentration in plasma of young (n=6) and old (n=3) control female mice show large coefficient of variation (74%) in the young group likely attributable to different phases of the estrus cycle.

Appendix Fig. S2

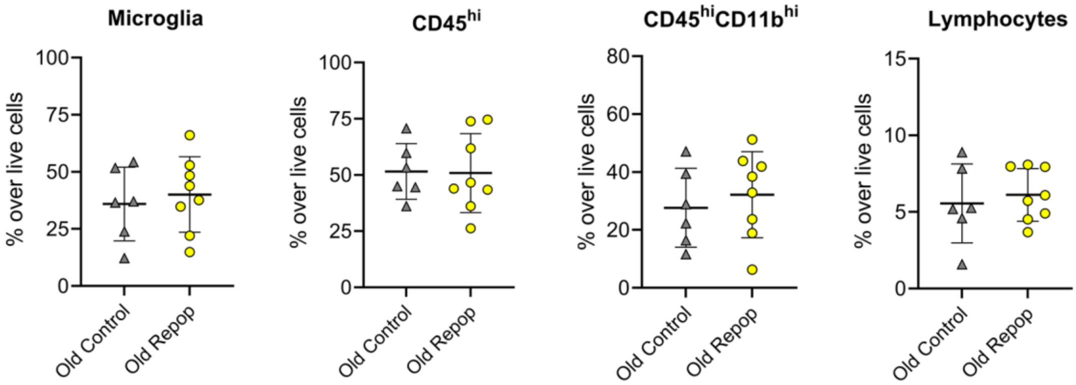

Appendix Fig. S2. Microglia and brain infiltrating cells four days post-ischemia in old female mice under control diet (n=6, Old Control) or following microglia depletion/repopulation for seven days prior to ischemia (n=8, Old Repop). There were no differences between groups regarding the % of cells. Values show data for individual mice and the mean ± SD.

Appendix Fig. S3.

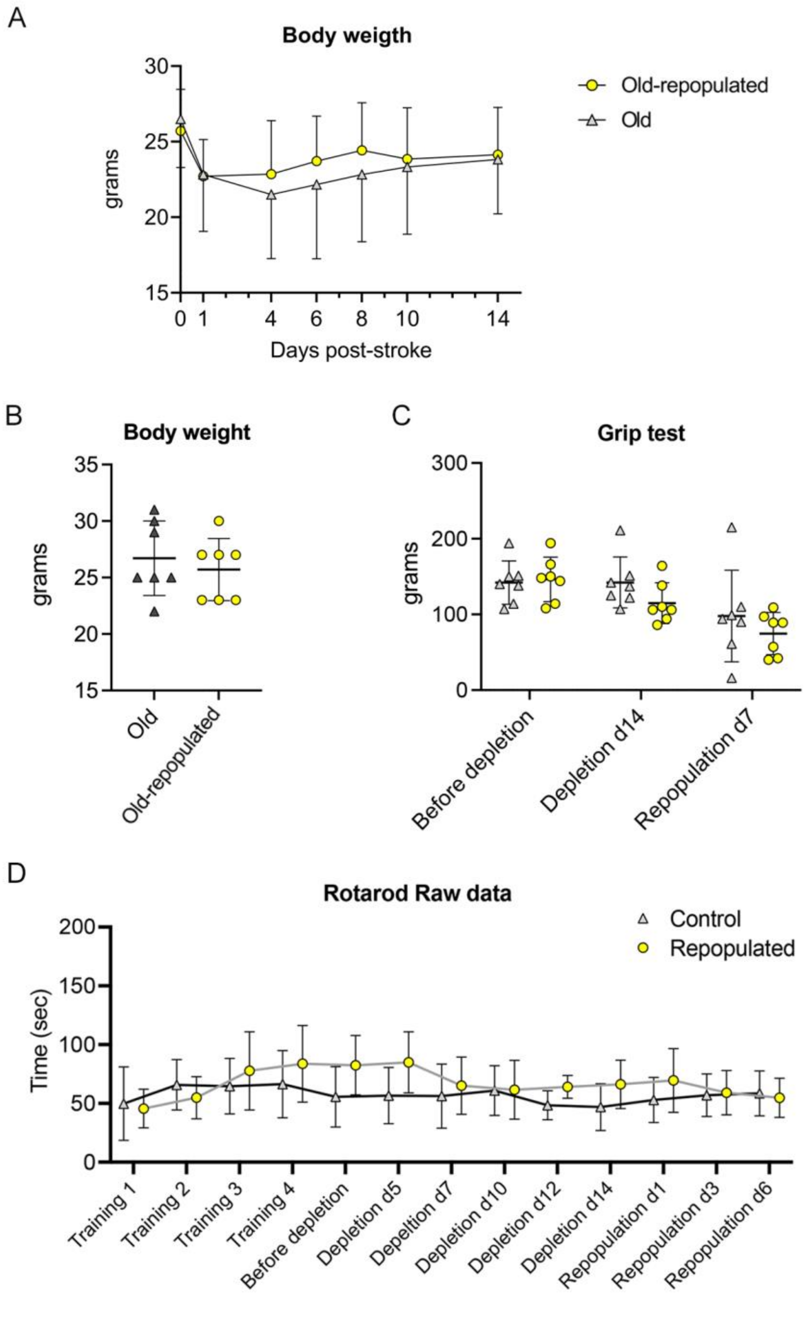

Appendix Fig. S3. Behavioral analysis of old mice with or without microglia depletion/repopulation. *Related to Fig. 6.* Old female mice received PLX5622 diet for two weeks for microglia depletion followed by control diet for another week for microglia repopulation before induction of ischemia (n=7). Treatment controls received the corresponding control diet all the time (n=7). Mice were studied for 2 weeks more after ischemia. A) Mean body weight evolution after induction of ischemia in both groups shows no differences. B) Body weight after microglia repopulation (prior to ischemia) in old mice was similar in both groups regardless of the diet, as shown for each individual mouse. One mouse of the control group died after day 1 post-ischemia and was excluded from A and B graphs. C, D) Behavioral tests performed in these mice prior to ischemia showed no significant differences between groups for the grip (C) and rotarod (D) tests during treatments (two-way ANOVA, treatment effect p=0.30 and p=0.34, respectively). Values show data for individual mice and/or show the mean±SD.
