# Supplementary material for: Aged lipid‐laden microglia display impaired responses to stroke
Source: EMBO Mol Med. 2022 Dec 21;15(2):e17175. doi: 10.15252/emmm.202217175 (PMC9906381; doi:10.15252/emmm.202217175)
Supplement: Supplementary file 2 — Expanded View Figures PDF [file EMMM-15-e17175-s009.pdf]

Expanded View Figures

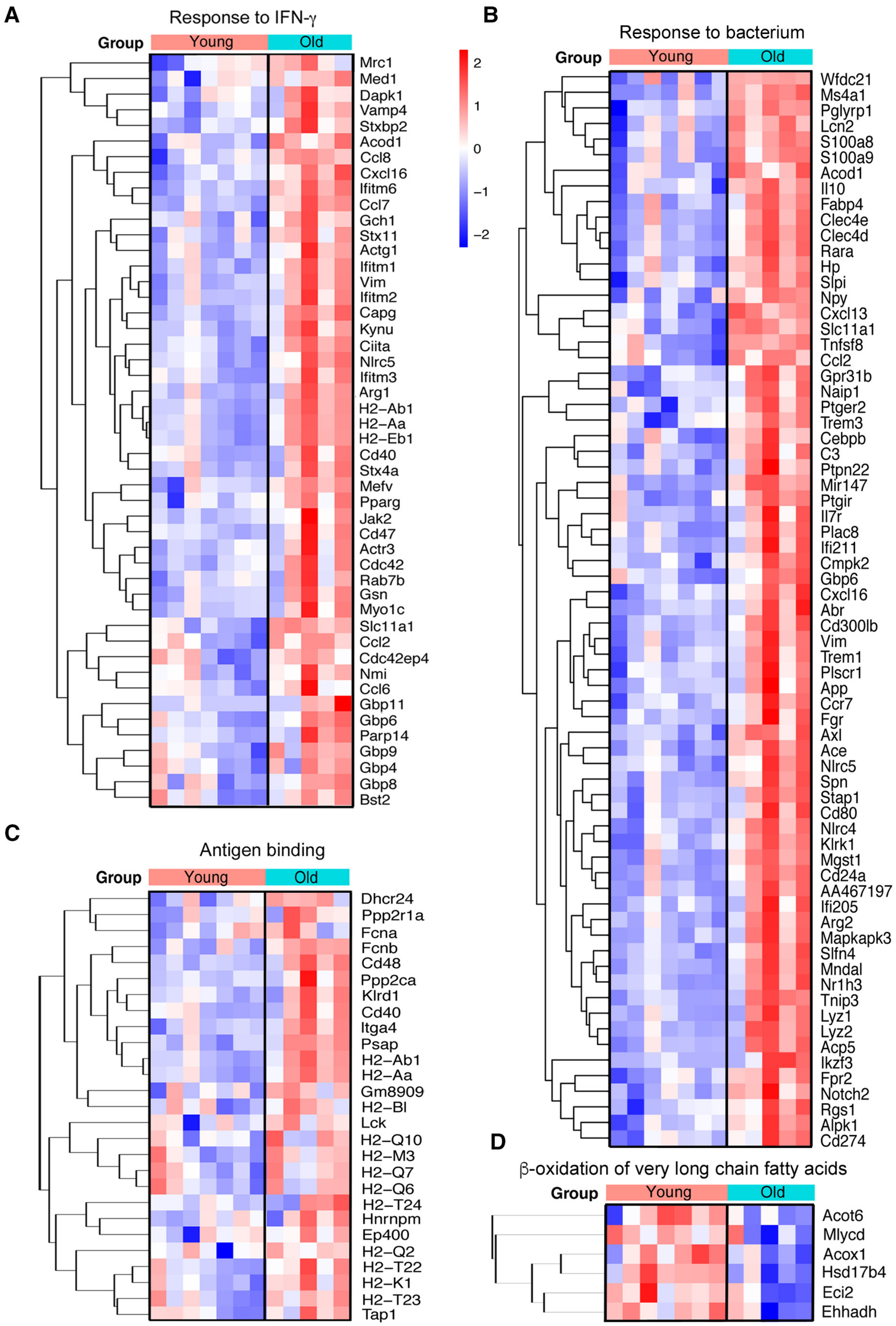

Figure EV1.

 © 2022 The Authors

Figure EV1. Enrichment of genes in GO pathways in microglia of old versus young ischemic female mice. Related to Fig 3.

RNAseq analysis of microglia obtained by FACS from the brain of old (*n* = 5) versus young (*n* = 7) female mice 4 days after ischemia (GSE196737).

A–C Heatmaps illustrate genes upregulated in microglia of old ischemic mice versus young ischemic female mice for the following GO terms: “Response to IFN-γ” (A), “Response to bacterium” (B), and “Antigen binding” (C).

D By contrast, downregulated pathways in microglia of old mice included: “β-oxidation of very long-chain fatty acids”.

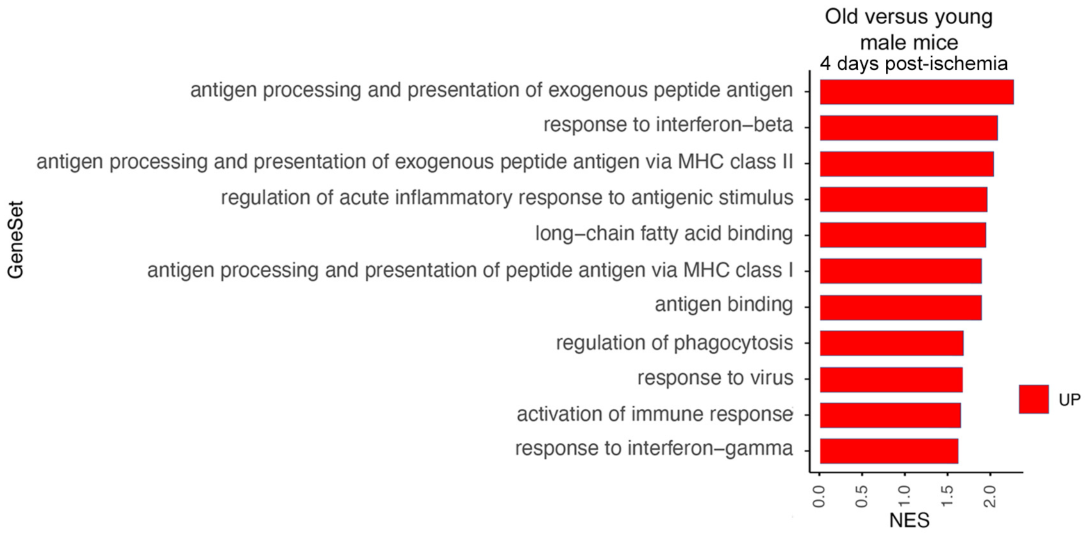

Figure EV2. Enrichment of genes in GO pathways involved responses in microglia of old versus young ischemic male mice. Related to Fig 3.

A transcriptomic analysis (GSE209732) was performed to validate that the main pathways found upregulated in old female mice were also upregulated in old male mice 4 days after ischemia. GO pathways following RNAseq analysis of microglia (Bodipy$^+$) obtained by FACS from the brain of old (*n* = 3) versus young (*n* = 4) male mice 4 days after ischemia.

Figure EV3. Microglia cells after depletion/repopulation. Related to Fig 4.

A, B (A) Representative images of the striatum of young female mice with original microglia and repopulated microglia 4 days postischemia (*n* = 4 per group). Images show microglial cells immunostained with anti-P2YR12 (red) in the noninjured contralateral hemisphere, the periphery of ischemia, and the lesion core. Cell nuclei are labeled with DAPI (blue). After repopulation, original and renewed microglia react to ischemia with changes in morphology. (B) Magnification of individual cells marked with squares in (D). Scale bar: (A) 20 μm, (B) 10 μm.

C Morphometric analysis of microglial cells showed reduced area and increased solidity and circularity in microglia of the ischemic hemisphere versus the contralateral hemisphere in both groups. However, the increase in circularity at the periphery and core of infarction was attenuated in repopulated versus original microglia (**P* = 0.047 in the periphery and **P* = 0.033 in the core; Kruskal–Wallis test). Points show individual cells (in the periphery, core, and contralateral regions: *n* = 233, 96, and 91 cells for the control group, and *n* = 178, 58, and 109 cells for the renewed group, respectively), and colors indicate different mice (*n* = 4 mice per group). Bars show the median with 95% confidence interval.

D Renewed microglia derive from brain cells. We generated chimeric mice by bone marrow transplantation from DsRed fluorescent reporter donor male mice to wild-type recipient male mice (*n* = 11). After at least 8 weeks, mice were treated with PLX5622 in the diet (*n* = 8) or corresponding control diet (*n* = 3). Three weeks later, mice were euthanized (*n* = 4) or they were switched to control diet for repopulation for 7 days (*n* = 4 per group). The brain was studied via flow cytometry by measuring DsRed$^+$ and DsRed$^-$ cells in the gate of microglia cells. Absolute number of microglial cells was strongly reduced after microglia depletion (PLX5622 diet; one-way ANOVA and Holm–Šídák’s multiple comparisons test ****P* = 0.0002 versus control diet). Microglia numbers recovered after 7 days of repopulation ($^{\&\&\&}P$ = 0.0002 versus depleted cells). The % of DsRed cells in the microglia gate of mice fed control diet was negligible. However, after microglia depletion (PLX5622 treatment; ANOVA and Holm–Šídák’s, ****P* < 0.0001 versus control diet) there was a high % of DsRed$^+$ cells within the very small population of CD45^{low}CD11b$^+$ cells indicating the presence of a few CSF1R-independent infiltrating cells. Importantly, the proportion of DsRed$^+$ cells was negligible after mice were switched to control diet and the number of microglia increased ($^{\&\&\&}P$ < 0.0001 versus depleted mice). Values are expressed as the mean ± SEM.

© 2022 The Authors

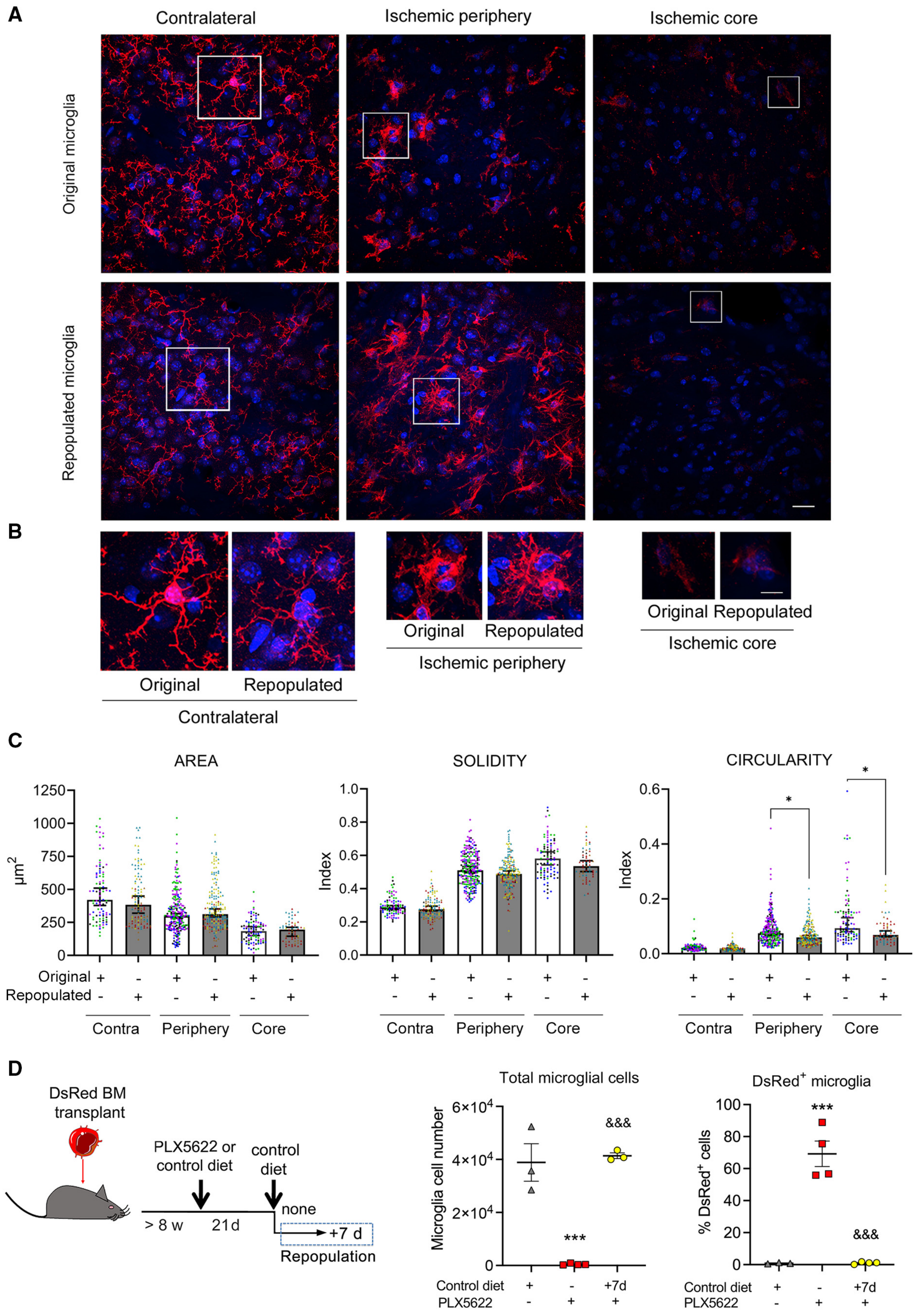

Figure EV3.

© 2022 The Authors

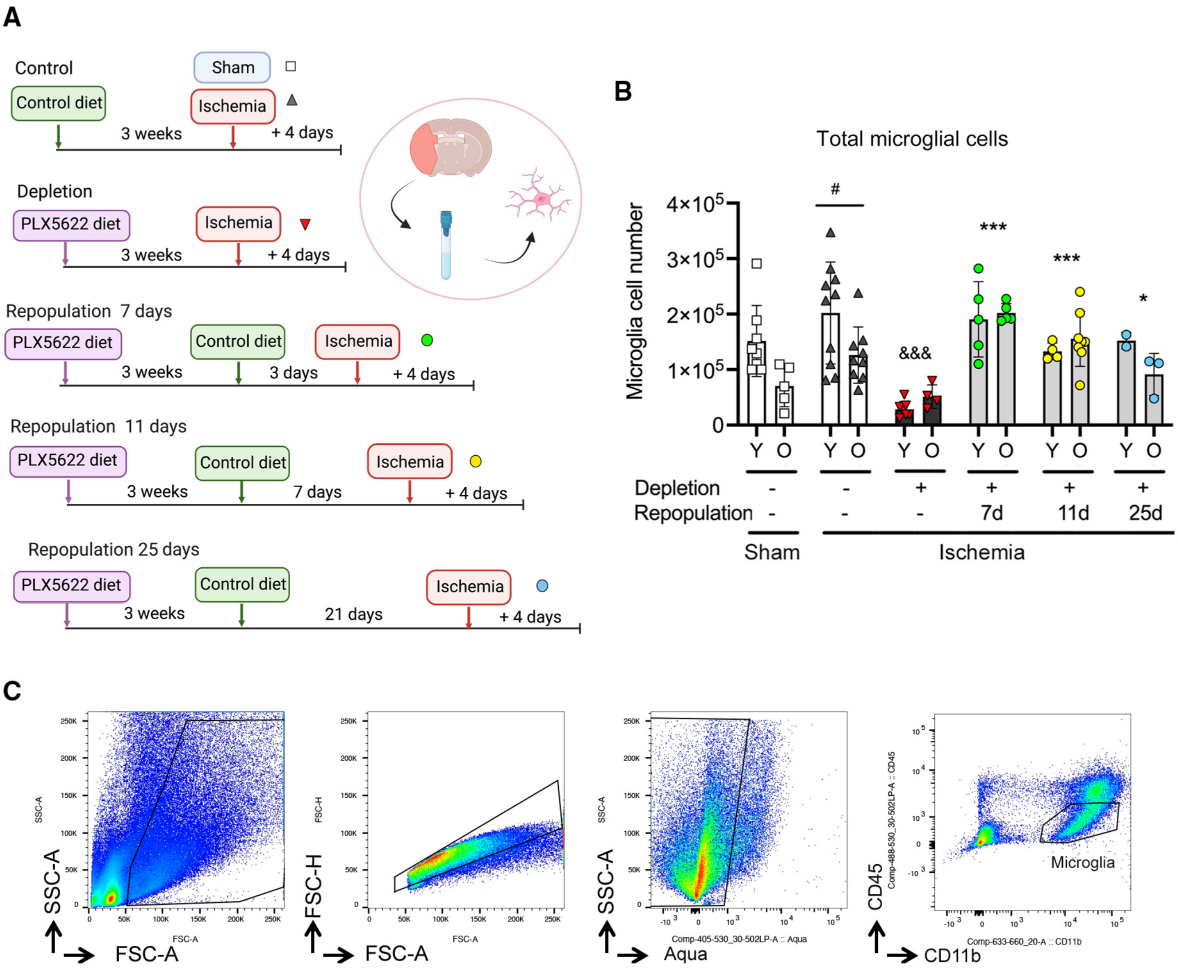

Figure EV4. Effects of microglia repopulation. Related to Fig 4.

A Experimental design for microglia depletion and repopulation in young (3–4 months; $n = 39$) and old (20–22 months; $n = 34$) female mice. We depleted microglia via a PLX5622 diet for 3 weeks. Mice were repopulated by switching to the corresponding control diet for 3, 7, or 21 days prior to ischemia induction. The brain was studied 4 days postischemia and during this period control diet was maintained. Total repopulation times: 7, 11, and 25 days, respectively. As controls, we used mice subjected to control diet and studied 4 days postischemia or sham operation.

B The number of microglia recovered postischemia was lower in old (O) than in young (Y) mice fed a control diet (two-way ANOVA and Šídák's multiple comparisons test, $\#P = 0.0134$). The PLX5622 diet strongly reduced the number of microglial cells postischemia in both age groups ($^{\&\&\&}P < 0.0001$ versus ischemic mice on control diet); switching to control diet increased the number of microglia versus depleted mice ($^{***}P < 0.0001$ at day 7, $^{***}P = 0.0003$ at day 11, and $^{*}P = 0.0377$ at day 25; two-way ANOVA and Dunnett's multiple comparison test). Ischemic mice: $n = 10$ Y and $n = 9$ O with control diet; $n = 7$ Y and $n = 4$ O with PLX5622 diet (depleted); $n = 5$ Y and $n = 5$ O with PLX5622 diet + 7 d (repopulated for 7 days); $n = 4$ Y and $n = 8$ O with PLX5622 diet + 11 d (repopulated for 11 days); $n = 2$ Y and $n = 3$ O with PLX5622 diet + 25 d (repopulated for 25 days). Sham mice: $n = 8$ Y and $n = 5$ O.

C Gating strategy for cell sorting to obtain the $CD45^{low}CD11b^{+}$ microglia shown in (B).

Figure EV5. Enrichment of genes in GO pathways in renewed microglia of old ischemic mice versus the original microglia of old ischemic mice. Related to Fig 4. ▶

RNAseq analysis of microglia obtained by FACS from the brain of old mice with repopulated microglia (Old-renewed; $n = 8$) versus old mice with the original microglia ($n = 5$) 4 days after ischemia.

A–E Heatmaps illustrate DEGs in various GO pathways downregulated (A–C) and upregulated (D, E) in renewed microglia of old mice after ischemia in relation to the original microglia of old ischemic mice. Downregulated GO terms include: (A) "Response to type I IFN," (B) "Pyroptosis," and (C) "Antigen binding." By contrast, enriched pathways in renewed microglia include: (D) "Mitochondrial respirasome" and (E) "Fatty acid metabolism".

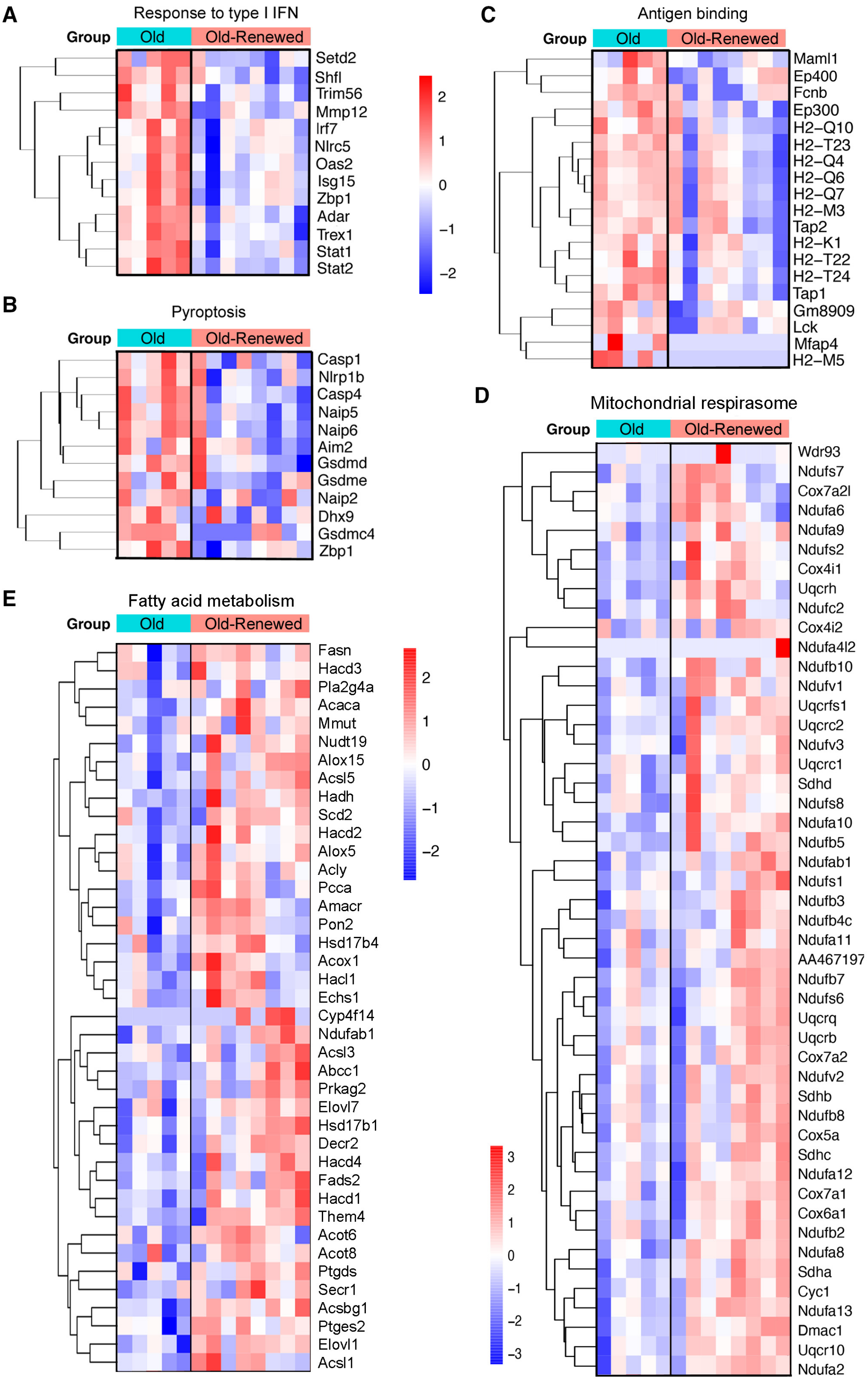

Figure EV5.

© 2022 The Authors
