# Supplementary material for: Aged lipid‐laden microglia display impaired responses to stroke
Source: EMBO Mol Med. 2022 Dec 21;15(2):e17175. doi: 10.15252/emmm.202217175 (PMC9906381; doi:10.15252/emmm.202217175)
Supplement: Supplementary file 3 — Table EV1 [file EMMM-15-e17175-s011.pdf]

Table EV1. Features of stroke patients.

Age	Gender	Admission NIHSS	Vascular territory	Intracranial oclusion site	Acute revascularization therapy	Hemorrhagic transformation	Etiology	Exitus (days after onset)	Exitus to necropsy timelapse
89	Woman	9	Vertebro-basilar infarct	No vessel imaging	None	No	Cardio-embolic	1	3 h
63	Woman	20	Right MCA infarct	Right M1	Yes mechanical thrombectomy	No	Undetermined	1	2 h
81	Woman	13	Right MCA infarction Left MCA and IH2 recurrence	Right and Left M1	None	No	Undetermined	4	8 h
86	Woman	1	Vertebro-basilar infarct	Basilar artery	None	No	Cardio-embolic	3	3 h
88	Woman	19	Right MCA infarction Left MCA and IH2 recurrence	Right and Left M1	Yes	No	Cardio-embolic	5	5:45 h
79	Man	4	Left MCA infarct	Left M2	None	Parieto-occipital parenchymal hematoma type 2	Large vessel disease	5	4:35 h
86	Woman	20	Left MCA infarct	Left M1	None	No	Cardio-embolic	6	2h 20min
74	Woman	35	Left MCA infarct	No vessel imaging	None	No	Undetermined	18	3 h
57	Woman	11	Right PICA infarct	Right vertebral artery	None	No	Other: dissection	140	7 h
