# Supplementary material for: Aged lipid‐laden microglia display impaired responses to stroke
Source: EMBO Mol Med. 2022 Dec 21;15(2):e17175. doi: 10.15252/emmm.202217175 (PMC9906381; doi:10.15252/emmm.202217175)
Supplement: Supplementary file 12 [file EMMM-15-e17175-s002.pdf]

EMBO Molecular Medicine

Aged lipid-laden microglia display impaired responses to stroke

Maria Arbaizar-Rovirosa, Jordi Pedragosa, Juan Lozano, Carme Casal, Albert Pol, Mattia Gallizioli, and Anna Planas
DOI: 10.15252/emmm.202217175

Corresponding author(s): Anna Planas (anna.planas@iibb.csic.es) , Mattia Gallizioli (mattia.gallizioli@iibb.csic.es)

Review Timeline:

Submission Date:	23rd Mar 22
Editorial Decision:	4th May 22
Revision Received:	2nd Aug 22
Editorial Decision:	17th Aug 22
Revision Received:	15th Nov 22
Editorial Decision:	29th Nov 22
Revision Received:	30th Nov 22
Accepted:	1st Dec 22

Editor: Jingyi Hou

Transaction Report:

(Note: With the exception of the correction of typographical or spelling errors that could be a source of ambiguity, letters and reports are not edited. Depending on transfer agreements, referee reports obtained elsewhere may or may not be included in this compilation. Referee reports are anonymous unless the Referee chooses to sign their reports.)

4th May 2022

Thank you again for submitting your work to EMBO Molecular Medicine. We have now heard back from two of the three referees who agreed to evaluate your manuscript. Unfortunately, after a series of reminders, we did not obtain a report from Referee #2. In the interest of time, I prefer to make a decision now rather than further delay the process. As you will see from the reports below, the referees acknowledge the potential interest of the study. However, they raise a series of concerns, which we would ask you to address in a major revision of the manuscript.

I think that the referees' recommendations are relatively straightforward, so there is no need to reiterate their comments. In particular, Referee #3 raised a series of concerns regarding mechanisms and causality. During our pre-decision cross-commenting process (in which referees are given a chance to make additional comments, including on each other's reports), Referee #1 agreed with Referee #3's assessment and mentioned an essential set of experiments that need to be performed. I have included these comments below after the referee reports. Further, in light of Referee #3's criticism, the mechanism should be strengthened, and overinterpretation should be avoided.

We would welcome the submission of a revised version within three months for further consideration. Please note that EMBO Molecular Medicine in principle only allows a single round of revision. As acceptance or rejection of the manuscript will depend on another round of review, your responses should be as complete as possible.

EMBO Molecular Medicine has a "scooping protection" policy, whereby similar findings that are published by others during review or revision are not a criterion for rejection. Should you decide to submit a revised version, I do ask that you get in touch after three months if you have not completed it to update us on the status.

We are aware that many laboratories cannot function at full efficiency during the current COVID-19/SARS-CoV-2 pandemic and have therefore extended our "scooping protection policy" to cover the period required for a full revision to address the experimental issues. Please let me know should you need additional time, and also if you see a paper with related content published elsewhere.

Please read below for important editorial formatting and consult our author's guidelines for proper formatting of your revised article for EMBO Molecular Medicine.

I look forward to receiving your revised manuscript.

Use this link to login to the manuscript system and submit your revision:
https://embomolmed.msubmit.net/cgi-bin/main.plex

Kind regards
Jingyi

Jingyi Hou
Editor
EMBO Molecular Medicine

*****

When submitting your revised manuscript, please carefully review the instructions that follow below. We perform an initial quality control of all revised manuscripts before re-review; failure to include requested items will delay the evaluation of your revision.

We require:

1) A .docx formatted version of the manuscript text (including legends for main figures, EV figures and tables). Please make sure that the changes are highlighted to be clearly visible.

2) Individual production quality figure files as .eps, .tif, .jpg (one file per figure). For guidance, download the 'Figure Guide PDF':
(https://www.embopress.org/page/journal/17574684/authorguide#figureformat).

3) A .docx formatted letter INCLUDING the reviewers' reports and your detailed point-by-point responses to their comments. As part of the EMBO Press transparent editorial process, the point-by-point response is part of the Review Process File (RPF), which will be published alongside your paper.

4) A complete author checklist, which you can download from our author guidelines (https://www.embopress.org/page/journal/17574684/authorguide#submissionofrevisions). Please insert information in the checklist that is also reflected in the manuscript. The completed author checklist will also be part of the RPF.

5) Please note that all corresponding authors are required to supply an ORCID ID for their name upon submission of a revised manuscript.

6) It is mandatory to include a 'Data Availability' section after the Materials and Methods. Before submitting your revision, primary datasets produced in this study need to be deposited in an appropriate public database, and the accession numbers and database listed under 'Data Availability'. Please remember to provide a reviewer password if the datasets are not yet public (see https://www.embopress.org/page/journal/17574684/authorguide#dataavailability).

In case you have no data that requires deposition in a public database, please state so in this section. Note that the Data Availability Section is restricted to new primary data that are part of this study.

7) For data quantification: please specify the name of the statistical test used to generate error bars and P values, the number (n) of independent experiments (specify technical or biological replicates) underlying each data point and the test used to calculate p-values in each figure legend. The figure legends should contain a basic description of n, P and the test applied. Graphs must include a description of the bars and the error bars (s.d., s.e.m.). See also 'Figure Legend' guidelines:

https://www.embopress.org/page/journal/17574684/authorguide#figureformat

8) We would also encourage you to include the source data for figure panels that show essential data. Numerical data should be provided as individual .xls or .csv files (including a tab describing the data). For blots or microscopy, uncropped images should be submitted (using a zip archive if multiple images need to be supplied for one panel). Additional information on source data and instruction on how to label the files are available at

<https://www.embopress.org/page/journal/17574684/authorguide#sourcedata>.

9) Our journal encourages inclusion of *data citations in the reference list* to directly cite datasets that were re-used and obtained from public databases. Data citations in the article text are distinct from normal bibliographical citations and should directly link to the database records from which the data can be accessed. In the main text, data citations are formatted as follows: "Data ref: Smith et al, 2001" or "Data ref: NCBI Sequence Read Archive PRJNA342805, 2017". In the Reference list, data citations must be labeled with "[DATASET]". A data reference must provide the database name, accession number/identifiers and a resolvable link to the landing page from which the data can be accessed at the end of the reference. Further instructions are available at <https://www.embopress.org/page/journal/17574684/authorguide#referencesformat>.

10) We replaced Supplementary Information with Expanded View (EV) Figures and Tables that are collapsible/expandable online. A maximum of 5 EV Figures can be typeset. EV Figures should be cited as 'Figure EV1, Figure EV2" etc... in the text and their respective legends should be included in the main text after the legends of regular figures.

- For the figures that you do NOT wish to display as Expanded View figures, they should be bundled together with their legends in a single PDF file called *Appendix*, which should start with a short Table of Content. Appendix figures should be referred to in the main text as: "Appendix Figure S1, Appendix Figure S2" etc.

- Additional Tables/Datasets should be labeled and referred to as Table EV1, Dataset EV1, etc. Legends have to be provided in a separate tab in case of .xls files. Alternatively, the legend can be supplied as a separate text file (README) and zipped together with the Table/Dataset file.

See detailed instructions here:

<https://www.embopress.org/page/journal/17574684/authorguide#expandedview>.

11) The paper explained: EMBO Molecular Medicine articles are accompanied by a summary of the articles to emphasize the major findings in the paper and their medical implications for the non-specialist reader. Please provide a draft summary of your article highlighting

- the medical issue you are addressing,

- the results obtained and

- their clinical impact.

This may be edited to ensure that readers understand the significance and context of the research. Please refer to any of our published articles for an example.

12) For more information: There is space at the end of each article to list relevant web links for further consultation by our readers. Could you identify some relevant ones and provide such information as well? Some examples are patient associations, relevant databases, OMIM/proteins/genes links, author's websites, etc...

13) Author contributions: the contribution of every author must be detailed in a separate section (before the acknowledgments).

14) A Conflict of Interest statement should be provided in the main text.

15) Every published paper now includes a 'Synopsis' to further enhance discoverability. Synopses are displayed on the journal webpage and are freely accessible to all readers. They include a short stand first (maximum of 300 characters, including space) as well as 2-5 one-sentences bullet points that summarizes the paper. Please write the bullet points to summarize the key NEW findings. They should be designed to be complementary to the abstract - i.e. not repeat the same text. We encourage inclusion of key acronyms and quantitative information (maximum of 30 words / bullet point). Please use the passive voice. Please attach these in a separate file or send them by email, we will incorporate them accordingly.

Please also suggest a striking image or visual abstract to illustrate your article as a PNG file 550 px wide x 300-600 px high.

EMBO Molecular Medicine has a "scooping protection" policy, whereby similar findings that are published by others during review or revision are not a criterion for rejection. Should you decide to submit a revised version, I do ask that you get in touch after three months if you have not completed it, to update us on the status.

***** Reviewer's comments *****

Referee #1 (Comments on Novelty/Model System for Author):

Please refer in remarks to author

Referee #1 (Remarks for Author):

In this manuscript titled "Age-dependent lipid droplet-rich microglia worsen stroke outcome in old mice", Maria Arbaizar-Rovirosa and colleagues investigated for the role of age-associated increase in lipid droplet-rich microglia on post-stroke recovery using a mouse model. Briefly, the authors show microglial cells undergo transcriptomic and phenotypic changes after ischemic stroke using young mice. Using RNAseq analysis on flow sorted microglia and by performing bioinformatics using Gene Ontology, GSEA and KEGG pathway analysis authors show changes in key pathways including upregulation of innate immune pathways in microglia

following stroke. Stroke in young mice induces the formation of lipid droplets in microglia. Microglial cells from aged brain express increased lipid droplet content compared to young brain in controls and after stroke. Data from transmission electron microscopy, flow analysis and in vitro studies show microglia of young mice accumulate lipid droplets after brain ischemia. Authors show that flow sorted microglia from aged females demonstrate impaired phagocytosis compared to young mice, and aged stroke mice show worse neurological outcomes compared to young. Further, depleting microglia by a diet containing the CSF1R antagonist PLX5622 for three weeks and allowing to repopulate prior to stroke helps restore microglial transcriptional profiles and eliminates the age-associated increase in lipid droplets. Finally, the authors show chronic effects of microglia repopulation in aged mice on stroke recovery.
The idea that microglial lipid droplets contribute to stroke recovery is exciting, manuscript well written, authors employed an array of techniques and the topic is timely. My enthusiasm is somewhat reduced as provided data did not sufficiently support the hypothesis and some flaws in study design.

Major concern:
1. Young females develop smaller infarcts compared to older females following stroke, this was demonstrated in several earlier studies and is largely estrogen mediated. Surprisingly, this is not seen in this study, Fig 3A shows no difference in injury? Authors should discuss potential role of E2 as Fig 3-5 are performed using young female mice.
2. This is also important as estrogens are known to modulate activation of microglia in dose dependent manner (Vegeto E, et al., PNAS 2003) and it also regulates the activity of enzymes involved in the fatty acid synthesis and regulating brain fatty acid levels (as reviewed in: Morselli E, et al., Am J Physiol Endocrinol Metab.. 2018). Some of the observed changes in transcriptional profile of microglia are likely due to E2 levels in young females than age-associated. The authors need to expand on this and at least validate, if microglial DEGs are indeed age-associated using ovariectomized young females or in a cohort of male mice.

Minor comments:
1. Provide quantification data for Fig 2B (western blot)
2. Blinding for histological analysis is not mentioned.
3. Main stroke outcomes data shows, no difference in infarct and modest functional recovery at day 14 (much later compared to lipid droplet accumulation after stroke). Title needs to be revised appropriately as provided data do not fully support the claim, particularly claiming causality where there is only association.
4. Please also perform cresyl violet/nissl staining to complement MRI quantification data from 3B and 6C. Is % infarct volume presented on top panel represents analysis of day 4/14?

Referee #3 (Remarks for Author):

This study examined if microglial renewal can restore microglia function and reverse post-stroke neurological deficits. The outcome measures the authors employed for microglial function was primarily transcriptome based, along with in vitro phagocytosis assay and intracellular lipid phenotype assessment. In general, the manuscript was well written and appropriately presented, albeit some fonts within figures were too small. However, there are some concerns that need to be addressed prior to reaching the proposed conclusion:

1) The manuscript is structured to show the effect of stroke on young, then in aged, then aged vs renewed. Although this enables fluidity in readership, the way that the data presented does not allow for examining the combined impact of aging and stroke. For instance, lipids are abundantly present already in aged microglia (Fig. 3F control), do these cells have heightened inflammatory transcriptomic signature compared to young, and further exacerbated after stroke?

2) The causality of greater intracellular lipids --> type 1 IFN gene signature --> lack of phagocytosis --> worse stroke outcome is not evident. Currently, the data is associative. The discrepancy of this proposed sequence of the events is highlighted in the setting of young, which needs lipid generation for appropriate innate immune response and phagocytosis. Therefore, it is unclear what new information the findings of this manuscript has revealed. What is the mechanism of elevated lipids inducing type 1 IFN signature in aged after stroke?

3) Many occasions throughout the manuscript the authors mentioned "subset of microglia" - what subset is this? Similarly, if microglial repopulation is dependent on PLX5622-resistant microglia, do these microglia have less lipids thus allowing for the renew population to also have lower lipid content? What makes them resistant to PLX5622? With the greater knowledge of different microglia subsets nowadays, and how they can be identified, delineation of the remaining microglia involved in the renewal process should be achievable.

4) It is interesting that the authors put a lot of emphasis on phagocytosis as a microglial function (the only one they assessed functionally), but microglia has other important functions too especially with their interactions with neurons and neurovascular units. These were not assessed at all, what is rationale behind this? What is the effect of aging and repopulation/renew on these functions?

5) Whilst transcriptomic assessment is useful and obviously "very hot" in recent years - what is the implication of microglia renewal in the general brain health (although infarct size is not changed) - ie. astrocytic activation and scarring? Cerebral inflammatory environment? Neuronal rewiring?

6) Improvement of stroke outcome after microglial repopulation is arguable - only Neuroscore really showed improvement and this assessment is not fully dependent on neurological functions but also general health. It bets the question whether the effect seen in this study is not specific to microglia, but rather involvement of peripheral CSF1R-dependent cells too - doi.org/10.1073/pnas.1922788117.

7) Does microglia repopulation occur in the stroke infarct or surrounding regions after stroke? If so, do these repopulated cells demonstrate renewed phenotypes observed?

8) What is the clinical relevance of the findings?

****

Additional comments from Referee #1:

"I very much agree with Referee #3 comments. If the authors can carefully revise manuscript as per review comments, it would substantially improve the manuscript and would make it more suitable for publication in EMBO Mol Med.

Here are few experiments that the authors can perform to improve the study:

1. Lack of neuroprotection in young females (Fig 3A) compared to aged needs more justification. Authors should provide estrogen levels data from plasma/serum samples of these young and aged mice.

2. Some of the observed changes in transcriptional profile of microglia (Fig 3-5) are likely due to E2 levels in young females than age-associated. The authors need to expand on this and at least validate, if microglial DEGs are indeed age-associated using ovariectomized young females or in a cohort of male mice.

3. Authors should consider testing post-stroke recovery in more sensitive and/or chronic behavioral tests for 30d or longer to test for beneficial effects of renewed microglia. Provided data from behavioral tasks is a major weakness and suggests no major clinical relevance from renewed microglia

4. Clinical relevance would improve if findings from Fig 2C and/or 3G were validated using patient samples.

Answers to Reviewers

We thank the Reviewers for the constructive comments and the Editor for giving us the opportunity to reply. Below, we provide answers to all the points.

Referee #1:
In this manuscript titled "Age-dependent lipid droplet-rich microglia worsen stroke outcome in old mice", Maria Arbaizar-Rovirosa and colleagues investigated for the role of age-associated increase in lipid droplet-rich microglia on post-stroke recovery using a mouse model. Briefly, the authors show microglial cells undergo transcriptomic and phenotypic changes after ischemic stroke using young mice. Using RNAseq analysis on flow sorted microglia and by performing bioinformatics using Gene Ontology, GSEA and KEGG pathway analysis authors show changes in key pathways including upregulation of innate immune pathways in microglia following stroke. Stroke in young mice induces the formation of lipid droplets in microglia. Microglial cells from aged brain express increased lipid droplet content compared to young brain in controls and after stroke. Data from transmission electron microscopy, flow analysis and in vitro studies show microglia of young mice accumulate lipid droplets after brain ischemia. Authors show that flow sorted microglia from aged females demonstrate impaired phagocytosis compared to young mice, and aged stroke mice show worse neurological outcomes compared to young. Further, depleting microglia by a diet containing the CSF1R antagonist PLX5622 for three weeks and allowing to repopulate prior to stroke helps restore microglial transcriptional profiles and eliminates the age-associated increase in lipid droplets. Finally, the authors show chronic effects of microglia repopulation in aged mice on stroke recovery.The idea that microglial lipid droplets contribute to stroke recovery is exciting, manuscript well written, authors employed an array of techniques and the topic is timely. My enthusiasm is somewhat reduced as provided data did not sufficiently support the hypothesis and some flaws in study design.

Major concern:
1. Young females develop smaller infarcts compared to older females following stroke, this was demonstrated in several earlier studies and is largely estrogen mediated. Surprisingly, this is not seen in this study, Fig 3A shows no difference in injury? Authors should discuss potential role of E2 as Fig 3-5 are performed using young female mice.

We thank the Reviewer for raising this important point since we focused on the worse neurological deficit in the old mice, and not as much on infarct volume. Based on literature of stroke in young female mice, we anticipated a smaller variation in this group and estimated a sample size that now, in view of the actual variability of the initial group, we realize it was too small to compare infarct volumes. We did not exclude any mouse, but two mice in the old group died after stroke whereas none of the young mice died. A possible difference with other studies in the literature, is that in our study the mice were awake during the occlusion time (see Methods, page 17). Therefore, reduced anesthesia could also result in larger infarct volumes. In any case, to limit type I errors in our infarct volume study, we recalculated the sample size and estimated a new effect size d of 1.3 using an alpha error of 0.05 and a power of 0.95, resulting in a sample size of n=17 mice per group. Now, after increasing the number of mice, the median % infarct volume is 10.0% in young mice and 26.2% in old mice (Mann-Whitney test, p=0.041) showing higher infarct volume in the old group, as expected (Fig. 3A).

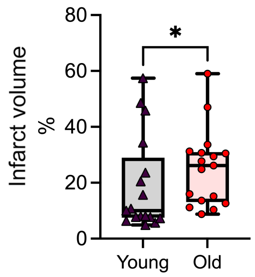

In addition, we added this text in the Discussion (Page 9):
'Although we detected lipid droplets in ischemic microglia and old microglia of male and female mice, estrogens affect lipid metabolism[35] and microglial activity[36]. Therefore, estrogen levels and estrogen treatments are expected to influence the reported microglia responses in females.'

References:
35. Morselli E, Santos RS, Gao S, Ávalos Y, Criollo A, Palmer BF, Clegg DJ. Impact of estrogens and estrogen receptor-α in brain lipid metabolism. Am J Physiol Endocrinol Metab. 2018 Jul 1;315(1):E7-E14. doi: 10.1152/ajpendo.00473.2017.
36. Vegeto E, Belcredito S, Etteri S, Ghisletti S, Brusadelli A, Meda C, Krust A, Dupont S, Ciana P, Chambon P, Maggi A. Estrogen receptor-alpha mediates the brain antiinflammatory activity of estradiol. Proc Natl Acad Sci U S A. 2003 Aug 5;100(16):9614-9. doi: 10.1073/pnas.1531957100.

2. This is also important as estrogens are known to modulate activation of microglia in dose dependent manner (Vegeto E, et al., PNAS 2003) and it also regulates the activity of enzymes involved in the fatty acid synthesis and regulating brain fatty acid levels (as reviewed in: Morselli E, et al., Am J Physiol Endocrinol Metab.. 2018). Some of the observed changes in transcriptional profile of microglia are likely due to E2 levels in young females than age-associated. The authors need to expand on this and at least validate, if microglial DEGs are indeed age-associated using ovariectomized young females or in a cohort of male mice.

Indeed, we agree that estrogens can affect microglia activity and metabolism. We added the reference of Morselli et al. (Ref. 35) and Vegeto et al. (Ref. 36) in the text (see response to comment 1). However, we believe that old age does also impact inflammation and fatty acid metabolism. The intention of our study was not to compare the response of male and female mice. However, to further address this question, we conducted a validation RNAseq study of Bodipy^{+} microglia obtained from old (n=3) and young (n=4) male mice 4 days post-ischemia to find out whether main pathways upregulated in ischemic microglia of old vs. young female mice were also upregulated in old vs. young male mice. The results confirmed it by showing upregulation in old male mice of main pathways representative of our previous findings in old females, such as: *Response to interferon-beta* (GO:0035456), *Response to interferon-gamma* (GO:003434), *Response to virus* (GO:0009615), *Response to bacterium* (GO:0009617), *Antigen binding* (GO:0003823), and *Long chain fatty acid binding* (GO:0036041), to mention but a few (Fig. EV2, Dataset EV2). Therefore, these findings show that old age induces transcriptional changes in microglia after ischemia involving type-I interferon responses and fatty acid metabolism in both sexes. Further studies are needed to investigate in detail the transcriptional differences in microglia of ischemic mice attributable to sex.

We added the following text in page 5:

These results showing altered inflammatory and metabolic pathways in old versus young ischemic mice were obtained in female mice. Given that estrogens can affect microglia function and brain lipid

metabolism[35,36], we confirmed that many of the above-described pathways were also upregulated in microglia of old versus young male mice after ischemia (Fig. EV2, Dataset EV2).

Figure EV2

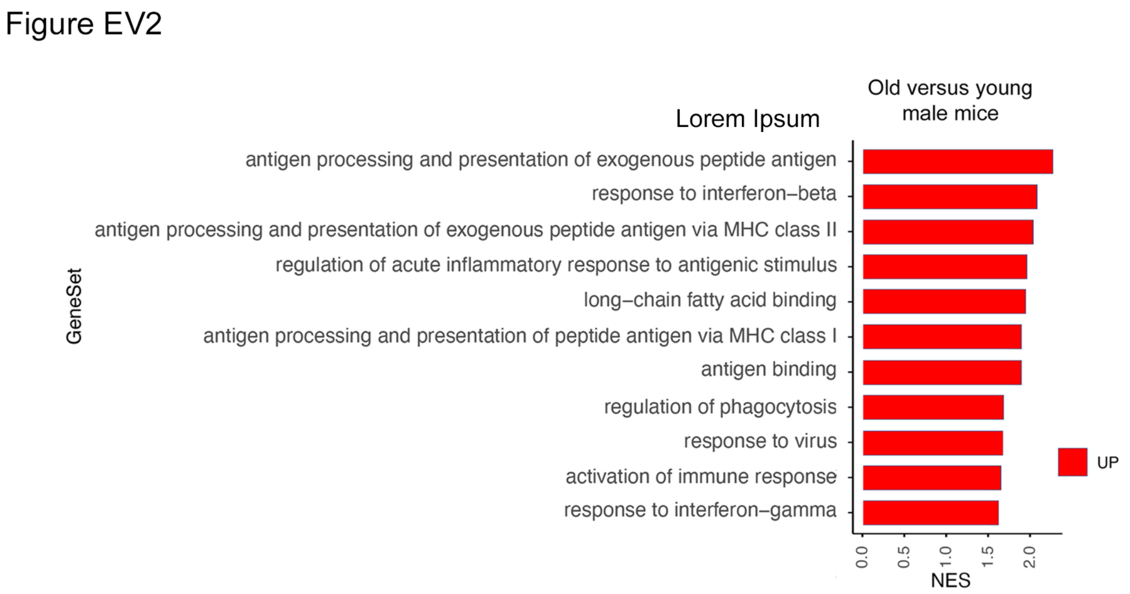

Fig. EV2. Enrichment of genes in GO pathways involved responses in microglia of old vs. young ischemic male mice. *Related to Fig. 3. A transcriptomic analysis (GSE209732) was performed to validate that the main pathways found upregulated in old female mice were also upregulated in old male mice four days after ischemia. GO pathways following RNAseq analysis of microglia (Bodipy$^+$) obtained by FACS from the brain of old (n=3) versus young (n=4) male mice four days after ischemia.*

In addition, we now show the presence of lipid droplets in microglia of the ischemic brain of young and old mice, both male and female, with higher values in old mice than young mice. The results are presented in Fig. 3F for both sexes (pink is female):

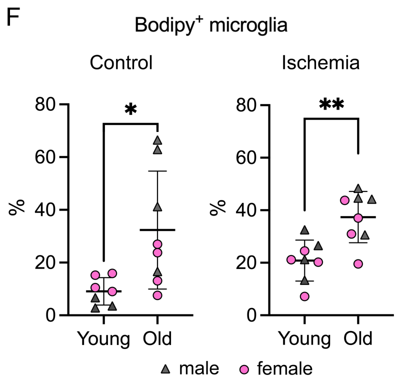

*Fig. 3F) Microglia obtained from young and old male (grey) and female (pink) mice 4 days post-ischemia (ipsilateral hemisphere) and control (contralateral) was studied by flow cytometry (n=7-8 mice per group). Old mice showed higher proportion of microglia containing lipid droplets in control (*p=0.0193) and ischemia (**p=0.0022) (t-test). Values show the mean±SD.*

Moreover, we assessed by electron microscopy the presence of lipid droplets in old male mice. We detected microglia with large lipid droplets in old male mice, whereas old male mice with renewed microglia showed no or small lipid droplets. We added the results in Fig. 5F:

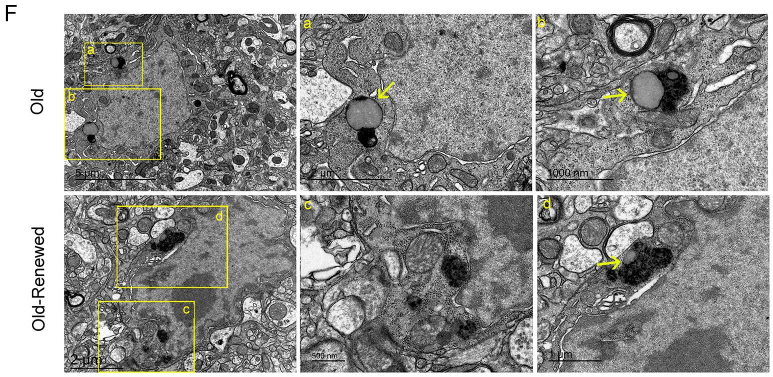

Fig. 5F) Electron microscopy images representative of microglia of old male mice with original microglia (Old) and renewed microglia (Old-Renewed) (n=3 per group). Images labelled (a-d) are higher magnifications of the areas marked with yellow squares in the images on the left. Lipid droplets are marked with arrows. Large lipid droplets are seen in microglia of old mice, whereas renewed microglia typically show no or small lipid droplets.

Minor comments:

1. Provide quantification data for Fig 2B (western blot)

The Western blot has been quantified, as shown in Fig. 2C. In addition, we added an immunofluorescence image showing the presence of PLIN2 in microglia after ischemia (Fig. 2D):

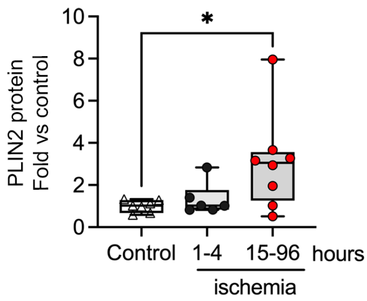

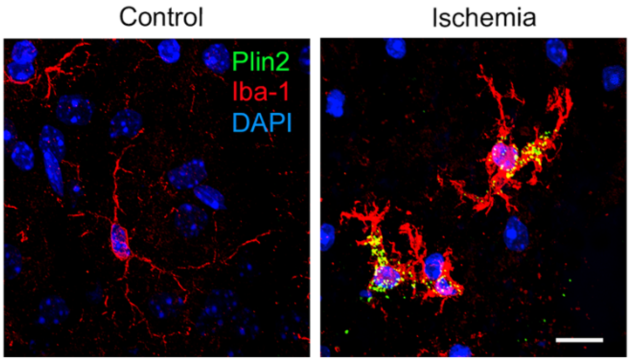

*Fig. 2C) Quantification of band intensity corrected for the loading protein control. Values are expressed as fold versus the control group (non-ischemic). Points correspond to independent male mice and group values are expressed as box and whiskers. For presentation and statistical analysis, we pooled together the values obtained at 1h (n=3) and 4h (n=3) postischemia, and the values obtained at 15h (n=2), 24h (n=3) and 96h (n=3) postischemia. Comparison with the control non-ischemic group showed increase in PLIN2 expression in the time range 15h-4 days post-ischemia *p=0.023, Kruskal-Wallis test and Dunn's multiple comparisons test.*

Fig. 2D) Immunofluorescence with antibodies against Plin2 (green) and Iba-1 (red) in brain tissue 1 day after induction of ischemia in female mice. Nuclei are stained with DAPI (blue). Control is the contralateral hemisphere. Scale bar: 10 μm.

2. Blinding for histological analysis is not mentioned.

Mice received a code that did not reveal the identity of the groups and the analyses were carried out in a blinded fashion. The following sentence was added to page 18:

'At different time points, we euthanized the mice and obtained the brain, which received a code that did not reveal the identity of the experimental group'.

3. Main stroke outcomes data shows, no difference in infarct and modest functional recovery at day 14 (much later compared to lipid droplet accumulation after stroke). Title needs to be revised appropriately as provided data do not fully support the claim, particularly claiming causality where there is only association.

Given that the group of old mice used for behavior up to day 14 post-ischemia was rather small we decided to improve the robustness of the finding by increasing the n value to limit type I errors, as explained in our response to comment n. 1. We recalculated our effect size and the final n values we achieved were n=16 in the old-PLX5622 diet group (renewed group) and n=15 in the old-control diet group. For the neuroscore, there were no differences between groups during the first few days after stroke, but the time course evolution was more favorable in the microglia renewed group. To illustrate this effect, we calculated the ratio of the neuroscore at each time point divided by the neuroscore value at day 1. Values lower than 1 correspond to amelioration of the neuroscore versus day 1. The microglia renewed group improved significantly at day 14 versus day 4 (*** p<0.001, Kruskal-Wallis test), whereas time course differences in the group receiving control diet were not statistically significant (Fig. 6B).

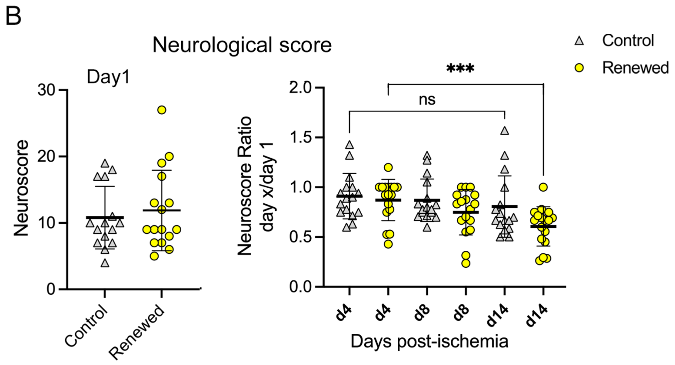

Regarding behavioral tests, the result previously showing improved grip strength at day 14 in the renewed group is confirmed in the larger group of mice. For the rotarod test, differences were not statistically significant before and this result is maintained after increasing the number of mice per group.

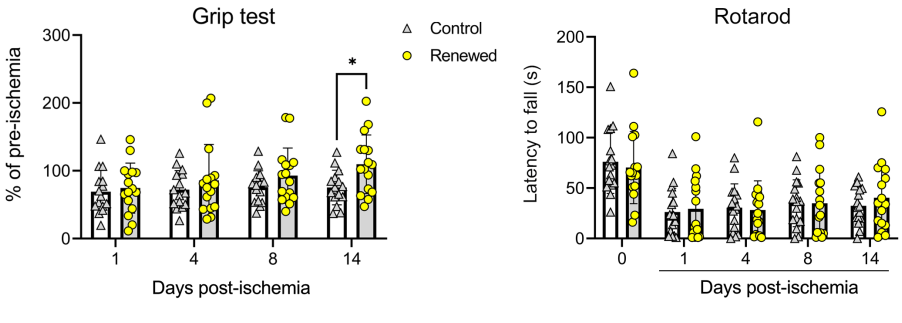

We believe our data support that there is some functional improvement in the old mice after microglia repopulation. Renewal of old microglia is not neuroprotective in the acute phase, but our results show that it contributes to some functional recovery within the two weeks after stroke. Improvement is modest but not negligible. Our point is that microglia in old individuals is not as fit as

in young individuals, and renewing old microglia has some beneficial effect, since likely the cells contribute to better restoration of tissue homeostasis during the days that follow stroke onset.

The above results are presented in figure 6 and described in the corresponding legend and in the main text (page 8, before the Discussion). We added the following text to refine our interpretation of these results:

'*Overall, microglia renewal in old mice prior to ischemia did not reduce the size of the lesion, nor did it improve the neurological score in the first few days postischemia. However, old mice with renewed microglia showed signs of improvement of the neurological function two weeks after stroke suggesting that renewed microglia facilitated recovery of function in old mice'.*

In view of the comments of the reviewer, we decided to change the title to:

'Aged lipid droplet-rich microglia impair neurological recovery after stroke'

4. Please also perform cresyl violet/nissl staining to complement MRI quantification data from 3B and 6C. Is % infarct volume presented on top panel represents analysis of day 4/14?

The mentioned infarct volume measurements correspond to day 4 post-ischemia. This information is provided in the figure legends. We cannot perform the cresyl violet/nissl staining of the corresponding MRI quantifications since the tissue of previous experiments was used for other purposes (RNA, Western blot, flow cytometry). In a previous study (Perez-de-Puig et al., 2013), we showed a correlation between MRI infarct volume and cresyl violet infarct volume in a model of permanent ischemia:

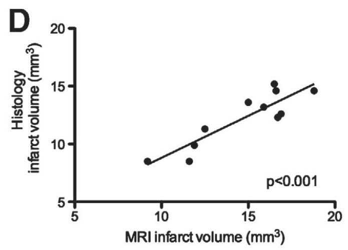

<u>Reference:</u>

Pérez-de Puig I, Miró F, Salas-Perdomo A, Bonfill-Teixidor E, Ferrer-Ferrer M, Márquez-Kisinousky L, Planas AM. IL-10 deficiency exacerbates the brain inflammatory response to permanent ischemia without preventing resolution of the lesion. *J Cereb Blood Flow Metab*. 2013 Dec;33(12):1955-66. doi: 10.1038/jcbfm.2013.155.

Referee #3 (Remarks for Author):

This study examined if microglial renewal can restore+ microglia function and reverse post-stroke neurological deficits. The outcome measures the authors employed for microglial function was primarily transcriptome based, along with in vitro phagocytosis assay and intracellular lipid phenotype assessment. In general, the manuscript was well written and appropriately presented, albeit some fonts within figures were too small. However, there are some concerns that need to be addressed prior to reaching the proposed conclusion:

1) The manuscript is structured to show the effect of stroke on young, then in aged, then aged vs

renewed. Although this enables fluidity in readership, the way that the data presented does not allow for examining the combined impact of aging and stroke. For instance, lipids are abundantly present already in aged microglia (Fig. 3F control), do these cells have heightened inflammatory transcriptomic signature compared to young, and further exacerbated after stroke?

Our study focused on stroke in young and old mice since other works have addressed differences between naïve young and old mice. After stroke, old mice show an exacerbated inflammatory response. Microglia of ischemic old mice show strong upregulation of inflammatory pathways, see for instance in Fig. 3B; notably the response to IFN-β (Fig. 3C) and IFN-γ, as well as other innate immune responses common to infections (shown in Extended View figure EV1), and interleukin-1 production (GO:0032611) (Dataset EV1). Therefore, the ischemia-induced activation of innate immune and inflammatory responses already seen in the young mice is further exacerbated in old mice.

2) The causality of greater intracellular lipids --> type 1 IFN gene signature --> lack of phagocytosis --> worse stroke outcome is not evident. Currently, the data is associative. The discrepancy of this proposed sequence of the events is highlighted in the setting of young, which needs lipid generation for appropriate innate immune response and phagocytosis. Therefore, it is unclear what new information the findings of this manuscript has revealed. What is the mechanism of elevated lipids inducing type 1 IFN signature in aged after stroke?

We thank the reviewer for this important question. We detected lipid accumulation and higher IFN signatures in microglia of old mice. The fact that removing lipid-rich microglia in old mice causes some improvement of the neurological function within the first two weeks after stroke demonstrates that 'old microglia' impairs spontaneous recovery of the neurological function. The question formulated by the Reviewer regarding the mechanism linking elevated lipids and type I IFN signature after stroke is very interesting. We decided to address it using Stat1$^{-/-}$ mice and corresponding Stat1$^{+/+}$ mice. The signal transducer and activator of transcription Stat1 is an important transcription factor mediating IFN signaling (Van Boxel-Dezaire et al., 2006). Given that lipid droplets are decorated with anti-microbial proteins, in particular proteins of the IFN pathway, we hypothesized that the IFN response could be necessary for ischemia-induced lipid droplet biogenesis. According to it, we expected that microglia of Stat1$^{-/-}$ mice would show a lower capacity to generate lipid droplets after stroke. Four days after induction of ischemia we analyzed lipid droplets in microglia by flow cytometry. The results showed ischemia-induced increases in Bopipy^{+} microglia in Stat1$^{+/+}$ mice (** p=0.0086) but not in Stat1$^{-/-}$ mice (p=0.927) (Two-way ANOVA and Šídák's multiple comparisons test). The results are now shown in Fig. 2G, described in the figure legend, and in page 4 of the main text.

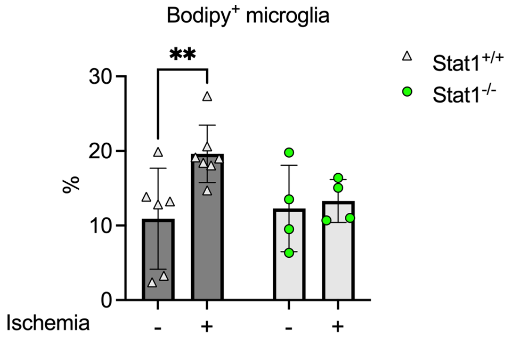

Therefore, this finding provides evidence supporting the involvement of the IFN response in lipid droplet biogenesis in microglia after stroke. We added the following text (page 4):

'The percentage of Bodipy⁺ CD45^{low}CD11b⁺ microglia increased four days post-ischemia in Stat1$^{+/+}$ mice, but not in Stat1$^{-/-}$ mice (Fig. 2G). Stat1 is a critical factor mediating the transduction of cellular responses to several types of IFNs[31]. Therefore, these results show that the IFN response is involved in ischemia-induced lipid droplet biogenesis in microglia.'

In the Discussion (page 9), we added:

'Moreover, we found that IFN signaling is involved in ischemia-induced lipid droplet biogenesis since it is abrogated in Stat1 knockout mice defective in IFN signal transduction.'

Reference:
Ref. 31. Van Boxel-Dezaire AH, Rani MR, Stark GR. Complex modulation of cell type-specific signaling in response to type I interferons. Immunity. 2006 Sep;25(3):361-72. doi: 10.1016/j.immuni.2006.08.014.

3) Many occasions throughout the manuscript the authors mentioned "subset of microglia" - what subset is this? Similarly, if microglial repopulation is dependent on PLX5622-resistant microglia, do these microglia have less lipids thus allowing for the renew population to also have lower lipid content? What makes them resistant to PLX5622? With the greater knowledge of different microglia subsets nowadays, and how they can be identified, delineation of the remaining microglia involved in the renewal process should be achievable.

We used the word 'subsets' to name subgroups, for instance the fraction of microglia that accumulates lipid droplets is the Bodipy$^+$ subset in the flow cytometry studies. To avoid confusion, we tried to minimize the use of this term through the text. We hope this terminology is now clear and acceptable. The question of whether the microglia remaining in the tissue after PLX5622 treatment (with no repopulation) have differential features versus total microglia is very interesting. However, the number of microglial cells recovered after continuous PLX5622 diet is low, and we did not really aim to characterize these cells in this study. We rather aimed to compare the repopulated cells with the original microglia in brain ischemia. We agree that it will be important to understand whether the residual microglial cells remaining after PLX5622 treatment have any specific feature conferring them resistance to CSF1R inhibition, or a fraction of cells remains as the result of a stochastic process. In our view, a separate study will be needed to adequately answer this question.

4) It is interesting that the authors put a lot of emphasis on phagocytosis as a microglial function (the only one they assessed functionally), but microglia has other important functions too especially with their interactions with neurons and neurovascular units. These were not assessed at all, what is rationale behind this? What is the effect of aging and repopulation/renew on these functions?

We fully agree with this comment, and we certainly believe there should be effects of renewed microglia on the function of other brain cells. We carried out a detailed examination of the genes that are differentially expressed in microglia of old versus young ischemic mice that show restoration of expression after microglia renewal. Beyond type I IFN-regulated genes, we identified some more genes in Fig. 4D that can shed some light on this question. Microglia of old mice showed downregulation of *Igf1* mRNA, encoding Insulin growth factor 1, whereas *Igf1* mRNA expression increases in renewed microglia. IGF1 plays important trophic functions in the CNS and administration of IGF1 has shown beneficial effects in experimental stroke models (ref. 49). A similar pattern of gene expression is observed for: amyloid precursor-like protein 1 (*Aplp1*), which physiological function is not completely understood but could be involved in synaptic plasticity; the

matrix protein Matrylin-3 (*Matn3*); *CD59a*, an inhibitor of complement membrane attack complex and inflammasome activation; Collectin-12 (*Colec12*), a member of the C-lectin family that could be involved in cleaving Amyloid-beta; and Melanoma Cell Adhesion Molecule (*Mcam*) that interacts with vascular VEGFR-2. The above genes encoding microglia-derived molecules are likely to exert beneficial effects in other neural cells, such as neurons and endothelium. In contrast, examples of genes upregulated in microglia of old mice, which effect was reversed by microglia renewal,include: the chemokine *Ccl8* that we previously showed is produced by microglia (ref. 26); *CD209b*, a pattern recognition C-type lectin; and *Fcnb,* Ficolin-b (Ficolin-1 in humans) is a pattern recognition receptor involved in activating the lectin pathway of the complement system. These latter genes could exacerbate the inflammatory response and enhance secondary brain damage in old mice. Therefore, attenuation of expression in mice with renewed microglia could be beneficial. We added a brief text in results (page 6):

'A pattern of gene expression similar to that of Igf1 was observed for amyloid precursor-like protein 1 (Aplp1), matrix protein Matrylin-3 (Matn3), CD59a, Collectin-12 (Colec12), and Melanoma Cell Adhesion Molecule (Mcam).'

In the Discussion, page 8, we included this text:

'Additional features of microglia of old mice that were restored after microglia repopulation could contribute to improve neurological recovery after stroke. For instance, relative to young mice, microglia of old mice showed downregulation of Igf1 mRNA after ischemia, whereas microglia renewal in old mice upregulated the expression of Igf1. IGF1 plays important trophic and neurogenic functions in the CNS[36]. Moreover, administration of IGF1 has shown beneficial effects in experimental stroke models[49].'

References:

Ref 26: Gallizioli M, Miró-Mur F, Otxoa-de-Amezaga A, Cugota R, Salas-Perdomo A, Justicia C, Brait VH, Ruiz-Jaén F, Arbaizar-Rovirosa M, Pedragosa J, Bonfill-Teixidor E, Gelderblom M, Magnus T, Cano E, Del Fresno C, Sancho D, Planas AM. Dendritic Cells and Microglia Have Non-redundant Functions in the Inflamed Brain with Protective Effects of Type 1 cDCs. Cell Rep. 2020 Oct 20;33(3):108291. doi: 10.1016/j.celrep.2020.108291.

Ref. 49. Lioutas VA, Alfaro-Martinez F, Bedoya F, Chung CC, Pimentel DA, Novak V. Intranasal Insulin and Insulin-Like Growth Factor 1 as Neuroprotectants in Acute Ischemic Stroke. Transl Stroke Res. 2015 Aug;6(4):264-75.

5) Whilst transcriptomic assessment is useful and obviously "very hot" in recent years - what is the implication of microglia renewal in the general brain health (although infarct size is not changed) - ie. astrocytic activation and scarring? Cerebral inflammatory environment? Neuronal rewiring?

Regarding the inflammatory environment, which is exacerbated in aged mice, we expected it to be reduced after microglia renewal, according to the reduction of pro-inflammatory gene expression pathways in renewed microglia. Microglial cells interact with many different cells, notably neurons, astrocytes and the vascular endothelium. In line with the response to the previous question, we noticed the effect of microglia renewal in old mice restoring *Igf1* expression, which is expected to impact synaptic function and promote neurogenesis. Studying the expression of synaptic proteins as well as different populations of neurons, and neurogenesis under the various experimental conditions could provide some clues to this question. In addition, it would be interesting to study the response of the endothelium to microglia renewal after ischemia, the function of the blood-brain barrier and the process of angiogenesis, and also possible differences in the formation of the glial scar. However, we believe all these studies are out of the scope of this manuscript.

6) Improvement of stroke outcome after microglial repopulation is arguable - only Neuroscore really showed improvement and this assessment is not fully dependent on neurological functions but also general health. It bets the question whether the effect seen in this study is not specific to microglia, but rather involvement of peripheral CSF1R-dependent cells too - doi.org/10.1073/pnas.1922788117.

We cannot exclude that the effects of treatment on peripheral immune cells, as shown by Lei et al., 2020 (ref. 50), could have some contribution to stroke outcome. However, in old mice we expect a higher impact of cell renewal in microglia since these cells are long living cells that accumulate age-dependent dysfunctions compared to peripheral cells, which have a shorter half-life. Nonetheless, we believe it is important to mention the possibility of contributing effects of peripheral cells. Accordingly, we added the following sentence in the Discussion (page 10):
'While microglia renewal in old mice is likely a critical player in the observed benefits of PLX5622 treatment for stroke outcome, some contribution of changes induced by treatment in peripheral immune cells cannot be excluded[50].'

<u>Reference:</u>

Ref. 50: Lei F, Cui N, Zhou C, Chodosh J, Vavvas DG, Paschalis EI. CSF1R inhibition by a small-molecule inhibitor is not microglia specific; affecting hematopoiesis and the function of macrophages. Proc Natl Acad Sci U S A. 2020 Sep 22;117(38):23336-23338. doi: 10.1073/pnas.1922788117.

7) Does microglia repopulation occur in the stroke infarct or surrounding regions after stroke? If so, do these repopulated cells demonstrate renewed phenotypes observed?

We found repopulated microglia in the non-ischemic and ischemic hemispheres as assessed by immunofluorescence with an antibody against P2YR12. After brain ischemia, microglial morphology changes acquiring larger body size and thicker ramifications at the periphery of infarction whereas in the core of the lesion microglia cell number and P2RY12 immunoreactivity decrease, as reported (ref. 18). The ischemic microglial reaction and morphology appeared to be similar after microglia repopulation. This distribution pattern four days post-ischemia is illustrated in Extended View Fig. EV3 showing representative images of the striatum of a mouse brain with original microglia and a mouse with renewed microglia. We added the following text in page 5:
'We examined microglia repopulation by switching to control diet for 7 days prior to ischemia and studying the brain 4 days postischemia. Microglia repopulated the contralateral and ipsilateral hemispheres. Renewed microglia showed ramified morphology like original microglia, and the cells acquired reactive morphology at the periphery of infarction and in the lesion core resembling the morphology displayed by the original microglia in these locations (Fig. EV3).'

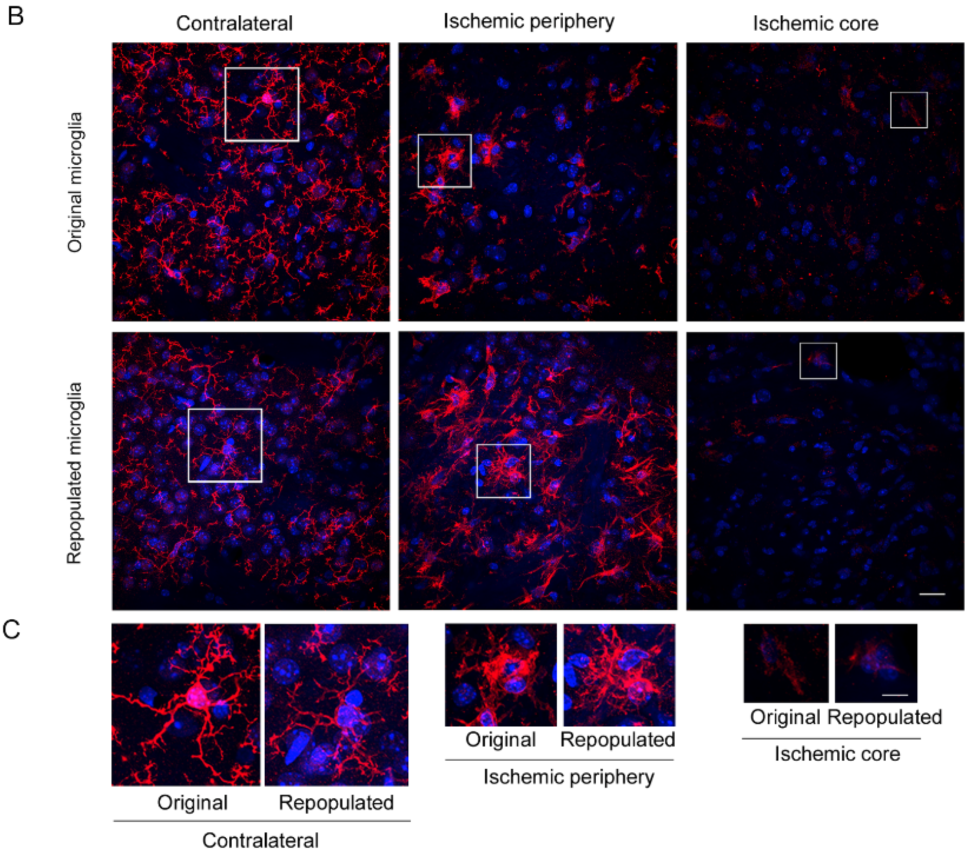

***Fig. EV3**: A) Representative images of the striatum of young female mice with original microglial and repopulated microglia 4 days post-ischemia (n=3 per group). Images show microglial cells immunostained with anti-P2YR12 (red) in the non-injured contralateral hemisphere, the periphery of ischemia, and the lesion core. Cell nuclei are labelled with DAPI (blue). After repopulation, microglia react to ischemia acquiring a reactive morphology that appears to be similar than that of the original microglia. C) Magnification of individual cells marked with squares in D. Scale bar: (B) 20 µm, (C) 10 µm.*

8) What is the clinical relevance of the findings?

Microglia are long living cells and several lines of evidence support that their function is impaired in old age. Microglia of aged individuals can accumulate insoluble materials in the lysosomes and lipids, likely due to dysfunctional lipid disposal, while displaying an exacerbated inflammatory reaction to brain ischemia. We also showed that brain ischemia, as inflammation and infection, induces metabolic changes in microglia involving acute lipid droplet biogenesis that is dependent, at least in part, on deployment of the type I interferon immune program. The presence of lipid droplets already in a fraction of microglia of old mice under steady state impairs novel lipid droplet biosynthesis and the metabolic adaptation necessary to respond to ischemia. We showed that renewing the microglia population of old mice restored some of the responses of old microglia to brain ischemia and ameliorated the functional recovery of the mice. Therefore, microglia are putative targets to improve the fitness of the old brain. Lipids could be druggable targets in microglia to increase the cellular fitness in old subjects. The possibility that targeting lipids after stroke could improve recovery in the long term by improving the fitness of microglia should also be explored.

****

Additional comments from Referee #1:

"I very much agree with Referee #3 comments. If the authors can carefully revise manuscript as per review comments, it would substantially improve the manuscript and would make it more suitable for publication in EMBO Mol Med.

Here are few experiments that the authors can perform to improve the study:
1. Lack of neuroprotection in young females (Fig 3A) compared to aged needs more justification. Authors should provide estrogen levels data from plasma/serum samples of these young and aged mice.

As we mentioned above in our reply to the initial comment 1, we believe that our initial sample size was too small. Now, after correcting for it, the mean infarct volume is lower in young than old female (please see response to comment 1 of Referee 1). In view of the request of the reviewer, we carried out an ELISA assay of 17β-estradiol in plasma of other sets of young and old control female mice. We found high variability in the young group, which clearly showed some mice with the highest values and other mice with low values, as follows:

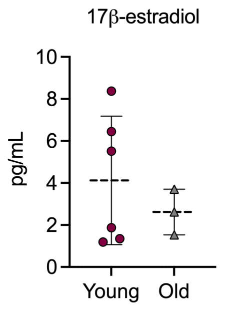

Appendix Fig. S1: 17β-estradiol concentration in plasma of young (n=6) and old (n=3) female mice show large coefficient of variation (74%), likely attributable to different phases of the estrus cycle.

We added this sentence in the Results (page 4):

'Given the protective effect of estrogens[32], it is possible that differences in plasma estrogen levels in individual young female mice (Appendix Fig. S1), attributable to the various stages of the estrus cycle, contributed to infarct volume variability in young female mice.'

Reference

32. Koellhoffer EC, McCullough LD. The effects of estrogen in ischemic stroke. Transl Stroke Res. 2013 Aug;4(4):390-401. doi: 10.1007/s12975-012-0230-5.

2. Some of the observed changes in transcriptional profile of microglia (Fig 3-5) are likely due to E2 levels in young females than age-associated. The authors need to expand on this and at least validate, if microglial DEGs are indeed age-associated using ovariectomized young females or in a cohort of male mice.

Although this study was not intended to compare sex differences, we wanted to find out whether the main pathways involved in the described responses were also upregulated in old versus young male mice, hence we studied DEGs as explained above in response to comment 2 of Referee 1. We decided to conduct a validation RNAseq study of microglia (Bodipy^{+}) obtained from old (n=3) and

young (n=4) male mice 4 days post-ischemia to find out whether main pathways upregulated in ischemic microglia of old vs. young female mice were also upregulated in old vs. young male mice. The results confirmed this by showing upregulation in old male mice of the main pathways highlighted in our previous findings in old females, such as: *Response to interferon-beta* (GO:0035456), *Response to interferon-gamma* (GO:003434), *Response to virus* (GO:0009615), *Response to bacterium* (GO:0009617), *Antigen binding* (GO:0003823), and *Long chain fatty acid binding* (GO:0036041), to mention but a few (Fig. EV2, Dataset EV2). Therefore, these findings show that old age induces transcriptional changes in microglia after ischemia involving type-I interferon responses and fatty acid metabolism in both sexes. Further studies are needed to investigate in detail the transcriptional differences in microglia of ischemic mice attributable to sex.

Figure EV2

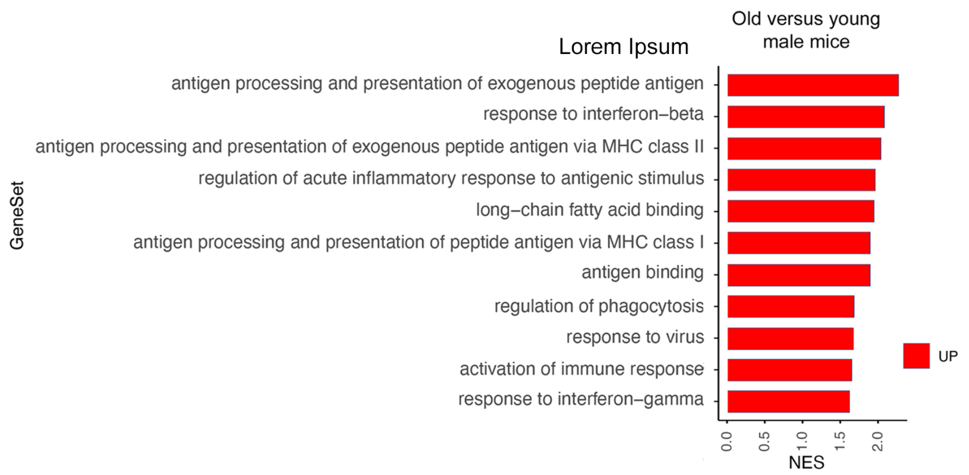

Fig. EV2. Enrichment of genes in GO pathways involved responses in microglia of old vs. young ischemic male mice. *Related to Fig. 3. A transcriptomic analysis (GSE209732) was performed to validate that the main pathways found upregulated in old female mice were also upregulated in old male mice four days after ischemia. GO pathways following RNAseq analysis of microglia (Bodipy^{+}) obtained by FACS from the brain of old (n=3) versus young (n=4) male mice four days after ischemia.*

.

3. Authors should consider testing post-stroke recovery in more sensitive and/or chronic behavioral tests for 30d or longer to test for beneficial effects of renewed microglia. Provided data from behavioral tasks is a major weakness and suggests no major clinical relevance from renewed microglia

As explained above answering question 3 (minor comments) of Referee 1, we increased the number of mice in the behavioral tests since we believed this could augment the robustness of the results. There is a consistent amelioration of the grip strength in the old mice with renewed microglia versus old mice with original microglia. We also detected some amelioration of the neuroscore in the group of old mice with renewed microglia. We agree that the beneficial effects are not dramatic and they do not change the lesion size, but the small recovery of function should not be considered negligible. The results support the concept that microglia of old mice become dysfunctional compared with that of young mice, and old microglia is less fit to cope with brain insults such as stroke. Moreover, preventing lipid accumulation, together with other features of dysfunction, in microglia of aging individuals could be a target for therapeutic intervention to improve healthy aging and to minimize the neurological impact of age-related diseases such as stroke.

4. Clinical relevance would improve if findings from Fig 2C and/or 3G were validated using patient samples.

Indeed, experimental findings should be validated in human samples, which will require a full study on its own. At this stage we could only study mRNA obtained from the brain of nine patients who died at 1, 3, 4, 5, 5, 6, 18 or 140 days after stroke onset at the Stroke Unit of the Hospital Clinic of Barcelona. Written consent was obtained from the families for tissue removal after death for diagnostic and research purposes at the Neurological Tissue Bank of the Biobank-Hospital Clinic-Institut d'Investigacions Biomèdiques August Pi i Sunyer (IDIBAPS). The study had the approval of the Ethics Committee of this Hospital. Basic characteristics of the patients are shown in Table EV1. The result showed that the mRNA expression of *Plin2* and *Isg15*, one of the type-I IFN-regulated genes (see Fig. 2A), increases in the human ischemic tissue versus non-affected (NA) tissue, suggesting that the mechanisms involved in ischemia-induced lipid droplet biogenesis are also activated in human stroke (Fig. 2B).

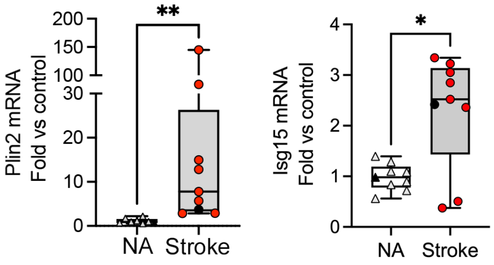

*Fig. 2B. mRNA expression of Plin2 and Isg15 in post-mortem human brain tissue of 9 stroke patients (points are values of individual patients), 8 women (red points) and 1 man (black point). Samples were obtained from the ischemic tissue and non-affected tissue (NA). Values are expressed as fold versus mean control (non-affected tissue). Wilcoxon matched-pairs signed rank test, **p=0.0039; *p=0.0195.*

We added the following text in page 3-4:

We investigated whether this response was also upregulated after human stroke using post-mortem brain tissue. Characteristics of the stroke patients are shown in Table EV1. Stroke increased the mRNA expression of Plin2 and Isg15, one of the typical type-I IFN responsive genes, suggesting that the molecular machinery related to lipid droplet biogenesis was activated after stroke in the human brain too (Fig. 2B).

17th Aug 2022

Thank you for sending us your revised manuscript. We have now heard back from the two referees who agreed to evaluate your study. As you will see below, the referees raise substantial concerns about your work, which unfortunately preclude its publication in EMBO Molecular Medicine.

The referees acknowledge the effort that has been made to revise the manuscript. However, Referee #3 raises significant concerns about several issues that were already brought up during the first round of review, such as the age effects and neurological outcomes, and mentioned additional technical problems. During our pre-decision cross-commenting process (in which the referees are given a chance to make additional comments, including on each other's reports), Referee #1, who was initially supportive in their report, thought that Referee #3's concerns were valid. I have included below the referee's comments during cross-commenting.

Based on the referees' comments, we think these concerns seem to be substantial and remain insufficiently addressed. Considering that thoroughly addressing the referees' concerns would involve substantial further experiments with an unclear outcome, in combination with the fact that our editorial policy allows, in principle, a single round of major revisions, we see no choice but to return the manuscript with the message that we cannot offer to publish it.

Nevertheless, as the reviewers did acknowledge the potential interest of the study, we would not be opposed to considering a substantially revised manuscript based on this work, provided that the issues raised by the reviewers can be convincingly addressed.

The reviewers provide constructive suggestions on how to address these issues and improve the study (see referee cross-commenting). We realize that this requires a significant investment and may prove challenging. We understand if in light of the substantial revisions required, you prefer to submit your study elsewhere.

A resubmitted will be editorially evaluated afresh, and its novelty will be re-assessed at the time of submission. As you probably understand, we can give no guarantee about its eventual acceptability. If you do decide to follow this course, then we would ask you to enclose with your re-submission a point-by-point response to the points raised in the present review.

I am sorry that the review of your work did not result in a more favorable outcome on this occasion, but I hope that you will not be discouraged from sending your work to EMBO Molecular Medicine in the future.

Kind regards,
Jingyi

Jingyi Hou
Editor
EMBO Molecular Medicine

***** Reviewer's comments *****

Referee #1 (Comments on Novelty/Model System for Author):

The authors have been extremely responsive to the previous concerns, as a result, I think this manuscript is now significantly improved.

Depletion and restoration of microglia may have some severe limitations in clinical translation, but it is an interesting tool to study role of microglia using preclinical models.

Referee #1 (Remarks for Author):

The authors have been responsive and addressed all of this reviewer's previous concerns.

Referee #3 (Comments on Novelty/Model System for Author):

This reviewer appreciates the authors thorough responses to the comments, especially with increasing the sample size to 17 for infarct analyses and validating the RNAseq findings in male mice. However, there are new and remaining concerns that will need addressing before publication:

1) The observation of increased Bodipy+ microglia still confirms this is an aged effect, independent of stroke. For instance, the amount of lipid droplets in microglia of aged mice is comparable between control vs stroke.

2) Closer examination of the methods reveal the "control" group is actually the contralateral hemisphere of a post-stroke animal, instead of a separate cohort of sham-operated animal. As there are increasing literature to suggest the involvement of cells in the contralateral hemisphere in the neural recovery phases post-injury, it begs the question of whether the experimental control is appropriate. Moreover, the text of the manuscript reporting "after stroke" is not valid, it should read "in the ischemic hemisphere" to be accurate.

3) There is an indication of sample/timepoint pooling for Western quantification, however this is not a scientific rationale. The lack of n per timepoint does not justify pooling in a manner that is convenient for the authors. Furthermore, the statistical significance seems to be skew by a single data point in the "15-96 hour" group. Without this outlier, the finding is arguably not significant.

4) Even when the authors try to create a neurological outcome measure to encompass "time course evolution" (neuroscore ratio), there is no difference between control vs renewed at d14. Comparing between d4 and d14 in renewed (Fig 6B) does not answer their scientific hypothesis. To this end, the statistic testing of Grip test between the treatment group was correctly performed and this statistical analysis should be adopted consistently.

5) Over-reaching statements such as "signs of improvement of neurological function" should avoided, the findings only demonstrate improvement of grip strength. Similarly, despite title has been changed, this review cannot see "neurological recovery" assessment performed or

supported with the data presented. If lipid droplet-rich microglia impair recovery, what was the evidence of efficient neurological recovery in non-lipid droplet rich microglia?

6) Additional microglial function outcome was not assessed but simply implied with the existing RNAseq data. Even with this, the authors did not perform any validation experiments to support their claims. Moreover, the notion of extrapolating the transcriptomic data to cellular function and interaction with other cell type within the ischemic brain is over-reaching. The question was initially related to how the lipid droplet-rich microglia can impact surrounding more abundant cell types that could explain the lack of neurological improvement. This query was not addressed adequately.

7) The representative images in EV3 revealed the renewed/repopulated microglia demonstrate more reactive morphology (or there are more reactive microglia) in the ischemic periphery than original. There are abundance of image analysis tools available to support the authors claim of similarity but first look at the images do not support the authors' claim.

****

Referee Cross-commenting:

Referee#3
I agree with the Editor that points #5 and #6 can be textually addressed, but in addition to points #1-4, there is also point #7 regarding the (dis)similarity of original vs renewed microglia. I am keen to hear Referee #1 input.

Referee #1
I agree with Referee#3, these are valid concerns that need to be addressed.
As editor & R#3 rightly mentioned, some of these concerns can be addressed by rewording, but others may need additional experiments.

Here are some suggestions to address these concerns:

#1. As age and stroke both increase Bodipy+ microglia numbers and amount of lipid droplets there may be a ceiling effect (if this is the case authors should at least discuss this), but to better understand, authors should test for stroke effect in aged mice using later timepoints (day 7 or day 14?) and by using sham brains as controls instead of contralateral hemisphere.
#2. Contralateral hemisphere of a post-stroke animal is not the best control, authors should use a sham cohort to at least validate key findings by comparing with stroke brains.
#3. Lack of rational for pooling different timepoints is a weakness. Also, as contralateral hemisphere is not an appropriate control (provided data is not sufficient to confirm, if there is an increase of PLIN2 in ipsilateral hemisphere or a decrease in contralateral hemisphere?), this needs to be confirmed using a sham cohort. A more specific experiment would be to validate if this increase of PLIN2 in stroke brains is significant compared to sham (either at day-4 or day-14, based on human data, it appears that PLIN2 levels remain elevated for several days after stroke).
#4. Authors should adopt statistical analysis consistently between behavioral tests. Current analysis is not uniform between tests. Analysis by repeated measures is a more specific statistical test for accessing early recovery.
#7. To better support authors' statements, quantification analysis needs to be performed on IHC images by using image analysis tools or by flow using a separate cohort.

Referee #3

Thank you Referee#1 for your thoughtful response, I completely agree with your assessments and suggestions.

ANSWERS TO REVIEWERS

Referee #1 (Comments on Novelty/Model System for Author):
The authors have been extremely responsive to the previous concerns, as a result, I think this manuscript is now significantly improved.
Depletion and restoration of microglia may have some severe limitations in clinical translation, but it is an interesting tool to study role of microglia using preclinical models.

Referee #1 (Remarks for Author):
The authors have been responsive and addressed all of this reviewer's previous concerns.

Thank you.

Referee #3 (Comments on Novelty/Model System for Author):
This reviewer appreciates the authors thorough responses to the comments, especially with increasing the sample size to 17 for infarct analyses and validating the RNAseq findings in male mice.

Thank you.

However, there are new and remaining concerns that will need addressing before publication:

1) The observation of increased Bodipy$^+$ microglia still confirms this is an aged effect, independent of stroke. For instance, the amount of lipid droplets in microglia of aged mice is comparable between control vs stroke.

Microglia of old mice contain lipid droplets as an age-dependent effect. However, ischemia increases LD-rich microglia also in old mice, but to a lower extent than in young mice. This is better appreciated in the figures arising from the new experiments we did in sham-operated mice, as can be seen in the answer to next question.

2) Closer examination of the methods reveal the "control" group is actually the contralateral hemisphere of a post-stroke animal, instead of a separate cohort of sham-operated animal. As there are increasing literature to suggest the involvement of cells in the contralateral hemisphere in the neural recovery phases post-injury, it begs the question of whether the experimental control is appropriate. Moreover, the text of the manuscript reporting "after stroke" is not valid, it should read "in the ischemic hemisphere" to be accurate.

To address this point, we did an entire new experiment where we studied lipid droplets in microglia of sham-operated young and old mice (male and female), as assessed by Bodipy staining in flow cytometry. The results clearly show more LD-rich microglia in old mice (both male and female) than in young mice, both after sham-operation and after ischemia. Microglial cells containing LDs are negligible in sham-operated young mice of both sexes. Ischemia induced the formation of LDs in microglia of young mice (8.3-fold increase vs. sham) and, to a lower extent, in old mice (2.7-fold increases versus sham), which already showed the presence of lipid droplet-rich microglia under sham conditions. Results are presented in Fig. 3F.

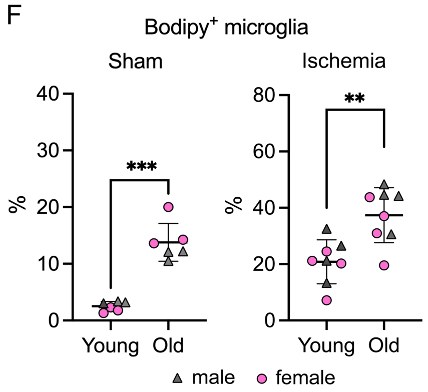

We modified the text in Results as follows (page 5, 3rd paragraph):

'By flow cytometry we found that old male and female mice showed more lipid droplet-rich microglia (Bodipy+) than young mice under sham and ischemic conditions (Fig. 3F). Compared to sham-operation, ischemia increased the percentage of lipid droplet-rich microglia by 8.3-fold in young mice and 2.7-fold in old mice (Fig. 3F).'

Microglia repopulation in old mice (yellow symbols) reduced the % of Bodipy+ microglia found in old mice, both after sham-operation and after ischemia (Fig. 5D).

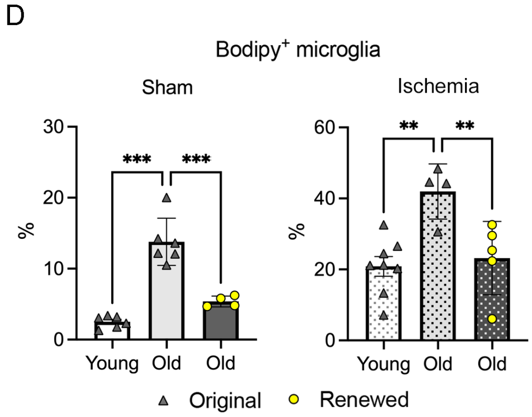

In addition, in a different group of mice we sorted microglia from the ipsilateral brain hemisphere 4 days after sham-operation in young (Y) (n=4) and old (O) mice (n=3) and did a new RNAseq analysis (GSE212056, Token: gvyvckucbdmhdgp). Here we show the principal components analysis (PCA) that clearly distinguished both groups:

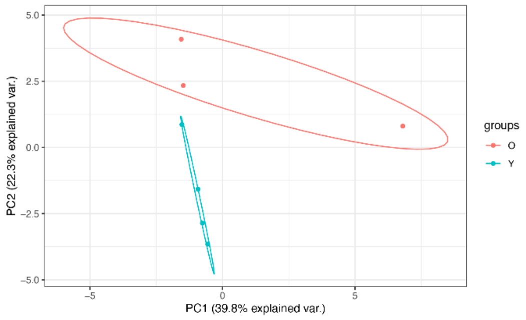

In microglia of old vs. young sham-operated mice, we found upregulation of pathways related to lipid storage and metabolism, together with innate immune and pro-inflammatory pathways. Data are presented in new *Dataset EV2* and we added the following text in page 5:

'For comparative purposes, we also looked for changes in microglia gene expression in old versus young female mice 4 days after sham-operation (Dataset EV2). Microglia of old versus young sham-operated mice showed upregulation of innate immune and pro-inflammatory GO pathways, together with upregulation of GO pathways involved in lipid accumulation and metabolism, such as 'Fatty acid binding', 'Cellular response to fatty acid', 'Triglyceride metabolic process', 'Lipid storage', 'Cholesterol storage', 'Regulation of fatty acid biosynthetic process', 'Lipoprotein particle binding', and 'Long-chain fatty acid binding', amongst others. Therefore, compared to young microglia, microglia of old mice show a pro-inflammatory profile and altered lipid metabolism in the absence of ischemia.'

According to the suggestion, we changed 'after stroke' to 'in the ischemic tissue' through the text.

3) There is an indication of sample/timepoint pooling for Western quantification, however this is not a scientific rationale. The lack of n per timepoint does not justify pooling in a manner that is convenient for the authors. Furthermore, the statistical significance seems to be skew by a single data point in the "15-96 hour" group. Without this outlier, the finding is arguably not significant.

We added more ischemic mice and performed a new group of sham-operated mice. PLIN2 expression shows significant increases at day 4 postischemia vs. sham-operation.

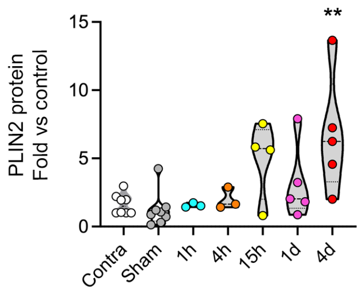

Fig. 2 C) PLIN2 protein expression in mouse brain tissue, as assessed by Western blotting. Values were obtained from the ipsilateral hemisphere 1h (n=3), 4h (n=3), 15h (n=4), 24h (n=5) and 96h (n=5) post-ischemia, and 15h (n=4), 24h (n=2), and 96h (n=2) after sham-operation. Values of sham mice were pooled together since they did not differ between time points. Samples of the contralateral hemisphere (Contra) of ischemic mice (1h, n=2; 24h, n=3; and 96h, n=3) were also evaluated. Ischemia increased PLIN2 expression at day 4 vs. the sham group (**p=0.0032, Kruskal-Wallis test and Dunn's multiple comparisons test).

4) Even when the authors try to create a neurological outcome measure to encompass "time course evolution" (neuroscore ratio), there is no difference between control vs renewed at d14. Comparing between d4 and d14 in renewed (Fig 6B) does not answer their scientific hypothesis. To this end, the statistic testing of Grip test between the treatment group was correctly performed and this statistical analysis should be adopted consistently.

In the former presentation of the neuroscore results we already stated in the text that there were no differences between groups. The intra group variation in the neuroscore values at day one in both groups was very large, without differences between groups. We reasoned that normalizing for each mouse the neuroscore value at the different time points by its own initial neuroscore value at day 1 could help to have a clearer view of the time course evolution while overcoming the high initial variability between subjects. This was the reason for our former presentation of the results. Please notice that we did not try to create any artificial outcome but just normalize data of each mouse to its own initial value, given the large initial variability.

In view of the comments of the Reviewer, we removed the normalized data and now show the raw neuroscore values. The neuroscore value (mean±SD) decreased from day 1 to day 14 by 23% in the control group (from 10.8±4.7 to 8.3±4.2) and by 46% in the microglia renewed group (from 11.9±6.1 to 6.4±2.8). Differences between groups were not statistically significant using the Kruskal-Wallis test with a repeated measure design (Fig. 6B).

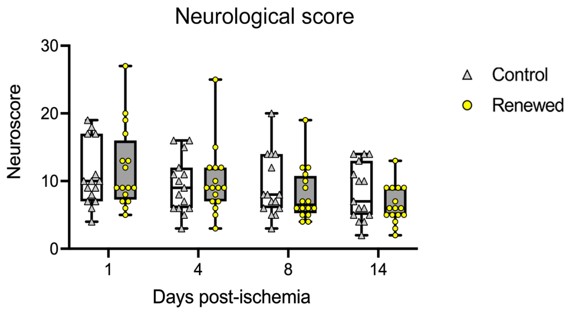

Fig. 6B) The neuroscore value (mean±SD) decreased from day 1 to day 14 by 23% in the control group (from 10.8±4.7 to 8.3±4.2) and by 46% in the microglia renewed group (from 11.9±6.1 to 6.4±2.8). The variability within groups was large and differences between groups were not statistically significant (p=0.21, non-parametric Kruskal-Wallis test with a repeated-measures design). Points are individual values for each mouse, box and whiskers show the median, and bars indicate min. and max. values.

We added this explanation in the text (bottom of pag. 7):
'The mean neuroscore value decreased (improved) from day 1 to day 14 by 23% in the control group, and by 46% in the microglia renewed group. However, the variability within groups was high and differences between groups were not statistically significant (Fig. 6B).'

5) Over-reaching statements such as "signs of improvement of neurological function" should avoided, the findings only demonstrate improvement of grip strength. Similarly, despite title has been changed, this review cannot see "neurological recovery" assessment performed or supported with the data presented. If lipid droplet-rich microglia impair recovery, what was the evidence of efficient neurological recovery in non-lipid droplet rich microglia?

'Signs of improvement of the neurological function' has been changed to 'S*igns of improvement of motor function*', as assessed by the grip test.
Title has been changed to:
'Aged lipid-laden microglia display impaired responses to stroke'.

6) Additional microglial function outcome was not assessed but simply implied with the existing RNAseq data. Even with this, the authors did not perform any validation experiments to support their claims. Moreover, the notion of extrapolating the transcriptomic data to cellular function and interaction with other cell type within the ischemic brain is over-reaching. The question was initially related to how the lipid droplet-rich microglia can impact surrounding more abundant cell types that could explain the lack of neurological improvement. This query was not addressed adequately.

We did not investigate additional cellular functions because this deserves a separate full study. We believe that RNAseq data is useful to guide new hypothesis that then deserve testing in depth in further investigation. Other studies clearly recognized that microglia of old mice accumulating lipids are dysfunctional cells. These studies are cited in the text of the manuscript. See for instance Marschallinger et al. (ref. 11) and Cantuti-Castelvetri et al. (ref. 9).

Marschallinger J, Iram T, Zardeneta M, Lee SE, Lehallier B, Haney MS, Pluvinage JV, Mathur V, Hahn O, Morgens DW, Kim J, Tevini J, Felder TK, Wolinski H, Bertozzi CR, Bassik MC, Aigner L, Wyss-Coray T. **Lipid-droplet-**

accumulating microglia represent a dysfunctional and proinflammatory state in the aging brain. *Nat Neurosci*. 2020 Feb;23(2):194-208. doi: 10.1038/s41593-019-0566-1.

Cantuti-Castelvetri L, Fitzner D, Bosch-Queralt M, Weil MT, Su M, Sen P, Ruhwedel T, Mitkovski M, Trendelenburg G, Lütjohann D, Möbius W, Simons M. **Defective cholesterol clearance limits remyelination in the aged central nervous system.** *Science*. 2018 Feb 9;359(6376):684-688. doi: 10.1126/science.aan4183.

7) The representative images in EV3 revealed the renewed/repopulated microglia demonstrate more reactive morphology (or there are more reactive microglia) in the ischemic periphery than original. There are abundance of image analysis tools available to support the authors claim of similarity but first look at the images do not support the authors' claim.

We agree that the image of a single or few microglial cells, as shown in Fig. EV3, does not allow to extrapolate about the morphology of the whole population, given that microglial cells are highly polymorphic. To properly answer this question, we carried out an entire morphometric analysis of original versus repopulated microglia in brain tissue sections stained with antibodies against P2YR12 four days post-ischemia. We used publicly available plugins for Fiji software enabling cell segmentation and the study of morphological descriptors. We studied microglial cell area, circularity and solidity in microglial cells of n=4 ischemic mice per group. Cells were sampled from three zones within the striatum, i.e. core and periphery in the ipsilateral hemisphere and the contralateral hemisphere. For these respective regions, the total number of cells analyzed were 96, 233, and 91 in the group with original microglia, and 58, 178, and 109 in the group with repopulated microglia, respectively. We did not detect significant differences between groups in the contralateral hemisphere. Both groups showed increased microglia circularity in the ischemic hemisphere versus the contralateral hemisphere, in agreement with the reduced arborization of the cells in the most injured regions. Comparison between groups showed a significant decrease in circularity in the microglial cells located at the periphery and core of infarction in the repopulated microglia groups versus the group with original microglia. We did not detect significant differences in the other parameters and zones. This finding suggests that the decrease in cell branching observed in microglia after ischemia was less pronounced in the repopulated microglia compared with the original microglia. The results are included in Suppl. Fig. EV3.

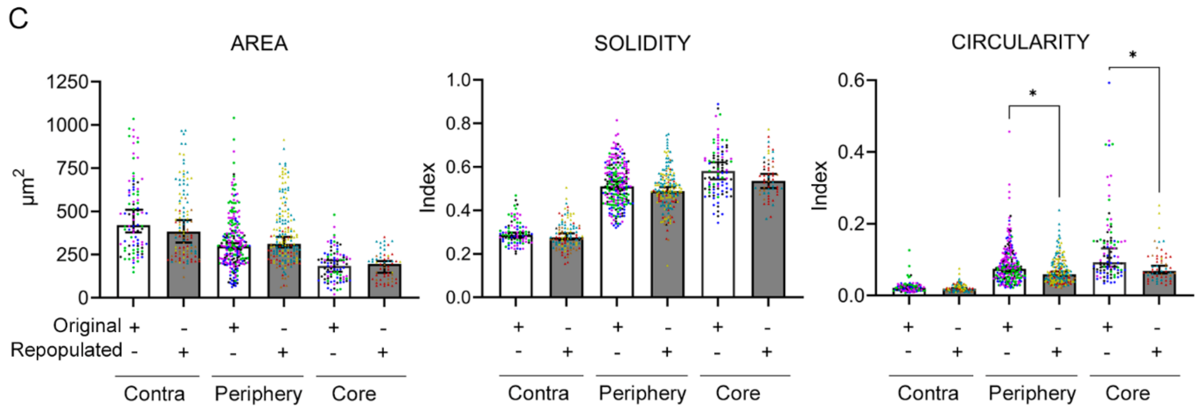

We added the following information in the text,

Methods (page 21):

'Morphometric analysis of microglia

Images of microglial cells were obtained 4 days post-ischemia from P2RY12 immunostained vibratome sections of mice previously subjected or not to microglia depletion/repopulation (n=4 male mice per group). Images were obtained with an oil x60 objective of a confocal microscope (Dragonfly, Andor).

Maximum intensity projections from 21 Z plans were generated with Fiji. The plugin 'New Microglia Segmentation and Tracking' available at the Fiji update site "Microglia Morphometry" (https://github.com/embl-cba/microglia-morphometry#microglia-morphometry) was used for semi-automated segmentation of microglial cells. We analized the morphological descriptors: area, circularity and solidity using Fiji in the ischemic core and periphery and the contralateral hemisphere. For these respective regions, the total number of cells analyzed were 96, 233, and 91 in the group with original microglia, and 58, 178, and 109 in the group with repopulated microglia, respectively.'

Results (end of page 5):
'Morphometric analysis of striatal microglia showed no differences between original and renewed microglia in the contralateral hemisphere. In the ischemic hemisphere, microglia of both groups showed lower area and higher circularity at the periphery and core of infarction versus the contralateral hemisphere, as expected. However, the increase in circularity at the periphery of infarction was less pronounced in repopulated microglia versus original microglia (Fig. EV3), suggesting that morphological changes in response to ischemia were slightly attenuated in the repopulated microglia.'

****
Referee Cross-commenting:

Referee#3
I agree with the Editor that points #5 and #6 can be textually addressed, but in addition to points #1-4, there is also point #7 regarding the (dis)similarity of original vs renewed microglia. I am keen to hear Referee #1 input.
I believe we have answered all the points

Referee #1
I agree with Referee#3, these are valid concerns that need to be addressed.
As editor & R#3 rightly mentioned, some of these concerns can be addressed by rewording, but others may need additional experiments.

Here are some suggestions to address these concerns:
#1. As age and stroke both increase Bodipy+ microglia numbers and amount of lipid droplets there may be a ceiling effect (if this is the case authors should at least discuss this), but to better understand, authors should test for stroke effect in aged mice using later timepoints (day 7 or day 14?) and by using sham brains as controls instead of contralateral hemisphere.

We performed new groups of sham-operated mice as suggested. Please see the above responses. We believe they clarify that ischemia is able to increase lipid droplet-rich microglia even in old mice that already have basal accumulation of lipid droplets under sham conditions. However, the increase induced by ischemia vs. sham is larger (8.3-fold) in young mice than in old mice (2.7-fold).

Regarding studying old mice at day 7 and 14 post-ischemia, it is always nice to see the time course evolution, but please notice that the main point of our study is that when we remove the microglia of old mice, the new microglia repopulating the brain showed reduced lipid deposits typical of old microglia and reduced innate immune responses to brain ischemia.

We added the following text in the Discussion (bottom of page 9 and top of page10):

'While acute lipid-droplet biogenesis in microglia after ischemia resembles the homeostatic response to immunometabolic challenges,[29-30] chronic accumulation of lipids and lipofucsins in microglia of old mice is related to aging and can impair microglia function.[8-11] Microglia renewal in old mice reduced age-associated lipid droplets and favored motor function recovery after stroke.'

#2. Contralateral hemisphere of a post-stroke animal is not the best control, authors should use a sham cohort to at least validate key findings by comparing with stroke brains.

We did sham-operated mice as explained above in response to comment nº 2 of Reviewer 3.

#3. Lack of rational for pooling different timepoints is a weakness. Also, as contralateral hemisphere is not an appropriate control (provided data is not sufficient to confirm, if there is an increase of PLIN2 in ipsilateral hemisphere or a decrease in contralateral hemisphere?), this needs to be confirmed using a sham cohort. A more specific experiment would be to validate if this increase of PLIN2 in stroke brains is significant compared to sham (either at day-4 or day-14, based on human data, it appears that PLIN2 levels remain elevated for several days after stroke).

We did more mice at the different time points after ischemia, and we also performed sham-operated mice for Western Blot analysis. Please see response to comment number 3 of Reviewer 3.

#4. Authors should adopt statistical analysis consistently between behavioral tests. Current analysis is not uniform between tests. Analysis by repeated measures is a more specific statistical test for accessing early recovery.

We did a repeated measure analysis design for the neuroscore using the Kruskal-Wallis test given the non-parametric nature of the neuroscore. Please see our response to comment number 4 of Reviewer 3.

#7. To better support authors' statements, quantification analysis needs to be performed on IHC images by using image analysis tools or by flow using a separate cohort.

As responded above, we carried out more experiments and quantified the IHC using morphometric analysis.

29th Nov 2022

Thank you for the submission of your revised manuscript to EMBO Molecular Medicine. We have received the enclosed report from the referee who was asked to re-assess it. As you will see, the referee is now supportive, and I am pleased to inform you that we will be able to accept your manuscript pending the following amendments.

1. In the main manuscript file, please remove the red color font.

2. Reduce the keyword number to five.

3. Remove the author contribution section from the manuscript file.

4. Appendix: "Supplementary Fig Sx" should be renamed to "Appendix Figure Sx". Please update the Table of Content accordingly.

5. We updated our journal's competing interests policy in January 2022 and request authors to consider both actual and perceived competing interests. Please review the policy https://www.embopress.org/competing-interests and update your competing interests if necessary.
Please use the heading "Disclosure statement and competing interests".

6. Data availability: GSE196737 and GSE209732 are currently private. Please make sure they will be made publicly available upon the acceptance of the manuscript.

7. EV datasets: please add titles to the Datasets EV6 and EV7.

8. None of the EV figures are called out in the manuscript text. Please fix this.

9. Place the Materials and Methods section after the Discussion.

10. Every published paper includes a 'Synopsis' to further enhance discoverability. Synopses are displayed on the journal webpage and are freely accessible to all readers. They include a short stand first (maximum of 300 characters, including space) as well as 2-5 one-sentence bullet points that summarizes the paper. Please write the bullet points to summarize the key NEW findings. They should be designed to be complementary to the abstract - i.e. not repeat the same text. We encourage the inclusion of key acronyms and quantitative information (maximum of 30 words / bullet point). Please use the passive voice. Please attach these in a separate file or send them by email, we will incorporate them accordingly.

11. The paper explained: EMBO Molecular Medicine articles are accompanied by a summary of the articles to emphasize the major findings in the paper and their medical implications for the non-specialist reader. Please provide a draft summary of your article highlighting

- the medical issue you are addressing,

- the results obtained and

- their clinical impact.

This may be edited to ensure that readers understand the significance and context of the research. Please refer to any of our published articles for an example.

12. The references must be formatted according to the EMBO Molecular Medicine reference style.
- Please list up to 10 co-authors of a paper before adding et al. to the reference list.
- Citations should be listed in alphabetical order.
- In the text of the manuscript, a reference should be cited by the author and year of publication.

13. Our data editors will have a look at the manuscript, and they will probably make additional comments and suggestions that need answering. Once I receive their edited version, I will forward it to you.

14. As part of the EMBO Publications transparent editorial process initiative (see our Editorial at http://embomolmed.embopress.org/content/2/9/329), EMBO Molecular Medicine will publish online a Review Process File (RPF) to accompany accepted manuscripts.

In the event of acceptance, this file will be published in conjunction with your paper and will include the anonymous referee reports, your point-by-point response, and all pertinent correspondence relating to the manuscript. Let us know if you DISAGREE with this or if you want to remove or keep any figures from it before publication.

Please note that the Authors checklist will be published at the end of the RPF.

I look forward to reading a new revised version of your manuscript as soon as possible.

Kind regards,
Jingyi

Jingyi Hou
Editor
EMBO Molecular Medicine

To submit your manuscript, please follow this link:

https://embomolmed.msubmit.net/cgi-bin/main.plex

***** Reviewer's comments *****

Referee #1 (Comments on Novelty/Model System for Author):

The role of microglial lipid droplet deposits with aging and how this changes stroke recovery is an important question to address.

The study is relatively novel as it elucidates mechanisms of therapeutic importance.

Referee #1 (Remarks for Author):

The authors have been extremely responsive and addressed all of this reviewer's previous concerns.

The authors addressed the minor editorial issues.

1st Dec 2022

Dear Prof. Planas,

I am pleased to inform you that your manuscript is accepted for publication and is now being sent to our publisher to be included in the next available issue of EMBO Molecular Medicine.

We would like to remind you that as part of the EMBO Publications transparent editorial process initiative, EMBO Molecular Medicine will publish a Review Process File online to accompany accepted manuscripts. If you do NOT want the file to be published or would like to exclude figures, please immediately inform the editorial office via e-mail.

Please read below for additional IMPORTANT information regarding your article, its publication and the production process.

Congratulations on your interesting work,

Jingyi

Jingyi Hou
Editor
EMBO Molecular Medicine

Follow us on Twitter @EmboMolMed
Sign up for eTOCs at embopress.org/alertsfeeds

EMBO Press Author Checklist

Corresponding Author Name: Anna M. Planas	
Journal Submitted to: EMBO Molecular Medicine	
Manuscript Number: EMM-2022-17175	

USEFUL LINKS FOR COMPLETING THIS FORM
The EMBO Journal - Author Guidelines
EMBO Reports - Author Guidelines
Molecular Systems Biology - Author Guidelines
EMBO Molecular Medicine - Author Guidelines

Reporting Checklist for Life Science Articles (updated January 2022)
This checklist is adapted from Materials Design Analysis Reporting (MDAR) Checklist for Authors. MDAR establishes a minimum set of requirements in transparent reporting in the life sciences (see Statement of Task: 10.31222/osf.io/9sm4x). Please follow the journal's guidelines in preparing your manuscript.
Please note that a copy of this checklist will be published alongside your article.

Abridged guidelines for figures
1. Data
The data shown in figures should satisfy the following conditions:
- the data were obtained and processed according to the field's best practice and are presented to reflect the results of the experiments in an accurate and unbiased manner.
- ideally, figure panels should include only measurements that are directly comparable to each other and obtained with the same assay.
- plots include clearly labeled error bars for independent experiments and sample sizes. Unless justified, error bars should not be shown for technical replicates.
- if n<5, the individual data points from each experiment should be plotted. Any statistical test employed should be justified.
- Source Data should be included to report the data underlying figures according to the guidelines set out in the authorship guidelines on Data Presentation.

2. Captions
Each figure caption should contain the following information, for each panel where they are relevant:
- a specification of the experimental system investigated (eg cell line, species name).
- the assay(s) and method(s) used to carry out the reported observations and measurements.
- an explicit mention of the biological and chemical entity(ies) that are being measured.
- an explicit mention of the biological and chemical entity(ies) that are altered/varied/perturbed in a controlled manner.
- the exact sample size (n) for each experimental group/condition, given as a number, not a range;
- a description of the sample collection allowing the reader to understand whether the samples represent technical or biological replicates (including how many animals, litters, cultures, etc.).
- a statement of how many times the experiment shown was independently replicated in the laboratory.
- definitions of statistical methods and measures:
  - common tests, such as t-test (please specify whether paired vs. unpaired), simple χ2 tests, Wilcoxon and Mann-Whitney tests, can be unambiguously identified by name only, but more complex techniques should be described in the methods section;
  - are tests one-sided or two-sided?
  - are there adjustments for multiple comparisons?
  - exact statistical test results, e.g., P values = x but not P values < x;
  - definition of 'center values' as median or average;
  - definition of error bars as s.d. or s.e.m.

Please complete ALL of the questions below.
Select "Not Applicable" only when the requested information is not relevant for your study.

Materials

Newly Created Materials	Information included in the manuscript?	In which section is the information available? (Reagents and Tools Table, Materials and Methods, Figures, Data Availability Section)
New materials and reagents need to be available; do any restrictions apply?	Not Applicable	

Antibodies	Information included in the manuscript?	In which section is the information available? (Reagents and Tools Table, Materials and Methods, Figures, Data Availability Section)
For **antibodies** provide the following information:<br>- Commercial antibodies: RRID (if possible) or supplier name, catalogue number and or/clone number<br>- Non-commercial: RRID or citation	Yes	Materials and Methods section

DNA and RNA sequences	Information included in the manuscript?	In which section is the information available? (Reagents and Tools Table, Materials and Methods, Figures, Data Availability Section)
**Short novel DNA or RNA including primers, probes**: provide the sequences.	Not Applicable	

Cell materials	Information included in the manuscript?	In which section is the information available? (Reagents and Tools Table, Materials and Methods, Figures, Data Availability Section)
**Cell lines:** Provide species information, strain. Provide accession number in repository **OR** supplier name, catalog number, clone number, and/**OR** RRID.	Not Applicable	
**Primary cultures:** Provide species, strain, sex of origin, genetic modification status.	Yes	Materials and Methods section
Report if the cell lines were recently **authenticated** (e.g., by STR profiling) and tested for mycoplasma contamination.	Not Applicable	

Experimental animals	Information included in the manuscript?	In which section is the information available? (Reagents and Tools Table, Materials and Methods, Figures, Data Availability Section)
**Laboratory animals or Model organisms:** Provide species, strain, sex, age, genetic modification status. Provide accession number in repository **OR** supplier name, catalog number, clone number, **OR** RRID.	Yes	Materials and Methods section
**Animal observed in or captured from the field:** Provide species, sex, and age where possible.	Not Applicable	
Please detail **housing and husbandry conditions**.	Yes	Materials and Methods section

Plants and microbes	Information included in the manuscript?	In which section is the information available? (Reagents and Tools Table, Materials and Methods, Figures, Data Availability Section)
**Plants:** provide species and strain, ecotype and cultivar where relevant, unique accession number if available, and source (including location for collected wild specimens).	Not Applicable	
**Microbes:** provide species and strain, unique accession number if available, and source.	Not Applicable	

Human research participants	Information included in the manuscript?	In which section is the information available? (Reagents and Tools Table, Materials and Methods, Figures, Data Availability Section)
If collected and within the bounds of privacy constraints report on age, sex and gender or ethnicity for all study participants.	Yes	Methds and Table EV1

Core facilities	Information included in the manuscript?	In which section is the information available? (Reagents and Tools Table, Materials and Methods, Figures, Data Availability Section)
If your work benefited from core facilities, was their service mentioned in the acknowledgments section?	Yes	Acknowledgment

Design

Study protocol	Information included in the manuscript?	In which section is the information available? (Reagents and Tools Table, Materials and Methods, Figures, Data Availability Section)
If study protocol has been **pre-registered, provide DOI in the manuscript**. For clinical trials, provide the trial registration number **OR** cite DOI.	Not Applicable	
Report the **clinical trial registration number** (at ClinicalTrials.gov or equivalent), where applicable.	Not Applicable	

Laboratory protocol	Information included in the manuscript?	In which section is the information available? (Reagents and Tools Table, Materials and Methods, Figures, Data Availability Section)
Provide DOI OR other citation details if **external detailed step-by-step protocols** are available.	Not Applicable	

Experimental study design and statistics	Information included in the manuscript?	In which section is the information available? (Reagents and Tools Table, Materials and Methods, Figures, Data Availability Section)
Include a statement about **sample size** estimate even if no statistical methods were used.	Yes	Materials and Methods section
Were any steps taken to minimize the effects of subjective bias when allocating animals/samples to treatment (e.g. **randomization procedure**)? If yes, have they been described?	Yes	Materials and Methods section
Include a statement about **blinding** even if no blinding was done.	Yes	Materials and Methods section
Describe **inclusion/exclusion criteria** if samples or animals were excluded from the analysis. Were the criteria pre-established?<br><br>If sample or data points were omitted from analysis, report if this was due to attrition or intentional exclusion and provide justification.	Yes	Materials and Methods section
For every figure, are **statistical tests** justified as appropriate? Do the data meet the assumptions of the tests (e.g., normal distribution)? Describe any methods used to assess it. Is there an estimate of variation within each group of data? Is the variance similar between the groups that are being statistically compared?	Yes	Figure Legends and Materials and Methods section

Sample definition and in-laboratory replication	Information included in the manuscript?	In which section is the information available? (Reagents and Tools Table, Materials and Methods, Figures, Data Availability Section)
In the figure legends: state number of times the experiment was **replicated** in laboratory.	Yes	Figure Legends
In the figure legends: define whether data describe **technical or biological replicates**.	Yes	Figure Legends

Ethics

Ethics	Information included in the manuscript?	In which section is the information available? (Reagents and Tools Table, Materials and Methods, Figures, Data Availability Section)
Studies involving **human participants**: State details of **authority granting ethics approval** (IRB or equivalent committee(s), provide reference number for approval.	Yes	Materials and Methods section
Studies involving **human participants**: Include a statement confirming that **informed consent** was obtained from all subjects and that the experiments conformed to the principles set out in the WMA Declaration of Helsinki and the Department of Health and Human Services Belmont Report.	Yes	
Studies involving **human participants:** For publication of **patient photos**, include a statement confirming that consent to publish was obtained.	Not Applicable	
Studies involving experimental **animals**: State details of **authority granting ethics approval** (IRB or equivalent committee(s), provide reference number for approval. Include a statement of compliance with ethical regulations.	Yes	Materials and Methods section
Studies involving **specimen and field samples:** State if relevant **permits** obtained, provide details of authority approving study; if none were required, explain why.	Not Applicable	

Dual Use Research of Concern (DURC)	Information included in the manuscript?	In which section is the information available? (Reagents and Tools Table, Materials and Methods, Figures, Data Availability Section)
Could your study fall under dual use research restrictions? Please check biosecurity documents and list of **select agents and toxins** (CDC): https://www.selectagents.gov/sat/list.htm	Not Applicable	
If you used a select agent, is the security level of the lab appropriate and reported in the manuscript?	Not Applicable	
If a study is subject to dual use research of concern regulations, is the name of the **authority granting approval and reference number** for the regulatory approval provided in the manuscript?	Not Applicable	

Reporting

The MDAR framework recommends adoption of discipline-specific guidelines, established and endorsed through community initiatives. Journals have their own policy about requiring specific guidelines and recommendations to complement MDAR.

Adherence to community standards	Information included in the manuscript?	In which section is the information available? (Reagents and Tools Table, Materials and Methods, Figures, Data Availability Section)
State if relevant guidelines or checklists (e.g., **ICMJE, MIBBI, ARRIVE, PRISMA**) have been followed or provided.	Yes	Materials and Methods section
For **tumor marker prognostic studies,** we recommend that you follow the **REMARK** reporting guidelines (see link list at top right). See author guidelines, under 'Reporting Guidelines'. Please confirm you have followed these guidelines.	Not Applicable	
For **phase II and III randomized controlled trials**, please refer to the **CONSORT** flow diagram (see link list at top right) and submit the CONSORT checklist (see link list at top right) with your submission. See author guidelines, under 'Reporting Guidelines'. Please confirm you have submitted this list.	Not Applicable	

Data Availability

Data availability	Information included in the manuscript?	In which section is the information available? (Reagents and Tools Table, Materials and Methods, Figures, Data Availability Section)
Have **primary datasets** been deposited according to the journal's guidelines (see 'Data Deposition' section) and the respective accession numbers provided in the Data Availability Section?	Yes	Materials and Methods section
Were **human clinical and genomic datasets** deposited in a public access-controlled repository in accordance to ethical obligations to the patients and to the applicable consent agreement?	Not Applicable	
Are **computational models** that are central and integral to a study available without restrictions in a machine-readable form? Were the relevant accession numbers or links provided?	Not Applicable	
If publicly available data were reused, provide the respective **data citations in the reference list**.	Not Applicable	
